# DMTO: a realistic ontology for standard diabetes mellitus treatment

**DOI:** 10.1186/s13326-018-0176-y

**Published:** 2018-02-06

**Authors:** Shaker El-Sappagh, Daehan Kwak, Farman Ali, Kyung-Sup Kwak

**Affiliations:** 1 Information Systems Department, Faculty of Computers and Informatics, Benha University, Banha Mansura Road, Meit Ghamr - Benha, Banha, Al Qalyubia Governorate 3000-104 Egypt; 2 Department of Computer Science, Kean University, Union, NJ 07083 USA; 3 Department of Information and Communication Engineering, Inha University, 100 Inharo, Nam-gu, Incheon, 22212 South Korea

**Keywords:** Clinical decision support system, Treatment plan, Ontology, Knowledge modeling, Diabetes mellitus

## Abstract

**Background:**

Treatment of type 2 diabetes mellitus (T2DM) is a complex problem. A clinical decision support system (CDSS) based on massive and distributed electronic health record data can facilitate the automation of this process and enhance its accuracy. The most important component of any CDSS is its knowledge base. This knowledge base can be formulated using ontologies. The formal description logic of ontology supports the inference of hidden knowledge. Building a complete, coherent, consistent, interoperable, and sharable ontology is a challenge.

**Results:**

This paper introduces the first version of the newly constructed Diabetes Mellitus Treatment Ontology (DMTO) as a basis for shared-semantics, domain-specific, standard, machine-readable, and interoperable knowledge relevant to T2DM treatment. It is a comprehensive ontology and provides the highest coverage and the most complete picture of coded knowledge about T2DM patients’ current conditions, previous profiles, and T2DM-related aspects, including complications, symptoms, lab tests, interactions, treatment plan (TP) frameworks, and glucose-related diseases and medications. It adheres to the design principles recommended by the Open Biomedical Ontologies Foundry and is based on ontological realism that follows the principles of the Basic Formal Ontology and the Ontology for General Medical Science. DMTO is implemented under Protégé 5.0 in Web Ontology Language (OWL) 2 format and is publicly available through the National Center for Biomedical Ontology’s BioPortal at *http://bioportal.bioontology.org/ontologies/DMTO*. The current version of DMTO includes more than 10,700 classes, 277 relations, 39,425 annotations, 214 semantic rules, and 62,974 axioms. We provide proof of concept for this approach to modeling TPs.

**Conclusion:**

The ontology is able to collect and analyze most features of T2DM as well as customize chronic TPs with the most appropriate drugs, foods, and physical exercises. DMTO is ready to be used as a knowledge base for semantically intelligent and distributed CDSS systems.

**Electronic supplementary material:**

The online version of this article (10.1186/s13326-018-0176-y) contains supplementary material, which is available to authorized users.

## Background

Diabetes is a complex and potentially debilitating chronic disease [1]. It affects many individuals, and represents a global health burden with a financial impact on national healthcare systems [2]. Diabetes has two main clinical categories: type 1 diabetes mellitus (T1DM) and type 2 diabetes mellitus (T2DM). T2DM accounts for 90–95% of new cases. In both conditions, continuous medical care is required to minimize the risk of acute and long-term complications. T1DM can only be treated with insulin, whereas patients with T2DM have a wide range of therapeutic options available, including lifestyle changes and administration of multiple oral and/or injectable anti-diabetes drugs, including insulin [3, 4]. This study concentrates on the non-insulin medications for T2DM, which is a risk factor for cardiovascular diseases and microvascular complications [5].

Lifestyle changes, including a healthy diet, weight loss, increased physical activity, self-monitoring of blood glucose, and diabetes self-management education, can help a patient’s efforts at controlling hyperglycemia. However, they may not be adequate for controlling the disease in the long term, and most patients will require pharmacotherapy intervention to achieve and maintain glycemic control [6]. Individualized choices of medications for patients are a challenge, because the number of medications used to treat diabetes has dramatically increased in the past few years. T2DM patients are usually treated with multiple drugs, and the choice differs according to each patient’s profile [5–7].

The most recent T2DM clinical practice guidelines (CPGs), including those from the American Diabetes Association (ADA) [5], Diabetes Canada (formerly the Canadian Diabetes Association) [8], and the European Association for the Study of Diabetes (EASD), recommend patient-centered and individualized diabetes therapy goals based on life expectancy, duration of diabetes, presence of comorbidities, potential for hypoglycemia or other adverse events, and other profile features [9, 10]. The tailored therapy decision for a specific patient is complex, because these decisions include checking many interrelated symptoms, and choosing from various medications and lifestyle plans [11]. T2DM patients usually take more than one drug, and drug interactions may occur. The risk of harmful drug interactions that can cause hyperglycemia, hypoglycemia, nephropathy, retinopathy, gastroparesis, and sexual dysfunction (among other deleterious effects) increases exponentially as the number of medications in a patient’s regimen increases [12, 13]. Interactions can occur between different T2DM drugs, between drugs and complications from diabetes, between drugs and foods, or between drugs and exercise [13–15]. In addition, the T2DM pathophysiology involves at least seven organs and tissues, including the pancreas, the liver, skeletal muscle, adipose tissue, the brain, the gastrointestinal tract, and the kidneys. Many treatment agents affect the seven organs involved in the pathogenesis of T2DM. Each agent has a mechanism of action on these organs, and each has adverse effects and contraindications. Not every patient with T2DM will respond the same way to a given treatment. The reason might be that physicians do not take all of the patient’s characteristics under consideration, including preferences, comorbidities, and other factors. The treatment that works most often for the greatest number of patients is usually selected first, even though this treatment will not be effective for some patients. No two patients are the same, and consequently, each requires his/her own treatment according to her/his chronic conditions [16]. Generally, T2DM patients need an education plan, a physical exercise and a diet plan, and two to three kinds of oral hypoglycemic agents to control blood glucose [13, 14]. However, it is difficult for general practitioners to determine how to combine these oral hypoglycemic agents, and the incidence of adverse drug reactions will increase if physicians do not carefully consider the drugs prescribed [14, 17]. In addition, because diabetes is a chronic disease, comorbidity usually exists and increases in old age [16, 18–20].

Given this information overflow and that the number of patients is increasing, physicians have no time to study all these data [6]. It is assumed that the accuracy of medical decisions can be enhanced with automated decision-support tools that consider all the patient’s clinical characteristics, treatment preferences, and ancillary data at the point of care [17]. The concept of personalized medication and treatment needs to be achieved. It is an emerging concept for treating diseases, which involves the ability to tailor strategies toward preventing, detecting, treating, or monitoring diabetes in individual patients according to the complete medical profile [5, 9, 21]. This approach would then lead to better outcomes without wasting time on ineffective therapy. In order to get the best treatment for this disease, it is necessary to develop a clinical decision support system (CDSS) for diabetes care recommendations, including suitable diet, physical exercise, education, and drugs [14, 22]. This CDSS must be embedded as a component in the electronic health record (EHR) system. The CDSS can support (1) personalization of therapy; (2) calculation of adverse events, allergies, drug-X interactions (where X can be drug, disease, or food); (3) consideration of the whole patient profile from the EHR, (4) improved medications in hospital and at home; and (5) handling of the most recent CPGs and the most recent advances by periodically updating the knowledge base.

A T2DM CDSS must depend on a complete knowledge base of T2DM diagnoses and treatments. Knowledge sharing, semantic interoperability, integration, and reusability are critical features of any system in the medical domain [23, 24]. An ontology can achieve these objectives [2, 14, 25, 26]. As a result, a formal ontology of T2DM treatment concepts based on recent research and clinical expert opinions is the first step toward implementing a semantically intelligent CDSS [27]. There are many definitions of ontology in the literature. They can be formal and explicit representations of a shared conceptualization [28]. The computer science view of an ontology focuses on the logical consistency and inferential implications of ontologies as sets of assertions. On the other hand, the view of the Open Biomedical Ontologies (OBO) Foundry is that the quality of an ontology is also—indeed primarily—determined by the accuracy with which it represents the pre-existing structure of reality [29]. An ontology, from this perspective, is a representational artifact, comprising a taxonomy as its central backbone, where representations are intended to designate some combination of universals (e.g., human beings), defined classes (e.g., patients), and certain relations between them [30]. Ontologies have been used in many studies, including those on diabetes, to enhance the intelligence and interoperable capabilities of a CDSS [14, 25, 26, 31].

*Regarding diabetes*, there are some diagnosis ontologies, such as the Diabetes Diagnosis Ontology (DDO) proposed by El-Sappagh and Ali [32] as a standard ontology. Diabetes treatment ontologies have received little focus in the literature. Sherimon and Krishnan [33] proposed the OntoDiabetic, an ontology-based CDSS. Chen et al. [14] proposed a recommendation system for diabetes drug selection based on the Web Ontology Language (OWL) ontology and Semantic Web Rule Language (SWRL) rules. Hempo et al. [21], Chalortham et al. [34], and Zhang et al. [35] proposed diabetes treatment ontologies. On the other hand, other studies built diabetes CDSS systems without using an ontology. For example, Tomczak and Gonczarek [36] proposed the eDiab system, which enables a physician to conduct a personalized and detailed medical interview for diabetes treatment based on rules extracted from a data stream. Caballero-Ruiz et al. [22] proposed a web-based telemedicine platform to remotely collect patient features and prescribe diet and insulin needs for patients with gestational diabetes; this system is a classifier based on expectation maximization clustering and a C4.5 decision tree–learning algorithm. However, diabetes treatment complexity and the lack of a gold standard can affect the formalization of knowledge when building a sophisticated CDSS system [22]. Wilkinson et al. [17] evaluated current CDSS systems that provide personalized support for T2DM patients; they asserted that current CDSS systems do not incorporate personalization of treatment goals or treatment selection based on clinical characteristics or patient preferences. A recent study by Donsa et al. [37] found that 37% of patients with diabetes have experienced at least one diabetes medication error during hospitalization, and they asserted that a CDSS can reduce medication prescription errors. Most of the limitations and challenges of non-ontology–based diabetes-treatment CDSSs were collected by Donsa and colleagues [2]. Ontologies can add more power to CDSS systems in relation to semantic intelligence and automation issues [16, 17, 38, 39]; they can be integrated with other reasoning techniques, and can support mobile CDSSs. An ontology can support knowledge sharing, easy maintenance, and reuse in similar domains. Usage of user-defined rules by SWRL adds an extra layer of expressivity to OWL semantics [33]. In addition, an ontology facilitates semantic integration with heterogeneous and distributed EHR environments [40].

*Regarding medical domains other than diabetes*, ontologies have modelled the semantics of many diseases, such as malaria (IDOMAL [41]), cancer (NanoParticle [42]), periodontitis (PeriO [43]), and rehabilitation of knee conditions (TRAK [44]). The following are some examples of ontology-based CDSS systems. Zhang et al. [45] used a set of SWRL rules collected by C4.5 algorithms and an OWL ontology to diagnose mild cognitive impairment. Esposito and Pietro [46] implemented an ontology-based system to help neuroradiologists treating multiple sclerosis, where an ontology represented the semantic structure of expert knowledge and helped to formulate the generated outcomes. Dasmahapatra et al. [47] used an ontology to model the different natures of expertise by describing concepts and relationships in breast cancer. Bouamrane et al. [48] developed a knowledge-based preoperative CDSS system to support health professionals in secondary care during preoperative assessment of a patient prior to elective surgery. Uciteli et al. [49] proposed OntoRiDe, which is ontology-based risk detection software for the whole perioperative treatment process. Hochheiser et al. [50] proposed DeepPhe, a cancer phenotype OWL 2 ontology; this ontology has been used as a knowledge base to build a CDSS for breast cancer. Brochhausen et al. [38] proposed the OBIB-a ontology, which is used in managing biobank information of the Penn Medicine BioBank.

However, no ontology has provided the coverage and completeness required to represent complete semantics for T2DM. Along with coverage of T2DM ontologies, interoperability between T2DM treatment CDSS systems and heterogeneous EHR environments has not been handled in most of previous researches [23, 24]. Researchers have not used any standard terminologies to encode their proposed ontologies. Moreover, most of existing studies failed to deal with the T2DM treatment problem as a chronic and temporal issue [14, 21]. The needed ontology can be incorporated into a healthcare system to automate the process of systematic data collection and the creation process of individualized T2DM treatment plans [33, 51]. As a result, there is a pressing need for a new T2DM ontology for this critical problem.

Our long-term goal is to automate the diabetes treatment process and provide an intelligent and distributed CDSS to be integrated as a component in any heterogeneous and distributed EHR system. Using standard ontologies can support to achieve this goal. However, there is no generalizable ontology in place for the structured storage and retrieval of T2DM treatment plans (TPs) and their associated semantics. In this paper, we describe the detailed process for the development of the Diabetes Mellitus Treatment Ontology (DMTO), an OWL 2 ontology based on SHOIQ (D) description logic. DMTO formally models individualized and standardized T2DM TPs [33]. The resulting ontology is based on standard concepts and relationships from globally accepted medical ontologies, and it contains axioms and rules based on recent diabetes CPGs. DMTO can be used to implement semantically intelligent CDSS systems. These systems can create customized patient treatment plans according to current and historical patient conditions collected from distributed EHRs that include lab tests, symptoms, physical examinations, current comorbidities, currently taken drugs, etc. This plan has to contain three main subplans: customized education, lifestyle, and medications [6]. In addition to information about treatment, this task requires other types of relevant information to be coded, including the current medical knowledge on T2DM complications, symptoms, lab tests, glucose-related diseases, glucose-related medications, drug-drug interactions, drug-disease interactions, drug-food interactions, and diabetes drugs with their characteristics.

Because accurate treatment is based on accurate diagnosis [5, 8], this ontology extends our previously published Diabetes Diagnosis Ontology [32] by adding all T2DM treatment knowledge. Both DDO and DMTO follow the principles of ontology development established by the OBO Foundry (*http://obofoundry.github.io/principles/fp-000-summary.html*). Both are extensions of the Basic Formal Ontology (BFO)^1^ and the Ontology for General Medical Science (OGMS),^2^ and both reuse numerous terms from pre-existing biomedical ontologies and standard terminologies [30]. Sharing development principles and upper ontologies facilitates the subsequent extension of ontologies to achieve broader coverage [38]. BFO is an upper-level ontology designed to support information retrieval, analysis, and integration. BFO enables a realistic approach to ontology modeling in which the classes in an ontology are universal categories of objects that represent things and processes [29, 30]. Unlike DDO, DMTO takes a step toward creating complete and consistent TPs by enabling formal representation and integration of knowledge about treatment drugs, foods, education, lifestyle modifications, drug interactions, the patient profile, the patient’s current conditions, and temporal aspects. The ontology is based on domain expert knowledge, recent research in the literature, books, and the most recent CPGs for diabetes management [5, 8]. Hopefully, DMTO introduces interesting features for sophisticated T2DM treatment plans, and will play a significant role in implementing intelligent, mobile, interoperable, and distributed CDSS systems.

The remainder of this paper is organized as follows. Section 2 illustrates the framework of a CDSS for DMTO. Section 3 details the development process of DMTO. The key features of the proposed ontology, along with results from using it, are discussed in Section 4, whereas Section 5 is a discussion of DMTO and its limitations. Finally, Section 6 concludes this paper.

### The context of the proposed DMTO

**Fig. 1:**
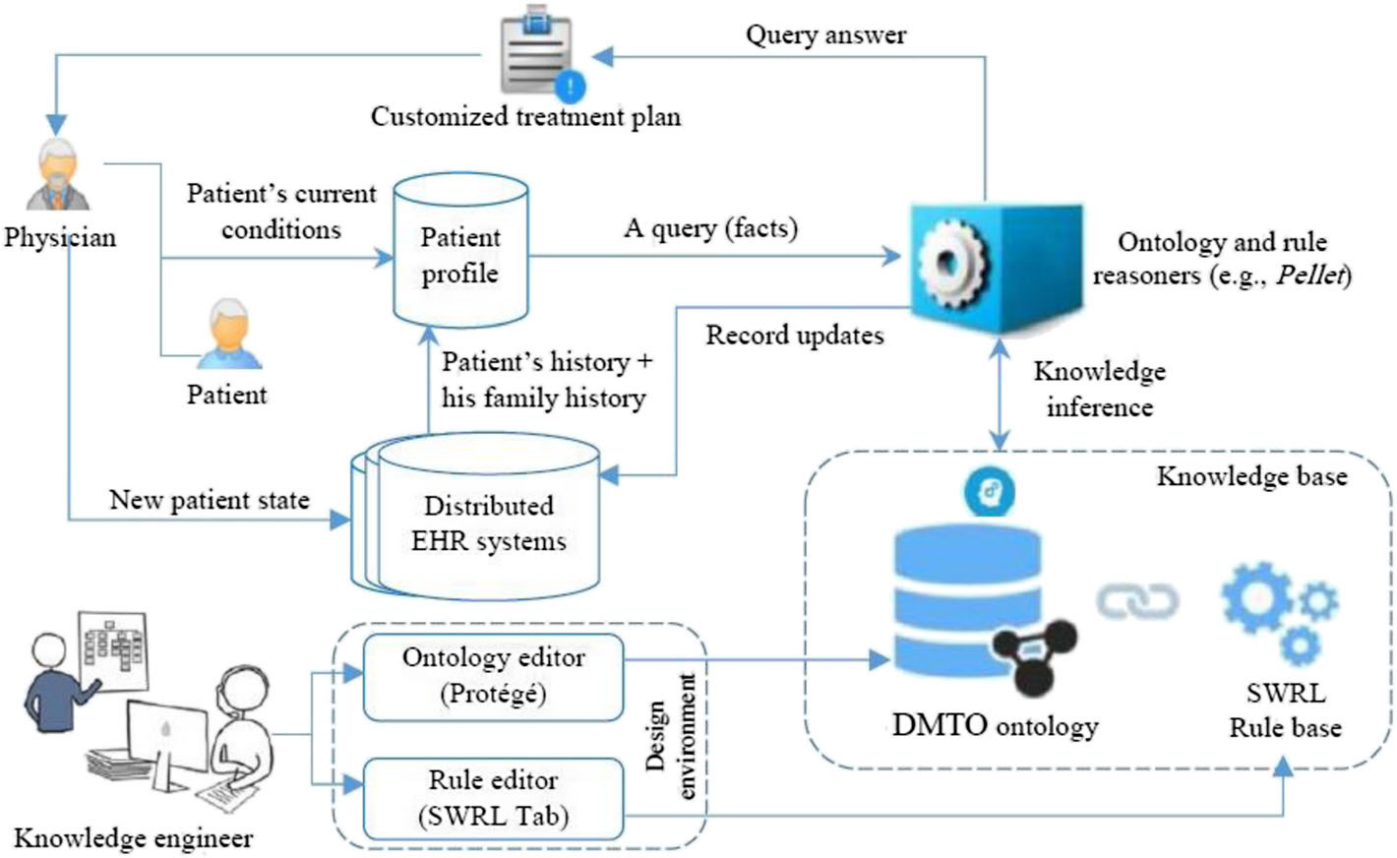
DMTO-based CDSS for diabetes treatment. This figure is the DMTO within the CDSS context. First, knowledge engineers use ontology editors and rule editors to create DMTO in OWL 2 and SWRL formats, respectively. This ontology can be used as a knowledge base for a distributed and semantically intelligent CDSS. Secondly, physicians can interact directly or indirectly with the CDSS through an EHR system. The physician uses the patient profile to query DMTO, which returns a suitable individualized treatment plan. So, DMTO is a core component of an intelligent CDSS

In this section, we discuss the framework of the whole CDSS to illustrate DMTO in its global context. An ontology represents domain knowledge in a machine readable and formal format. As a result, it can be incorporated into a CDSS [31] as a knowledge base. As shown in Section 1, there is an evident gap between the current status and the requirements for implementing an intelligent CDSS for T2DM treatment. The proposed CDSS is capable of automatically collecting patient profiles from distributed EHR systems, and can model TPs using knowledge from a standard medical ontology. Figure 1 shows the main components of the TP recommendation CDSS. The main components are the knowledge base, an inference engine, an ontology management system, and a distributed EHR system.

The knowledge base is implemented using DMTO and Semantic Web Rule Language (SWRL) rules. The ontology management system provides editors for ontology engineers to create and update the ontology and rules. The inference engine is the Pellet ontology reasoner because it supports both ontology and rule reasoning [26, 52]. The EHR system is utilized to complete the patient’s profile, and the resulting decisions can be added to the patient record based on the physician’s decisions. Results from DMTO are compatible with an EHR, because they are encoded using Systematized Nomenclature of Medicine—Clinical Terms (SNOMED CT) standard terminology.

There are many encoding terminologies in the literature. Ahmadian et al. [53] asserted that diversified terminologies adopted by a CDSS and an EHR resulted in problems of semantic interoperability. The paper selected SNOMED CT (SCT) as a unified language for many reasons. SCT has the most comprehensive terminology in the world and provides the most coverage of medical terms [54]. Lee et al. [55] surveyed SCT implementations in terms of design, use, and maintenance issues. Other terminologies are mostly specialized for specific purposes, such as ICD to encode diseases and procedures, LOINC to encode lab tests, and MedDRA to encode adverse events. These other terminologies can be mapped to SCT using many techniques, such as mapping Tables [56]. The metathesaurus of the Unified Medical Language System (UMLS) can be used to bind terms from different terminologies. SCT can be mapped to upper level ontologies like BFO and BioTopLite [57]; it can be used to measure semantic similarities between medical concepts [58]. Many studies proposed OWL 2 formulations for SCT based on description logics [59], but these studies are still not complete as of this writing. SCT has been used with all existing EHR interoperability standards, like HL7 RIM, OpenEHR, and CEN/ISO EN13606 [60, 61], to handle the semantic dimension. Hussain et al. [60] used SCT and HL7 vMR to implement a cloud-based CDSS, and encoded the guidelines using HL7 Arden syntax. Marcos et al. [61] utilized OpenEHR archetypes and SCT to solve the interoperability challenge between an EHR and a CDSS. SCT not only provides a semantic classification of terms but also the possibility of combining terms to describe or refine new healthcare information using post-coordination terms [55]. Finally, SCT has been used in many CDSS systems [60, 61] to improve semantics and support integration within their environments.

The first step is to build the system knowledge base as a formal ontology. We searched the literature, including semantic search engines such as Watson, and searched through biomedical ontologies gathered in the National Center for Biomedical Ontology (NCBO) BioPortal^3^ and OBO^4^ Foundry repositories. The search revealed no ontology strictly dedicated to the construction of detailed, personalized T2DM TPs. As a result, the main focus of this paper is to develop the DMTO knowledge base. In subsequent work, we will implement the complete EHR framework in Fig. 1.

## Method

This section discusses the detailed development process of the DMTO ontology. Before creating DMTO, we surveyed the literature, including databases like ScienceDirect, SpringerLink, and PubMed for any suitable ontologies to be extended into DMTO. The literature was searched from 2010 to February 2017 using the keywords combinations of [“diabetes ontology,” “diabetes semantic,” “diabetes treatment AND OWL,” “diabetes management AND ontology,” “diabetes plan AND ontology,” “diabetes medication AND ontology”]. The only selection criterion was papers that proposed diabetes treatment ontologies. In addition, ontology repositories such as Bioportal^5^ were searched for publicly available ontologies related to diabetes treatment, and we found some, such as disease ontology (DOID) [62], OntoDiabetic [33], DIAB,^6^ etc. However, these ontologies cannot be extended to a global diabetes treatment ontology for many reasons. First, they handle very limited corners of the problem, such as food [63], complications [64], follow-up [35], drugs [14, 65], education [66], diet [67], physical activity [68], and questioners [33]. Secondly, they have not been implemented in a modular way, so the semantics of their terms, relations, and axioms are not clear. Third, they have not been encoded with standard medical terminologies, and their classes have customized semantics (i.e., no top-level, unified semantics). Finally, most of them are not publicly available. We went another way (importing some relevant parts of the public ontologies into DMTO), as will be discussed later.

**Fig. 2:**
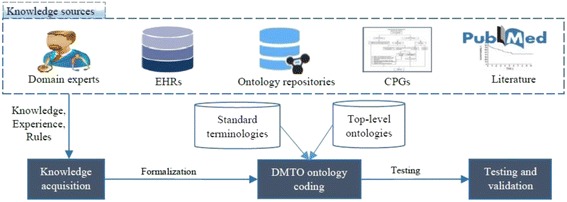
The DMTO construction methodology. This methodology is constructed from a set of sequential steps. The first step is knowledge acquisition to collect the required knowledge from different resources, including domain experts, research in the literature, etc. The second step is DMTO ontology coding, which formulates the ontology and rules in OWL 2 format. The last step is testing and validation, which check the completeness, correctness, and consistency of the resulting ontology. This step is a semi-automated process based on checking ontology content coverage depending on expert knowledge and CPGs, and checking ontology consistency using tools such as ontology reasoners

Figure 2 proposes the steps required to build a complete and consistent ontology. The knowledge sources include the most recent CPGs, relevant T2DM studies, EHRs, and domain experts [1, 5, 11, 33, 51, 69, 70]. This section discusses the DMTO construction methodology, see Fig. 2. It was carried out in four stages that combine scientific evidence with expert clinical opinion. The knowledge extracted from studies in the literature was combined with the knowledge of domain experts and CPGs, and this knowledge was used to create DMTO. The last step was proof of concept and ontology validation. Because DMTO integrates knowledge from different domains (drugs, foods, and others), it is structured in modules. Each module has its specific classes and relationships. Relationships between modules were designed as well. All of these modules are represented under the BFO top-level universals and mapped to SNOMED CT standard terminology to unify the meaning of classes for future extensions, reuse, interoperability, and integration.

### Knowledge acquisition

T2DM management involves the following [5, 11, 69]:

1. T2DM diagnosis of the patient according to current and historic conditions (lab tests, symptoms, physical examinations, family and patient medical histories, etc.)
2. Determination of the patient’s T2DM complications and comorbidities, which complicate medical regimens
3. Review of previous treatments and risk factor control if the patient already has T2DM
4. Creation or updating of a customized and individualized TP (according to patient preferences) that contains subplans for patient education, lifestyle advice (diet and exercise), blood glucose–control drugs, and complications management
5. Provision of a basis for continuing care

The first two items are optimally modeled in our own DDO [32]. DMTO will handle the remaining three. Based on BFO semantics, DMTO arranges its knowledge in the form of modules, and each module is a sub-ontology for a specific type of knowledge. This step collects all relevant knowledge necessary to reliably and completely describe T2DM treatment processes. The knowledge can be generally divided into ontological knowledge (DDO, DIAB, OntoDiabetic [33], RxNorm,^7^ SCT, NDF RT,^8^ FOOD,^9^ TIME,^10^ etc.) and non-ontological knowledge (state-of-the-art medical books [42, 69], relevant scientific papers [11, 33, 51, 71], and CPGs [5]). According to the principles of adequatism defined by Arp et al. [30], we try to collect all possible types of entities and relationships in this domain at multiple levels of granularity to build an adequate ontology. In addition, the DMTO design is based on *realism* and *utility* principles to support building of a complete, consistent, and interoperable ontology [30].

In this phase, the knowledge required to build DMTO comes from four main sources. The first is studies from the literature. Another literature review was conducted on a range of relevant bibliographic databases, including PubMed, SpringerLink, and ScienceDirect. The literature was searched from 2010 to February 2017 using combinations of terms relevant to diabetes treatment, like [“diabetes treatment” AND “drug”], [“diabetes treatment” AND “food”], [“diabetes treatment” AND “plan”], [“diabetes medication”], [“diabetes treatment” AND “process”], [“diabetes mellitus” AND “therapy”], [“diabetes mellitus” AND “diet”], [“diabetes mellitus” AND “lifestyle”]. The titles and abstracts of 200 retrieved articles were screened using inclusion and exclusion criteria. The exclusion criteria included non-English articles, non-medical studies, studies where the searched keywords are not in the title and abstract, and non-T2DM studies. If the full text of the paper is not available, then the paper was excluded. Seventy full-text articles were studied after applying the exclusion criteria. From the reviewed articles, we manually extracted relevant information about T2DM treatment drugs and lifestyles [12, 13, 65, 71, 72], food [63], plans [21, 66, 73], ontologies [33], and rules [74].

The second source is diabetes CPGs. We collected a set of diabetes treatment CPGs, including those from the ADA [5], Diabetes Canada [8], and EASD. Some web sites, including the National Guideline Clearinghouse (*www.guideline.gov*), UpToDate (*www.uptodate.com/home*) and the National Institute for Health and Care Excellence (*www.nice.org.uk*) were searched to collect the relevant structured knowledge for T2DM treatment. These CPGs were studied to extract TP components, relevant concepts and properties, SWRL rules, and the relationships between a patient’s characteristics and a specific plan. The third source of knowledge is official web sites, such as Drugs.com (*www.drugs.com*) and Medscape (*www.medscape.com*). From these sites, we extracted information about T2DM diagnosis criteria and interactions (drug-drug, drug-disease, drug-allergy, drug-food, and disease-food). The fourth source is regular interviews with domain expert. Our domain expert was consulted to clarify the relevant T2DM treatment types, relations, rules, and logic. He validated the collected knowledge and made additions and modifications. In addition, he evaluated the resulting ontology to verify the medical relevance of its decisions. The extracted information provides an initial outline of treatment concepts, conditions, and relationships to be included in DMTO.

### DMTO ontology coding

The information from the previous step is in natural language form. To formally represent collected knowledge, it needs to be refined and converted into an OWL 2 ontology. To support standardization and integration, SCT was searched to obtain identifiers, synonyms, and definitions for each medical class, where such information is available. This knowledge is represented in OWL as annotations. The collected terms from the previous step are represented in a class *is_a* hierarchy using SCT language. Unifying the meaning of each term according to globally accepted terminology facilitates documentation, reporting, integration, and interoperability.

For DMTO to be acceptable as an OBO Foundry ontology, it must (a) adhere to the foundry’s principles, and (b) build further on relevant common architectures or ontologies, which are BFO and OGMS. The approach to DMTO design depends on both the terminological approach, which tries to identify consistent meanings of terms, and the concept-based approach, which focuses on mere logical consistency. This paper depends on our proposed ontology engineering methodology [27]. In the following sections, we will discuss the development steps in detail.

#### Step 1: Determine the domain and scope of the ontology

To determine what is to be included in, excluded from, and the level of granularity in DMTO, this step answers the question, What part of reality is this an ontology of? Because the scope of the ontology is a very important factor that affects its quality and determines its goals, competency questions (CQs) are an essential method in the beginning stages of development to ensure the quality of the ontology. These questions were put to domain experts using very straightforward natural language. They include *general questions* such as:

- ***CQ:*** Why build the DMTO ontology?

***A:*** DMTO will be used as a reference model for the representation of a tailored, personalized, consistent, and complete TP for T2DM patients.

- ***CQ:*** What are the domains this ontology will cover?

***A:*** The ontology will focus on T2DM; however, this methodology can be applied to other domains.

- ***CQ:*** What will this ontology be used for?

***A:*** It is to be used in a knowledge-based system for decision support in hospitals, and in homes for T2DM monitoring and management.

- ***CQ:*** Who are the intended users of the ontology?

***A:*** Diabetologists and medical students.

- ***CQ:*** Is the ontology a brand new one, or an extension of an existing ontology?

***A:*** It is a new ontology, where the domain has no similar one for T2DM treatment.

- ***CQ:*** What will the ontology use to make decisions?

***A:*** The ontology takes into account the patient profile data, including lab tests, symptoms, physical examination results, medications, diseases, patient history, and family history.

- ***CQ:*** What resources will be considered to build the ontology?

***A:*** The most recent advances in T2DM medications, drugs, CPGs, databases, books, research, etc., will be considered. In addition, to provide accurate and acceptable results, it also considers all T2DM complications and medications, associated allergies, foods, education, lifestyles, and interactions (drug-drug, drug-disease, drug-food, etc.).

- ***CQ:*** What is the intended output of this ontology?

***A:*** The ontology generates individualized and tailored chronic treatment plans containing suitable medications, diet, exercise, and education. It can be used as a knowledge base for a CDSS.

In addition, *specific CQs* were asked, such as the following: What are the specific lab tests required, and what is their diagnostic value? What are the T2DM complications, drugs, drug interactions, foods, exercises, etc.? What are the main components of treatment plans? How do you customize a treatment plan according to a patient profile? Asking CQs and modifying the scope of the ontology model are iterated processes. The answers to these questions, guided by the initial motivation, helped the developers to identify the essential information to build this ontology. This step produces the ontology requirements specification document (ORSD).

#### Step 2: Consider reusing existing ontologies

Once we defined the ORSD, we searched for reusable candidate knowledge sources. One of the main benefits of an ontology for knowledge management is its ability to share and exchange knowledge with other ontologies, thanks to the interoperability of OWL. As a result, instead of creating a new ontology from scratch, we had to determine if there is any existing domain ontology or source to extend or refine for the current problem. According to the OBO Foundry principles, reusing existing well-defined ontologies can enhance the new one by avoiding redundancy [75]. In addition, to support standardization, interoperability, reusability, and integration, we required third-party taxonomies and top-level biomedical ontologies to enrich and standardize the classes’ naming and semantics. The list of reused ontologies is in Table 1.

**Table 1:** List of reused ontologies

Full name	Acronym	Ver.	Authors	Location	Main purpose
Diabetes diagnosis ontology	DDO	2015	Shaker El-Sappagh	https://bioportal.bioontology.org/ontologies/DDO	A standardized ontology that collects all of the classes, properties, and axioms of diabetes diagnosis process.
Basic formal ontology	BFO	2015	Barry Smith	https://bioportal.bioontology.org/ontologies/BFO	A genuine top-level ontology used to support the creation of domain ontologies with unified semantics.
Ontology of general medical science	OGMS	2017	Richard Scheuermann	https://bioportal.bioontology.org/ontologies/OGMS	An upper level ontology to uniformly formulate the diseases diagnosis and treatment semantics under the semantics of the BFO ontology.
The Drug-Drug Interactions Ontology	DINTO	2015	Maria Herrero	http://purl.bioontology.org/ontology/DINTO	Describes and categorizes all drug-drug interactions and all the possible mechanisms that can lead to them.
Relation ontology	RO	2017	Chris Mungall	https://bioportal.bioontology.org/ontologies/RELO	A standardized collection of relations or object properties.
Semantic Mining of Activity, Social, and Health data	SMASH	2015	Dejing Dou, Hao Wang	https://bioportal.bioontology.org/ontologies/SMASHPHYSICAL	Describes the semantic features of healthcare data and social networks.
Phenotypic Quality Ontology	PATO	2014	George Gkoutos	https://bioportal.bioontology.org/ontologies/PATO	Models the semantics of phenotypes.
OntoFood ontology	FO	2015	Vikas	http://bioportal.bioontology.org/ontologies/OF	Ontology and SWRL rules of the nutrition of diabetes patients.
Systematized Nomenclature of medicine-clinical terms	SNOMED CT	2017	SNOMED International	https://bioportal.bioontology.org/ontologies/OF	The most comprehensive standard medical terminology.
RxNorm	RxNorm	2017	NLM	https://bioportal.bioontology.org/ontologies/RXNORM	A normalized naming system for generic, branded, and standard drug names, ingredients, and other properties.
OWL Time	TIME	2017	Chris Little Simon Cox	http://purl.bioontology.org/ontology/TIME	Ontology of temporal concepts for describing the temporal properties of resources.

DMTO reuses SCT terminology to standardize the collected concepts. To create an interoperable, rigorous, and clear ontology, all ontology classes are subclasses of BFO and OGMS neutral universals. OGMS supports the modularity property while creating ontologies. Because many terms are imported into DMTO from multiple ontologies (SCT, National Drug File - Reference Terminology [NDF-RT], RxNorm, TIME, etc.), the alignment of all the imported terms was a challenge; we solved it with a carefully designed strategy to manually assert OGMS top-level terms of these imported ontology subsets under the DMTO ontology hierarchical structure. Once the top-level terms were aligned, the middle and bottom level ontology terms were aligned automatically. All of the imported ontologies follow the ontology design principles of the OBO Foundry; as a result, the import process was consistent. The final ontology is domain-specific to represent and study the T2DM treatment process. We used OntoFox (*http://ontofox.hegroup.org**/*) to extract subsets of related terms from different ontologies, including the following:

1. DMTO fully imports the top-level ontologies of BFO and OGMS.
2. The temporal module is represented by importing the World Wide Web Consortium (W3) standard TIME.owl ontology (*https://**www.w3.org/TR/owl-time/*) using Protégé 5.0. All classes and relations of this temporal module are named using the identifier template of *TIME:0000000*. The base class of this module is the *temporal thing* class with an identifier of *TIME:0000002*; this universal is modeled as a subclass of the BFO class *temporal region* with identifier *BFO_0000008.* All data and object properties are modeled under single-parent properties.
3. All T2DM *diagnoses*, *complications*, *lab tests*, *physical examinations*, and *symptoms* are collected from our standard DDO biomedical ontology [32]. DDO is based on BFO and OGMS, as well; DMTO extends DDO by adding treatment knowledge. The DDO types (i.e., classes, properties, axioms, and rules) define the *patient profile* universal. Each *patient* has one current *patient profile* and a historical one to facilitate the monitoring process. This class collects all of the patient characteristics of diagnosis, medications, complications, lab tests, physical examinations, and symptoms. We can assume that the patient profile is a record in a distributed EHR system.
4. Drug *adverse effects* are imported from the Drug-Drug Interactions Ontology (*http://bioportal.bioontology.org/ontologies/DINTO*). All types have the same identifiers as DINTO under the *adverse effect* upper-level class with the *OAE_0000001* identifier. We can add the adverse effect semantics related to all types of drugs, including T2DM treatment and complications, and patient history drugs. In addition, from the same ontology, we added the axioms that define, for each T2DM drug, its adverse effects.
5. For the *drug* class, we reuse the RxNorm, NDF-RT, and SCT ontologies to collect the most suitable lists of drugs, active ingredients, mechanisms of action, contraindications, dosages, side effects, and other critical features. All T2DM treatment drugs were collected, along with their properties. To be self-contained, DMTO had to collect all T2DM drugs related to complications (e.g., statins, fibrates, etc.) and the drugs’ active ingredients. This is because the patient may have one of these complications (e.g., heart failure, kidney disease) but not T2DM. After the patient is diagnosed with T2DM, his new medications must not conflict with the other drugs taken.
6. Three *qualities* are imported from the phenotypic quality ontology (PATO): *quality of a substance* (*PATO:0002198*) to add the semantics of drug qualities, *organismal quality* (*PATO:0001995*) to add the qualities of the patient, and the *process quality* (*PATO:0001236*) to model plan qualities.
7. For T2DM lifestyle planning, we have to prepare a diet plan. This plan requires a recommended mixture of foods. We imported the OntoFood ontology (*http://bioportal.bioontology.org/ontologies/OF*) from the NCBO BioPortal.
8. T2DM patients always suffer from other diseases and take other medications. As a result, their interactions (including drug-drug, drug-food, and drug-disease) are critical when building TPs. We collected this knowledge from three main sources. The first was importing the DINTO sub-ontology related to interactions of T2DM medications. The second was searching RxNorm and SCT for possible interactions related to T2DM drugs. Finally, we used web sites like Drugs.com (*www.drugs.com*) and Medscape (*www.medscape.com*) to confirm the collected interactions and identify new ones.
9. For TPs, the literature is research-poor in ontologies and research. Khoo et al. [39] proposed an abstract treatment plan for general disease treatment. There were no ontologies or research papers specific to T2DM treatment. This version of DMTO only concentrates on T2DM treatment. However, in most cases, when patients are diagnosed with T2DM, it is common to have complications like hypertension that require treatment, as well [18–20]. Extensions of DMTO to handle all T2DM complications are considered for future work.
10. For exercises and lifestyle, DMTO imported some terms related to types of exercise from the Semantic Mining of Activity, Social, and Health (SMASH)^11^ ontology.

#### Step 3: Enumerating important terms in the ontology

This step collects all the terminologies used in the T2DM treatment domain. We selected terms that are familiar and as close as possible to actual usage in the field. To achieve this purpose, we collected the terms from existing ontologies, according to domain experts, and from the most recent and globally accepted diabetes CPGs. Moreover, these terms will be annotated with equivalent terms from the most accepted and popular standard terminology, i.e., SCT. All terms were collected in singular nouns and are rendered in lower case italics. We avoid using acronyms and abbreviations. Each term is assigned a unique alphanumeric identifier. We try to preserve univocity (i.e., each term has exactly one meaning throughout the ontology). This step answers the following questions. What universals and relationships need to be represented? What are the appropriate domain-specific terms that should be used in representing these universals and relations? What levels of granularity for entities are salient for the ontology?

BFO has a set of abstract or high-level classes that can be considered the modules’ parent classes. These classes include *drug*, *disease*, *food*, *TP*, *time*, *interaction*, *lifestyle*, *patient profile*, etc. This step has two main sub-steps, as follows:

##### Identification of relevant terms

All relevant universals and terms are collected from diabetes CPGs, books, existing ontologies and terminologies, and research in the literature. Due to space restrictions, the following are only some of the examples of collected terms:

- For a T2DM *drug* and its associated terms, such as *active ingredient*, *mechanism of action*, *route of administration*, *dose form*, *adverse effect*, etc., we collected all relevant classes from NDF-RT and RxNorm using RxNav (*https://mor.nlm.nih.gov/RxNav/*) and RxClass (*https://mor.nlm.nih.gov/RxClass/*) tools, and from SCT using the CliniClue Xplore tool. SCT T2DM treatment drugs are modeled under the class *384,953,001|antidiabetic preparation*. Each drug has associated annotations including RxNorm’s RxCUI and SCT codes. Because DMTO contains a large number of drugs and diseases, the number of adverse-effect classes collected from the DINTO ontology is large, as well. Interactions were collected from the RxNorm and NDF-RT ontologies in combination with knowledge available in Drugs.com (*www.drugs.com*/), Medscape (*www.medscape.com*), and Epocrates.com (*online.**epocrates.com*) web sites. All T2DM drugs are modeled in DMTO under the *Daibetes drug* (DMTO_0000012) upper class; this class has 9 subclasses: *alpha glucosidase inhibitor* (DMTO_0000914), *biguanide* (DMTO_0000915), *dopamine agonist* (DMTO_0000964), *Incretin* (DMTO_0001582), *insulin* (DMTO_0001413), *meglitinide* (DMTO_0000969), *Sodium-Glucose Transporter 2 (SGLT-2) Inhibitor* (DMTO_0000960), *sulfonylurea* (DMTO_0000917), *thiazolidinedione* (DMTO_0000918).
- Each patient has *patient profiles*. For a patient profile, each patient is described by a set of 12 upper-level classes, including *demographics*, *diagnosis*, *disease*, *symptoms*, *history*, *time interval*, *physical examinations*, *medications*, and *lab tests*. Each class has a number of detailed subclasses. As a result, a specific patient can be connected to many profiles in different periods. This critical feature supports the collection of a patient’s complete history. The patient could be diagnosed as having prediabetes at one time, then gestational diabetes the next time, and then T2DM at another time.

##### Term realization

Defined terms are of two main types: universal and subterm. For each universal, this step determines what entity in reality the term refers to. In other words, it determines the portion of reality described by either BFO or OGMS, where the meaning of the term is associated. All of the DMTO classes subsume BFO and OGMS universals. The following is a very small sample of these classes:

- The *drug* class subsumes the BFO *material entity* class (i.e., DMTO_0000011⊑BFO_0000040). The drug class has four subclasses of *blood glucose lowering drug*, *T2DM complication drug*, *glucose level affecting drug*, and *patient history drug* (DMTO_0000011≡DMTO_0000020⊔DMTO_0000012⊔DDO_0000119⊔DMTO_0000013). The *active ingredient* class subsumes the *chemical entity* class (i.e., CHEBI_59999 ⊑CHEBI_24431). The *chemical entity* class has two main subclasses (CHEBI_24431≡CHEBI_59999⊔DMTO_0001579). The *adverse effect* class is a subclass of the *disposition* class (OAE_0000001⊑BFO_0000016). The mechanism of action (MoA) class subsumes the *role* class in BFO (DMTO_0000078⊑BFO_0000023). The *drug quality* class is a subclass of *quality of substance*, which is a subclass of the BFO *quality* class (DMTO_0000027⊑PATO:0002198⊑BFO_0000019). The drug quality class has subclasses including *administration duration*, *brand name*, *drug A1C lowering level*, *drug weight gain*, *route of administration*, and *drug hypoglycemic risk level*. (DMTO_0000027≡DMTO_0001698⊔DMTO_0001697⊔DMTO_0001693⊔DMTO_0000963⊔DMTO_0000028⊔DMTO_0000973⊔DMTO_0000033⊔DMTO_0000979⊔DMTO_0001585⊔DMTO_0000984⊔DMTO_0001590⊔DMTO_0000029).
- The *patient profile* class is a subtype of the PATO class *organismal quality* (i.e., DMTO_0001670⊑ PATO:0001995⊑BFO_0000019). Each profile is described by a set of classes. All of these classes are detailed in DDO. Regarding patient history information, the *history* class (i.e., BFO_0000182) of BFO represents it. This class has three subclasses: *family history*, *plan history*, and *medication history* (i.e., BFO_0000182 ≡ DMTO_0000913⊔DMTO_0000912⊔DMTO_0000911). The *organismal quality* class has four subclasses: *education*, *lifestyle*, *patient demographic*, and *patient profile* (PATO:0001995≡DMTO_0001702⊔DMTO_0001703⊔DDO_0000124⊔DMTO_0001670).
- The *treatment plan* class is a subtype of BFO’s *occurrent* because it is an entity that has temporal parts, and always depends on some (at least one) *material* entity (i.e., *human* with *patient* role). In DMTO, *treatment plan* is a subclass of the OGMS *planned process* type (i.e., DMTO_0000044⊑DMTO_0002072⊑OBI_0000011)*.* Patient treatment is assigned at a specific time according to the patient profile. After a specific period, the patient profile changes, and accordingly, the patient’s TP changes. As a result, and because T2DM is a chronic disease, the treatment process is a continuous process over the time axis.

#### Step 4: Ontology standardization and encoding

This paper tries to create a standard ontology. To achieve this, we followed two steps. First, DMTO was built as a sub-ontology of a globally accepted top-level ontology, i.e., BFO. This step guarantees the unified meaning of DMTO classes. Secondly, all terms in DMTO are unified with standard terminologies, including RxNorm and SCT. In addition, each class has a set of annotations to more precisely link DMTO with these standard terminologies.

#### Step 5: Define the classes and the class hierarchy

**Fig. 3:**
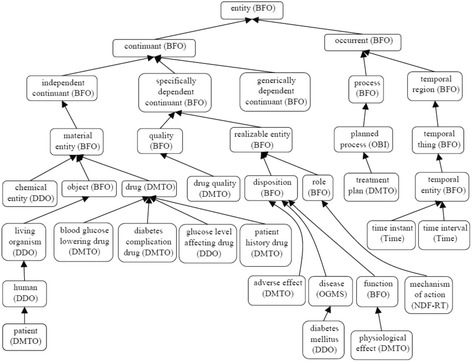
Foundational entities of DMTO in the context of BFO and OGMS. To support the interoperability and sharing capabilities of DMTO, it was designed as a sub-ontology of BFO and OGMS top-level universal ontologies. The main classes of DMTO are implemented under the most suitable upper-level universals of BFO and OGMS according to the semantics of these classes. For example, the DMTO’s *drug* class is implemented as a subclass of BFO’s *material entity* universal. The rectangles are classes, and the black lines are subclass properties

Defined terms are mapped to classes, attributes, and axioms. There are three main methods for identifying the class hierarchy: top-down, bottom-up, and a combination of both. The goal is to develop representational artifacts that are as logically coherent, unambiguous, and realistic as possible. We follow the top-down approach for defining the classes and the *is_a* hierarchy of DMTO. The *is_a* hierarchy is the backbone of every ontology. It is a directed acyclic graph with a single root. Each node is a class, and each edge represents the *is_a* relation connecting a child to its immediate parent. Edges can represent other relations, such as *part_of* as well. We follow the principle of *asserted single inheritance* (see Fig. 3). The *is_a* complete property is to ensure that every class in the domain is in the *is_a* hierarchy of the ontology. This is achieved in DMTO. The definition of classes according to the Aristotelian template guarantees the *is_a* completeness [26]. The general template of term definitions in the ontology hierarchy is as follows:

$$ \boldsymbol{X}=\mathrm{def}.\mathrm{a}\;\boldsymbol{Y}\;\mathrm{that}\;\mathrm{has}\;{\boldsymbol{Z}}_{\boldsymbol{1}},\dots, {\boldsymbol{Z}}_n $$

where ***X*** is the defined universal, ***Y*** is the parent class, and ***Z***_*1*_, …, ***Z***_*n*_ is a set of characteristics or qualities that differentiate the defined term from its parent. For example, *patient* and *treatment plan* can be defined as:
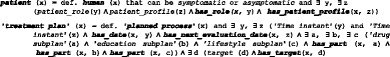


All DMTO classes were defined consistently and in the same manner. Due to space restrictions, and because many classes have complex definitions in DMTO, we will not present other definitions. DMTO was designed in the form of modules, where each module can be considered a separate ontology. Figure 3 shows the top-level structure of DMTO. These modules cooperate to implement the logic of T2DM treatment. Table 2 gives a very short list of the DMTO top-level universals.

**Table 2:** A sample of universals and defined classes

Class name	Description	Class name	Description
symptomatic	A patient who has at least three diabetes symptoms to support the diagnosis.	food	The types of foods that can be used to prepare the diet plan.
diabetes drug	All diabetes treatment drugs, including metformin.	patient	The patient to be diagnosed and treated.
organismal quality	The patient’s qualities, including demographics, profile, lifestyle.	mechanism of action	Models the drug’s mechanism of action.
adverse effect	All adverse effects of diabetes drugs.	treatment plan	Models the patient’s plan, including drugs, lifestyle, and education.

#### Step 6: Define the (object and data) properties of classes

In DMTO, all terms have genus-differentia definitions. In other words, a term is a subclass of a parent term and is distinguished from other related subclass terms (siblings) by some differentiating characteristics unique to that term. Since the class hierarchy itself is not enough to represent domain knowledge, the internal structure of classes has to be considered. Some terms from the glossary were selected as classes; the remaining terms could be represented as relations. Precise definitions of OWL classes cannot be achieved without precise definitions of relationships with other classes. Relations are represented by properties in OWL. There are two types of property: object and data. All properties have associated domains and ranges. DMTO has 170 object properties and 107 data properties. Object properties have formal definitions. For example, the definition of the *has_active_ingredient* property is:

**A** *has_active_ingredient* **B** = def. For every particular **a** of **A**, then there is some particular **b** of **B** such that **a** *has_active_ingredient* **b.**

All object and data properties can be defined this way. Table 3 has a small sample of DMTO object properties. Table 4 has a small sample of DMTO data properties. The Type column in Table 3 and Table 4 indicates if the property will be assigned a value from the EHR or by the physician (Asserted), or if it will be inferred by some SWRL rule. About half of the properties will be inferred by a rule.

**Table 3:** A sample of the implemented object properties

Property name	Domain	Range	Type
contradicte_with_disease	diabetes drug	disease	Inferred
has_role	patient	patient_role	Asserted
has_disease_severity	disease	severity	Asserted
can_be_combined_with	diabetes drug	diabetes drug	Inferred
has_education_program	education subplan	education program	Asserted
has_target	treatment plan	target	Inferred
has_complication	patient profile	patient history disease	Asserted
has_doseForm	drug	dose form	Asserted
has_selected_food	meal	food	Inferred
has_patient_profile	patient	patient profile	Asserted
cause_weight_gain_of	drug	drug weight gain	Inferred
has_measurement_unit	food	measurement unit	Asserted
may_prevent	active ingredient	disease	Asserted
has_history	patient profile	history	Inferred
has_part	treatment plan	drug subplan	Inferred
has_forbidden_food	patient profile	food	Inferred
has_demographic	patient profile	patient demographic	Asserted
contradicte_with_drug	diabetes drug	drug	Inferred
has_diagnosis_severity	diagnosis	severity	Inferred
may_diagnose	active ingredient	disease	Asserted
has_treatment_plan	patient profile	treatment plan	Inferred
has_disease_duration	disease	time interval	Asserted
has_diagnosis	patient profile	diabetes diagnosis	Inferred
has_A1C_lowering_level	drug	drug A1C lowering level	Asserted
has_breakfast_meal	diet	meal	Inferred
has_lab_test	patient profile	diabetes laboratory test	Asserted
has_diabetes_type	diagnosis	diabetes mellitus	Inferred
has_snack_meal_1	diet	meal	Inferred
has_lifestyle_participant	lifestyle subplan	lifestyle	Inferred
has_provider	education program	education provider	Asserted
has_active_ingredient	drug	active ingredient	Asserted

**Table 4:** A sample of the implemented data properties

Property name	Domain	Range	Type
has_length_in_minutes	physical exercise	integer	Inferred
has_next_evaluation_date	treatment plan	date	Asserted
has_expected_decrease_in_A1C	diabetes drug	float	Asserted
can_buy_expensive_drug	patient	Boolean	Inferred
has_amount	food	double	Asserted
has_passed_3_months	treatment plan	Boolean	Inferred
has_glycemic_index	food	string	Asserted
has_total_calories	patient profile	double	Inferred
has_patient_ID	patient	integer	Asserted
has_cost	drug	string	Asserted
has_maximum_dose_per_day	diabetes drug	float	Inferred
has_previous_treatment_plan	plan history	Boolean	Inferred
has_A1C_level	target	float	Asserted
has_basal_metabolic_rate	patient profile	double	Inferred
diabetes_since_date	patient profile	date	Asserted
has_amount_of_calories_for_food	food	float	Inferred
has_date	treatment plan	date	Asserted
has_dose	diabetes drug	float	Asserted
has_severity	diagnosis	float	Inferred
has_social_state	patient profile	{poor, intermediate, rich}	Asserted
has_carbohydrate_grams	meal	double	Inferred
has_calcium	food	double	Asserted
has_sugar_level	target	float	Inferred
has_weight_level	target	float	Inferred
number_of_times	physical exercise	integer	Inferred

**Fig. 4:**
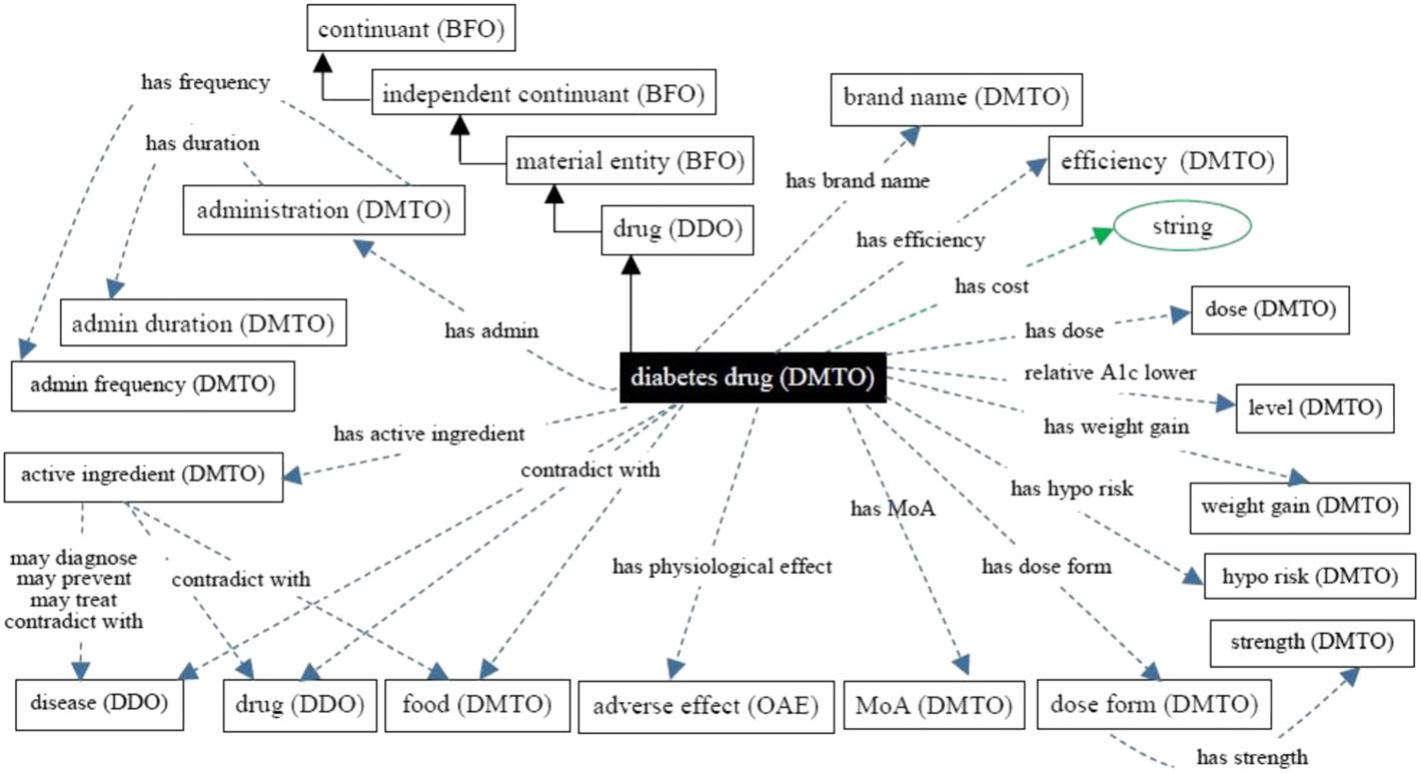
The class diagram of the diabetes drug class. DMTO is a comprehensive ontology of diabetes treatment knowledge. One of the most important classes is the *diabetes drug* class. It is a subclass of the *drug* class, which has other subclasses. The semantics of this class are represented by many object and data properties, such as *contradicte with*, *has MoA*, *has active ingredient*, *has dose*, *has cost*, etc. The green lines indicate data properties, the blue lines indicate object properties, rectangles indicate classes, and the black lines indicate subclass properties

At this point, we built DMTO in three main levels of abstraction. Level 0 is the universals of BFO and OGMS, which unify the semantics of the terms used. Level 1 has three main terms: *time*, *TP*, and *patient profile*. These three classes try to build a *temporal* and personalized *TP* according to a specific *patient profile*. Each universal can be connected to a set of sub-ontologies. Level 2 is the detailed parts for each of the Level 1 classes. Figure 4 is a small fragment of this semantic.

#### Step 7: Define the facets of the slots

**Fig. 5:**
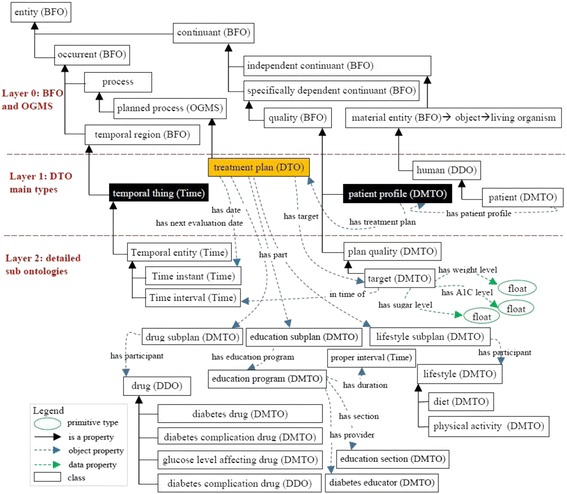
The class diagram of the three levels of DMTO’s main core. DMTO was built as a set of modules. These modules were implemented from scratch or imported from other well-known ontologies. For example, the temporal aspects are imported from the TIME ontology. Building DMTO in layer form supports subsequent maintenance and improvement. The green lines indicate data properties, the blue lines indicate object properties, rectangles are classes, and the black lines are subclass properties

To complete the precise semantics of each universal or defined class, a set of axioms has to be defined. Axioms formulate the logical definitions of types, which support the computational search in DMTO. Some examples of defined axioms are explained next. The *drug* class is defined as:
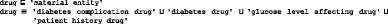


From these classes, we give special manipulations to the *diabetes drug* class because it will be used in defining treatment plans, as shown in Fig. 5. From RxNorm and SCT, we collected all of the properties that can describe this class as follows:
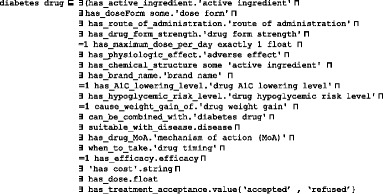

In addition, each subclass of *diabetes drug* has additional characteristics specific to the drug. For example, the *metformin* class, which is the most important diabetes treatment drug, is implemented as follows:
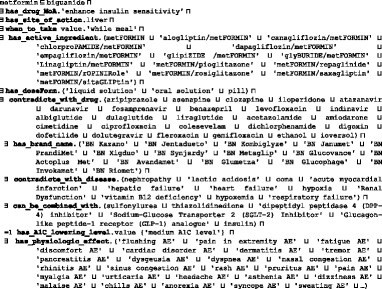


We tried to collect as many characteristics as possible to make the resulting decisions as complete as possible; however, DMTO still requires many improvements based on new knowledge.

**Fig. 6:**
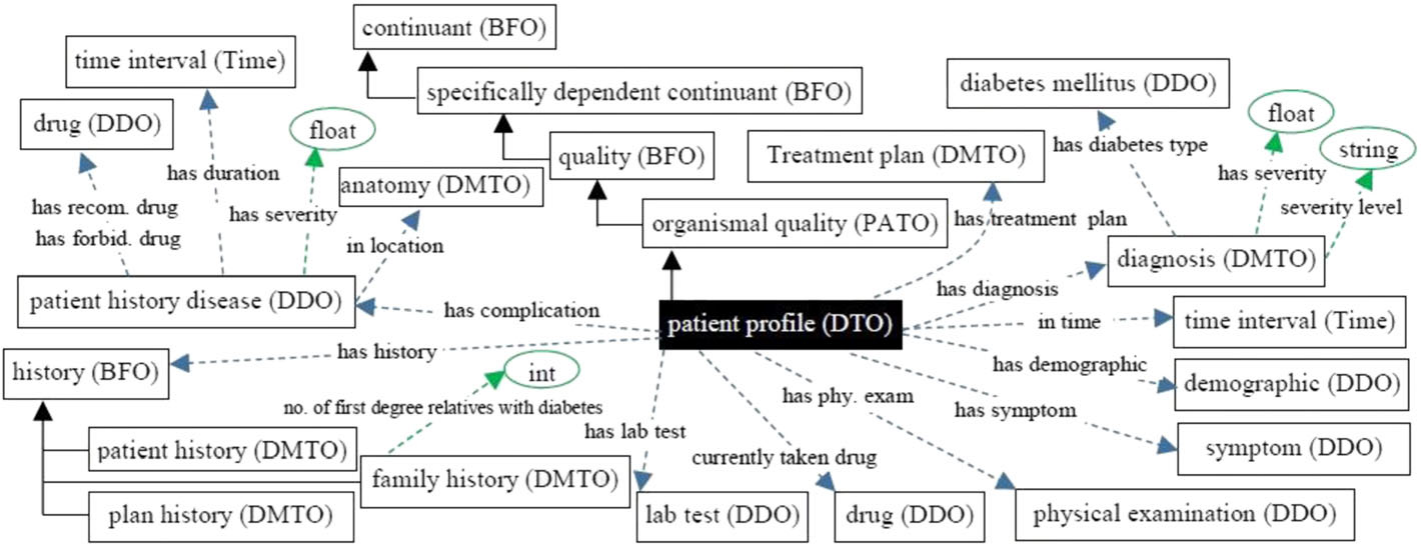
The class diagram of the patient profile class. A patient profile is collected from a distributed EHR environment, because DMTO supports semantic interoperability between a CDSS and an EHR. DMTO collects all of the possible patient characteristics under the *patient profile* top-level class. The green lines indicate data properties, the blue lines indicate object properties, rectangles indicate classes, and the black lines indicate subclass properties

**Fig. 7:**
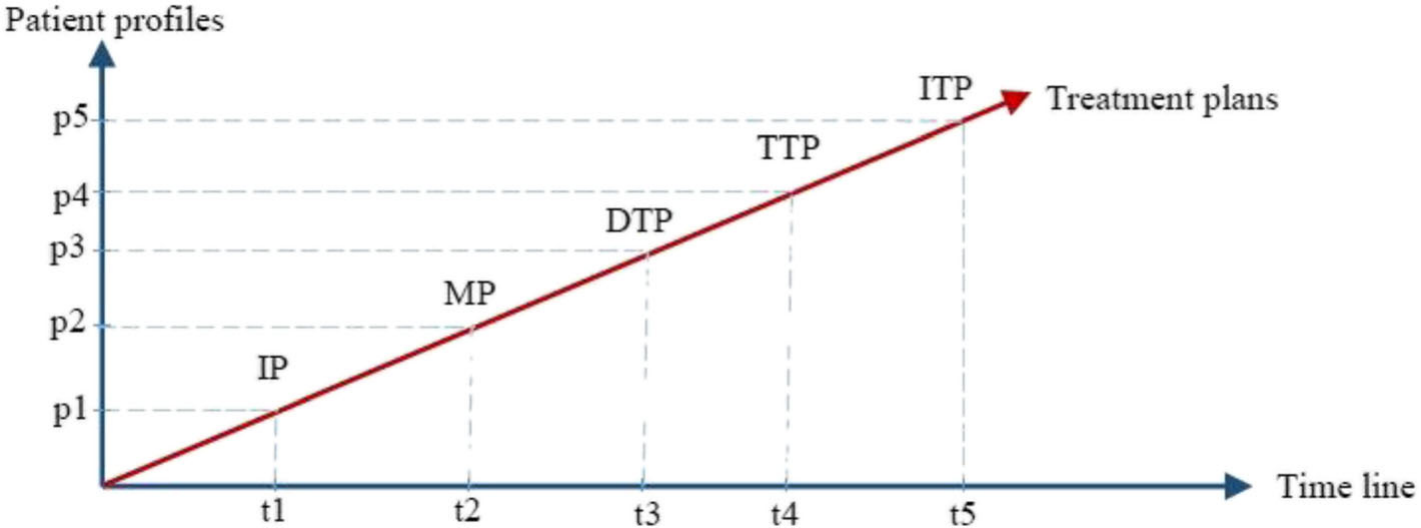
Changes in relationships between patient profiles and treatment plans over time. One treatment plan cannot be utilized for all patients. In addition, the patient’s customized plan cannot be created and forgotten. Regular customization and change is required for a patient plan based on changes in the patient profile over time. As time changes from t1 to t5 (x-axis), the patient profile changes from p1 to p5 (y-axis). As a result, treatment plans change from IP to ITP (the red line)

Let us take another example. The *treatment plan* class is implemented as follows (see Fig. 4):
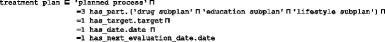


A treatment plan is an action plan. It has subplans that have participants. For example, the drug subplan has participant drugs (Fig. 4). Education and lifestyle subplans have many classes that are not displayed to keep the figure simple. Each plan has a date and a target that defines the plan’s target weight and glucose and A1C levels. The patient profile class collects all of the patient’s EHR features. According to this profile, each patient is assigned a specific plan. This class is represented in Fig. 6 and has the following axioms:
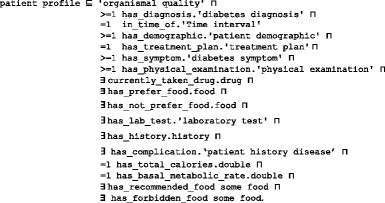

Each patient has at least one profile. If a patient has a treatment plan, then he or she has at least one profile (i.e., the one used to tailor the plan), but not vice versa. The new profile can be for the patient’s current state. The property *has_previous_treatment_plan* is used to determine if the patient has a previous plan. The patient class is designated as follows:
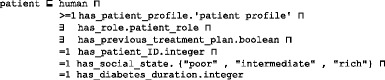


The *patient history disease* class is used to collect the diseases that the patient suffers from. This class is implemented as follows:
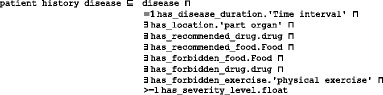


Most classes in DMTO are managed by a set of axioms that specify the complete semantics. As we can see in Fig. 6, we have not added all of the required properties in order to keep the figure simple. T2DM is characterized by ongoing decline in beta cell function, newly diagnosed complications, changes in symptoms, and lab test values. As a result, glucose levels will likely worsen over time. Treatment must be dynamic, because therapeutic requirements increase with longer duration of disease [8]. As shown in Fig. 7 along the timeline, after a specific period (e.g., three months according to the ADA), the patient plan is re-evaluated to check the targets and compare them to the current patient profile. The patient may be assigned a new TP accordingly. Treatment plans have three main subplans: medication, lifestyle, and education. Medication can be categorized into six templates, which can generate 34 plans. Special cases are handled, such as if the patient is *symptomatic* and has *blood glucose > =300 mg/dL* or *HbA1C > =10%*. In this case, we have to consider a combination of injectable insulin therapy. DMTO selects a suitable plan according to the patient’s complete profile. No two drugs from the same category, or that have the same mechanism of action, can be prescribed in the same plan.

If the patient has contraindications with some parts of the selected plan (determined using SWRL rules) then DMTO recommends another component using SWRL rules. Specific examples of these plans can be found elsewhere [3, 5, 7, 8]. A treatment plan can be organized as follows:

1. *Initial plan* (IP) = lifestyle subplan + education subplan. Lifestyle involves diet and physical exercise [11]. The diet component formulates the amounts and types of foods (e.g., *carbohydrates* for 50%–60% of energy intake, *proteins* 1.0–1.2 g/kg/ideal body weight, and *fats* at 7–10%) suitable for the patient based on the profile, including BMI, complications, activity level, glucose level, etc. The physical exercise component determines the types and durations of physical exercise per day and per week that the patient needs to follow according to the profile, including the drugs taken. Education is very important for T2DM patients; as a result, the education subplan provides the patient with sufficient knowledge about T2DM. If HbA1C and blood glucose levels are very high, we can start with medications, including insulin.
2. *Monotherapy plan* (MP): IP + drug subplan of *metformin* (if IP fails in 2–3 months and there are no contraindications for diseases and/or drugs). The *metformin* class subsumes the *biguanide* class, and it has 41 subclasses. If the patient profile shows that the patient has contraindications for metformin, DMTO suggests another suitable drug.
3. *Dual therapy plan* (DTP): MP + drug from other classes. Contraindications for diseases or drugs have to be checked. If the MP failed to achieve the planned goals within two or three months and after increasing the dosage to maximum, then a DTP has to be started. A set of choices is available for the combination of agents, and the choice is made according to the profile. Classes of agents that have different mechanisms of action and that affect different organs should be considered [8]. We have six plans: DTP-1 = MP + sulfonylurea; DTP-2 = MP + thiazolidinedione; DTP-3 = MP + dipeptidyl peptidase inhibitors (DPP-4); DTP-4 = MP + SGLT2; DTP-5 = MP + GLP-1; DTP-6 = MP + insulin (basal). Each plan has its associated characteristics, as shown in Table 5. This knowledge has been implemented in DMTO for every plan type.
4. *Triple therapy plan* (TTP): A set of choices is available, and the choice is made according to the patient’s conditions. We have 25 plans: TTP-1 = DTP-1 + (thiazolidinedione, DPP-4-i, SGLT2-i, GLP-1 receptor agonist, or insulin); TTP-2 = DTP-2 + (sulfonylurea, DPP-4-i, SGLT2-i, GLP-1 receptor agonist, or insulin); TTP-3 = DTP-3 + (sulfonylurea, thiazolidinedione, SGLT2-i, or insulin); TTP-4 = DTP-4 + (sulfonylurea, thiazolidinedione, DPP-4-i, or insulin); TTP-5 = DTP + (sulfonylurea, thiazolidinedione, or insulin); TTP-6 = DTP-6 + (thiazolidinedione, DPP-4-i, SGLT2-i, or GLP-1 receptor agonist). These plans are applied after three months of ineffectiveness from DTP, and if targets are not achieved. The choice of subplan TTP-i for *i = 1, 2, 3, 4, 5, 6*, is based on checking for contraindications for diseases or drugs.
5. *Injectable therapy plan* (ITP): We have two plans: IP + basal insulin + (mealtime insulin, or GLP-1 receptor agonist). If TTP does not achieve the planned targets, then ITP using insulin must be initiated. Due to space restrictions, the current version of DMTO concentrates on oral medications only. Insulin therapy (including basal, long acting, intermediate acting, and short acting) will be discussed as extensions of the current work but in a separate research paper.

**Table 5:** Characteristics of dual therapy plans

	DTP-1	DTP-2	DTP-3	DTP-4	DTP-5	DTP-6
Efficiency	High	High	Intermediate	Intermediate	High	Highest
Hypoglycemic risk	Moderate	Low	Low	Low	Low	High
Weight gain risk	Gain	Gain	Neutral	Loss	Loss	Gain
Side effects	Hypoglycemia	Edema	Rare	GU and dehydration	GI	Hypoglycemia
Cost (user preference)	Low	Low	High	High	High	Variable

An education subplan is required to support the patient for self-management and monitoring of conditions. The *education subplan* class has education programs that have the following semantic axioms. They tailor a treatment program according to the patient’s age, language, disease severity, education level, etc.
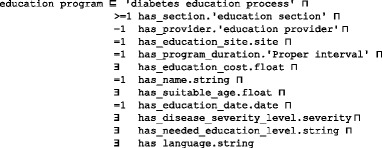


*Education provider* can be a physician, nurse, pharmacist, etc., and they have some demographic data in DMTO. *Education section* is a part of the education program. This design supports the delivery of many types of material (video, text, etc.) and sessions for a patient in a specific program. Each education section is tailored to a specific area in which to educate, such as patient diet, physical activities, or suggested drugs.
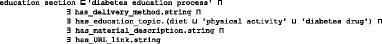


The *lifestyle subplan* has two main parts: *diet* and *physical exercise*. Regarding the diet, we use the following procedure to create customized plans:

- Calculate the ideal calories for the patient according to age, gender, weight, height, and activity level. We use the Harris Benedict Formula [52] to calculate the basal metabolic rate (BMR), or metabolism, in kilocalories per 24 h (kcal/24 h). Given W = weight in kilograms, H = height in centimeters, and A = age in years, calculations are as follows:

1 $$ \mathrm{BMR}\;\mathrm{for}\;\mathrm{men}=66.47+\left(13.75\times \mathrm{W}\right)+\left(5.0\times \mathrm{H}\right)\hbox{-} \left(6.75\times \mathrm{A}\right) $$

2 $$ \mathrm{BMR}\;\mathrm{for}\kern0.17em \mathrm{women}=665.09+\left(9.56\times \mathrm{W}\right)+\left(1.84\times \mathrm{H}\right)\hbox{-} \left(4.67\times \mathrm{A}\right) $$

The *total calories* (TC) per day required to maintain current weight or lose weight are calculated according to the patient’s *activity level* as follows: for little/no exercise, TC = BMR × 1.2; light exercise, TC = BMR × 1.375; moderate exercise, TC=BMR × 1.55; if very active, TC=BMR × 1.725; and if extra active, TC=BMR × 1.9. The activity level is determined according to the physical exercise plan of the patient.

b. TC is distributed into five meals: breakfast, snack 1, lunch, snack 2, and dinner, at 25%, 12.5%, 25%, 12.5%, and 25%, respectively.
c. In each meal, the calorie percentages are divided between the three main nutrients of carbohydrates, fat, and protein at 50%, 30%, and 20%, respectively. DMTO uses SWRL rules to customize the five meals in grams for each nutrient, according to the patient’s specific conditions, where 1 g fat = 9 cal, 1 g protein = 4 cal, and 1 g carbohydrates = 4 cal.
d. In future releases of DMTO, we will aggregate calories for most of the known foods to facilitate the customization of familiar foods and in familiar units (cup, piece, slice, etc.).

DMTO has the needed axioms to support modeling previous knowledge, including SWRL. As an example, the *diet* class has the following semantics:
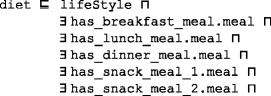


Each *meal* class has the following semantics:
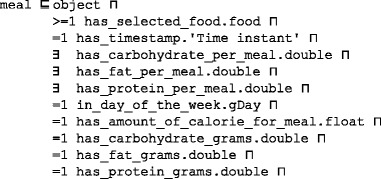


Regarding physical activity in the lifestyle, DMTO facilitates the modeling of many types of exercise, including aerobics, resistance exercise, flexibility activities, and strength activities. For each type, the plan designer can determine the exercise duration and units (i.e., 15 min), frequency and units (e.g., 3 days/week), intensity percentage, progression process, and contraindicated diseases (e.g., aerobics are not allowed for pregnancy with anemia, chronic bronchitis). As asserted by Diabetes Canada [8], physical activities are similar for both children and adults. After defining the required classes and properties, the personalization of exercise programs will be implemented in SWRL rules. Because of space restrictions, full details of the *physical activity* and *physical exercise* classes’ semantics are not provided here, but can be found in DMTO.

#### Step 8: Create instances (populating the ontology with individuals from EHRs)

In ontology terminology, a knowledge base consists of two main parts: the T-box (for terminology) and the A-box (for assertions). Terms for universals are different from terms for instances or particulars. In this step, individual instances of classes are created in the hierarchy. Defining an instance includes choosing a class, creating an individual instance of this class, and populating the values of its defining properties. The set of instances (ABOX) plus the set of semantic rules defined in the next step constitutes the knowledge base of a CDSS to be used by its inference engine (i.e., Pellet reasoner). DMTO is instantiated by the patient’s data from an EHR system. By building the ontology based on BFO and standard SCT terminology, semantic interoperability between the CDSS and EHR systems is achieved. The most important class to populate is the *patient profile*, which collects all of the patient’s temporal medical features, including demographics, complications, history, lab tests, symptoms, etc. In real time, the physician collects the patient’s current features (A1C, weight, blood pressure, etc.) after following a specific treatment plan. The profile plus these data are used to make a new decision about the patient.

#### Step 9: Define SWRL rules

An OWL ontology supports structural inferences, such as subsumption. We use SWRL [76, 77] to encode rules for user-defined reasoning owing to its compatibility with OWL. Many studies in the literature used the numerical capabilities of SWRL to model complex knowledge, and they used reasoners such as Pellet to infer other knowledge [78, 79]. A deductive reasoning capability is required for purposes that are more extensive. The SWRL language standard on top of OWL is based on Rule Markup Language (RuleML). SWRL semantic rules utilize the typical logic expression “antecedent ➔ consequent,” where ➔ means *implies*. The antecedent and consequent are conjunctions of atoms, written as:

$$ {\mathrm{A}}_1,{\mathrm{A}}_2\dots, {\mathrm{A}}_{\mathrm{n}}\to \mathrm{B} $$

where A_*i*_ and B are atomic formulas, where *i* = 1, 2, 3, …, *n*, and “,” is a conjunction. Each atom could be a class, object property, data property, instance, or SWRL built-in. The variables used in atoms are indicated by using a question mark prefix, such as C(?x), OP(?x,? y), DP(?x,? y), and BI (?x_1_,? x_2_,…), where C is class name, OP is object property, DP is data property, and BI is SWRL built-in. In addition, we have access to all XML schema data types. If all the atoms in the antecedent are true, then the consequent must also be true. The consequent part of a triggered rule can be used to update the ontology or issue reminders and alerts. These rules are evaluated according to the patient profiles populated in the previous phase. We organize rules into categories and sub-categories according to the stage at which they are applied in the TP creation process. Table 6 shows some example rules from different rule categories and their triggering conditions. These rules are defined as follows:

- *Patient evaluation rule* (PER): Evaluates a patient’s medical history, symptoms, physical examinations, medications, demographics, and diseases.
- *Patient diagnosis rule* (PDR): Evaluates a patient’s lab test results. This version of the ontology concentrates on T2DM-related rules only, such as HbA1C, FPG, etc. Other lab tests such as lipid profile, urine analysis, kidney tests, and liver function tests will be considered in future work.
- *Plan checking rule* (PCR): Evaluates the patient profile regarding drug, disease, and food interactions.
- *Patient treatment rule* (PTR): Provides suggestions for treatment plans, including medications, lifestyle, and education. The current version of DMTO concentrates on T2DM treatments and does not include treatment of T2DM complications.

**Table 6:**
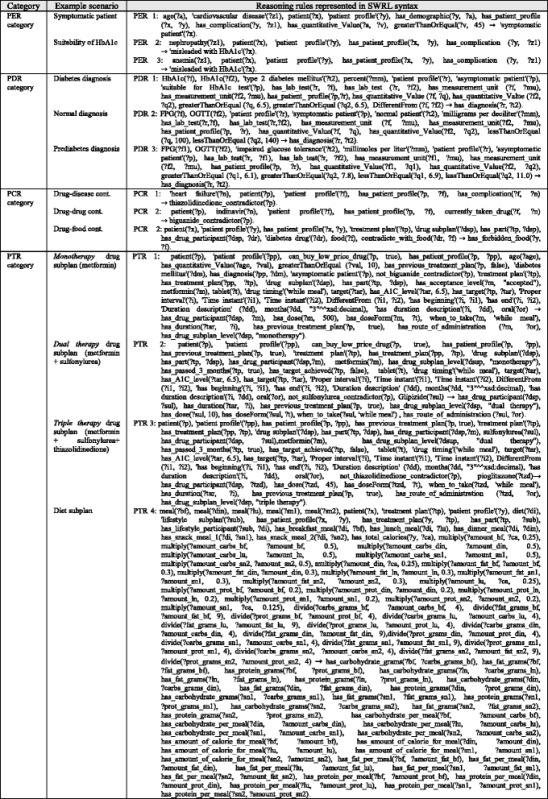
Examples of user-defined personalization SWRL rules

**Fig. 8:**
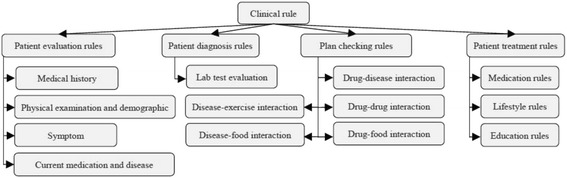
A hierarchy of rules based on clinical knowledge. DMTO has a large number of SWRL rules (i.e., 214 rules) collected from standard CPGs, domain experts, the literature, etc. They are categorized according to the purpose of the rule. There are four main groups, which depend on each other. For example, a patient evaluation rule (PER) evaluates the patient’s symptoms, current medications and diseases, medical history, and physical examination and demographics. The patient diagnosis rule (PDR) determines the diagnosis of the patient according to the previous group and patient lab tests. A patient checking rule (PCR) determines any interactions based on the previous groups, and the patient treatment rule (PTR) suggests a suitable individualized treatment plan

To obtain these rules, we consulted the most recent CPGs, research in the literature, and domain experts [3, 5, 8]. Figure 8 shows this hierarchy, where each category contains a set of rules according to a specific clinical pathway. The output of a stage can be used as input to another stage. For example, PERs classify patients as symptomatic or asymptomatic. Next, PDRs will check whether the patient is symptomatic or asymptomatic while evaluating the lab tests to make the final decision on T2DM diagnosis and severity. These rules are description logic (DL)-safe because they are executed and evaluated based on Pellet reasoner [52].

**Fig. 9:**
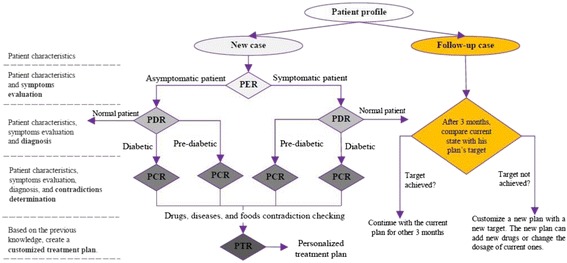
Semantic rules execution sequence. According to the rule groups in Fig. 8, these groups are executed in a certain order to examine the patient profile and determine a suitable plan. A patient case can be a new case without previous plans or a follow-up case with a previous plan. For new cases, the PER group decides if the patient is symptomatic or asymptomatic. Based on the PER, the PDR decides if the patient is diabetic, prediabetic, or normal. The PCR checks drug, food, and disease interactions. Based on the PER, PDR, and PCR, the PTR suggests a personalized treatment plan. On the other hand, if the patient has a previous plan, a check is done after three months. If the target of the current plan is achieved, the patient continues on the current plan for another three months; otherwise, a new customized plan is provided according to the patient’s profile changes and taking into account the patient’s previous plan

The set of defined rules is used to complete the definition of TPs, and they are based on the formalization of diabetes CPGs. Using the SWRL editor plugin in Protégé 5.0, we defined 214 rules to customize a specific treatment plan for a specific patient profile. A full list of these rules can be found in Additional file 1. Figure 9 illustrates the sequence and role of each rule set in the preparation of customized plans. The execution of rules requires the availability of a rule engine that performs reasoning with a set of rules and facts as input. Inferred facts are input to potentially fire more rules (i.e., forward chaining). Our design uses the Pellet reasoner rule engine to enable SWRL reasoning under Protégé 5.0 [52, 80, 81].

#### Step 10: Ontology coding

After specification of the ontology contents and formalization of these contents, the next step is implementation of this knowledge. The Protégé 5.0 ontology editor (http://protege.stanford.edu/) and OWL 2 standard format of the W3C are used to encode DMTO.

### Ontology testing and validation

Ontology evaluation comprises two stages: evaluation of its intrinsic properties (i.e., a technical evaluation) and evaluation of its actual use (i.e., a user’s evaluation). Technical evaluation is the verification and validation of the ontology, which assesses the consistency, correctness, and completeness of the knowledge. Validation of the DMTO terms and axioms against the available CPGs, published research, and books shows that all terms and axioms are valid. We reviewed the ontology and the set of rules with respect to the ORSD in order to detect and solve incompleteness, inconsistencies, and redundancies. This section concentrates on the verification and validation steps. User evaluations reflect the acceptance by end users. This requires designing a complete CDSS connected with an EHR system to study how the combination affects expert decisions. This type of evaluation will be detailed in future work. However, we can consider the usage of case studies and Simple Protocol and Resource Description Framework (RDF) Query Language (SPARQL) queries to verify that the ontology operates as intended. The testing process concentrates on the clarity, adequacy, accuracy, and consistency of the ontology:

1. *Clarity:* All DMTO terms are given a non-ambiguous label using rdfs:label. No abbreviations are used. These understandable labels are based on SCT terminology so the ontology effectively communicates the intended meaning of those terms.
2. *Accuracy and reliability:* DMTO is based on knowledge derived from the most recent and reliable diabetes CPGs under the guidance of medical experts, and no irrelevant terms are used. The ontology tried to import most of its terminology in the form of modules.
3. *Completeness of content coverage:* We evaluated the ontology content against the defined list of CQs. DMTO proved it is 100% complete, as all questions can be answered from DMTO knowledge. Table 7 shows samples of CQs and their corresponding axioms as examples of how the ontology meets these requirements and can answer any question about any specific piece of knowledge about patients. Table 7 lists a complementary and more specific list of CQs than the previously listed CQs in Step 1 of Section 2.2.2. CQs can be represented as queries over DMTO by using SPARQL, DL (description logic) queries, or Semantic Query-Enhanced Web Rule Language (SQWRL) queries. DMTO is the most complete T2DM treatment ontology in the literature. There are no publicly available ontologies that discuss the chronic treatment plan and its parts, the relationship between patient profile and the treatment process, and the standardization of used terminology and upper-level ontologies. DMTO is more flexible and open in order to handle new semantics, because it is based on a modularization concept. Table 8 provides a comparison between DMTO and five diabetes treatment ontologies based on 17 interrelated metrics. As shown in the table, all of the compared ontologies have limited coverage and handle the problem from only a narrow viewpoint. Physicians are expected to not accept their results because of inaccuracy. DMTO is the most complete of the five compared ontologies.
4. *Consistency checking:* This describes a syntactic-level evaluation. The HermiT 1.3.8 [76], Pellet [52], and FaCT++ [82] reasoners were used with the Protégé 5.0 editor to check that DMTO is free of inconsistencies and unsatisfactory classes. They revealed no discrepancies regarding this version of the ontology. Moreover, the Ontology Pitfall Scanner! (OOPS!)^12^ online tool can help to detect some of the 41 most common pitfalls occurring in the development of ontologies. We ran OOPS! on the DMTO ontology and corrected the reported pitfalls. The ontology modules are designed in an OWL 2 format based on SHOIQ (D) description logic. These modules are logically consistent and syntactically correct.

**Table 7:** Competency questions and corresponding axioms in the ontology

#	Competency Question	DMTO-based Axiom
1	Who are all the patients who achieved their treatment plan goals?	patient (?p), ‘treatment plan’(?tp), ‘patient profile’ (?pro), has_patient_profile(?p,?pro), has_treatment_plan(?pro,?tp), has_target_achieved(?tp, “true”^^true), has_passed_3_months(?tp, “true”^^true).
2	Who are the patients who take specific T2DM treatment drug X?	‘patient profile’ (?pro), has_patient_profile(?p,?pro), patient (?p), ‘treatment plan’ (?tp), has_treatment_plan(?pro,?tp), has_part(?tp,?dsub), ‘drug subplan’(?dsub), has_drug_participant(?dsub, X).
3	Who are the patients who have no treatment plan and who have been diagnosed with T2DM?	patient (?p), has_previous_treatment_plan(?p, “false”^^boolean), ‘patient profile’ (?pro), has_patient_profile(?p,?pro), has_diagnosis(?pro, ‘diabetes diagnosis’).
4	Who are the patients who suffer from specific disease X?	‘patient profile’ (?pro), has_patient_profile(?p,?pro), patient (?p), has_complication(?pro, X).

**Table 8:** A comparison between DMTO and some existing diabetes treatment ontologies

Dimension	DMTO	DKOs [21]	Chen et al. [14]	Chalortham et al. [34]	Zhang et al. [35]	OntoDiabetic [33]
Purpose	Treatment	Treatment	Treatment	Treatment	Treatment	Treatment
Available for reuse	Yes	No	No	No	No	No
Based on a unified top-level ontology	Yes	No	No	No	No	No
Encoded using standardized terminology	Yes	No	No	No	Yes	No
Based on OWL 2 and SWRL	Yes	Yes	Yes	Yes	Yes	Yes
Interoperable with EHR systems	Yes	No	No	No	Yes	No
Decisions based on the whole patient profile	Yes	Yes	Only 6 tests entered by user	No	Yes	Yes
Based on standard knowledge (e.g., collected from CPGs)	Yes	No	Yes	No	Yes	Yes
Uses a systematic method for creation	Yes	No	No	Yes	No	No
Delivers treatment plans with drugs, lifestyle, and education	Yes	No	No	No	Yes	No
Models diabetes drugs	Yes	No	Yes	No	No	Yes
Models drugs affecting glucose level	Yes	No	No	No	No	No
Models drug properties	Yes	No	Yes	No	No	No
Models T2DM comorbidities	Yes	Yes	No	No	No	Yes
Reuses existing ontologies	Yes	No	No	Yes	No	Yes
Ontology coverage (number of classes, properties, axioms, and rules)	Table 10	NA	18 drugs +6 rules	NA	NA	NA
Models temporal semantics	Yes	No	Yes	No	No	No

DMTO is publicly available. It can be incorporated into computer systems to facilitate data annotation, decision support, information retrieval, and natural language processing. For example, it can play the role of knowledge base in a T2DM diagnosis and treatment CDSS to support reusability and interoperability. The medical requirements or standards can change continuously. As a result, DMTO requires continuous maintenance to reflect the real environments of T2DM. The maintenance can be done by adding, deleting, or editing new terms, relations, or axioms.

## Results

**Fig. 10:**
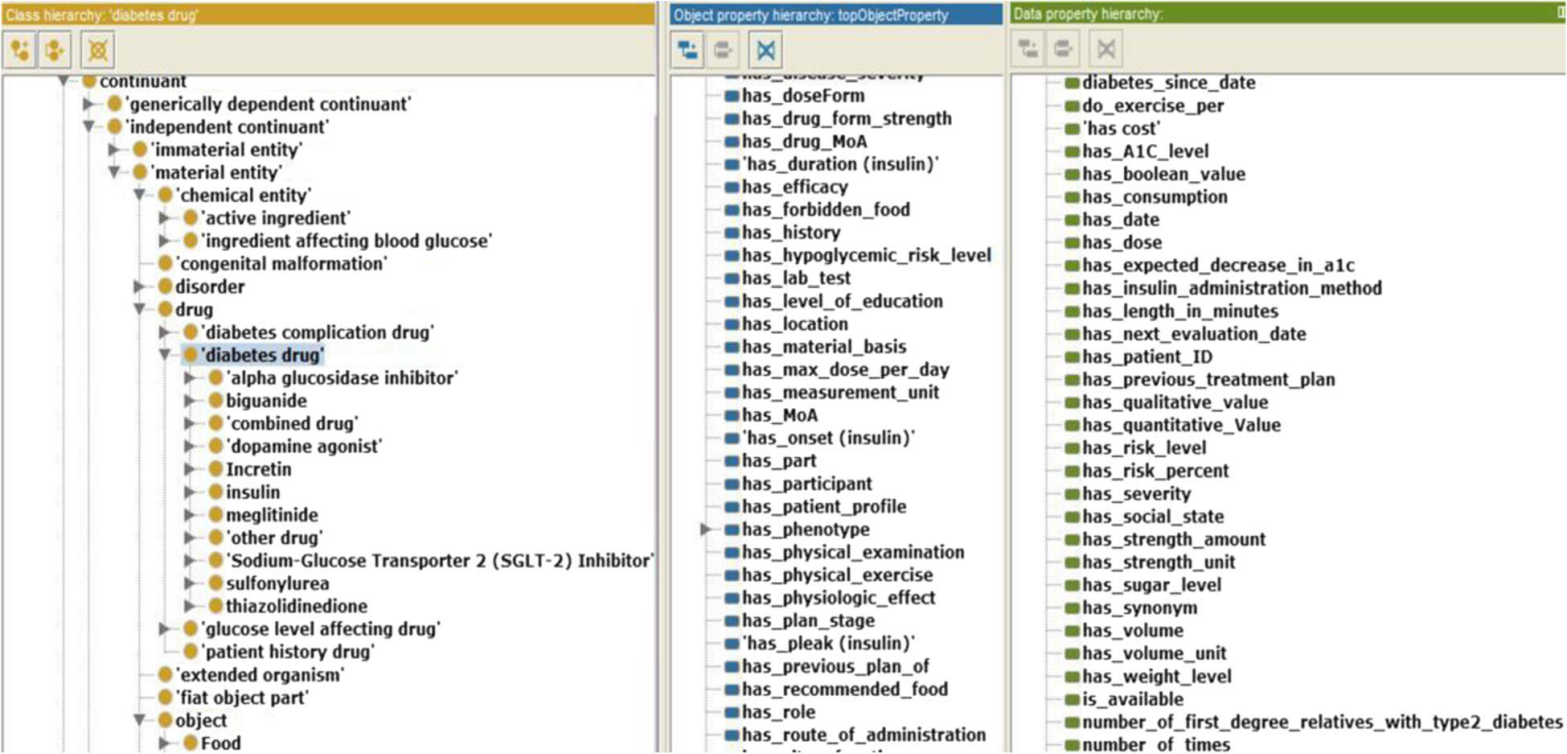
A snapshot of DMTO from the Protégé tool. Protégé 5.0 was used to develop DMTO. The current version of DMTO includes more than 10,700 classes, 277 relations, 39,425 annotations, 214 semantic rules, and 62,974 axioms

In this section, we present the key features of the DMTO ontology. DMTO is encoded in OWL 2 file format by using the Protégé 5.0 tool (*http://protege.stanford.edu**/*). See Fig. 10. This version of DMTO incorporates more than 10,700 classes linked by a total of 170 object properties and 107 data properties. A total of 62,974 axioms have been added into forms in DMTO. Each class is a subclass of an anonymous ancestor, which defines its complete semantics. As a principal in OBO Foundry ontologies, an identifier is always bipartite, in the form of *ID-space_Local-ID*. The *ID*-*space* entries represent the identifiers of ontologies that are used, i.e., DMTO. *Local*-*ID* represents a unique identification number of seven digits. Each class and property has a unique identifier with the format DMTO_0000000. In addition, 214 SWRL rules were added to implement the logic of treatment plans. Classes were annotated with their (preferred) names, definitions, synonyms, and unique identifiers from SCT and RxNorm, with cross-references to other authoritative sources of relevance to this application. DMTO has 39,425 annotation properties. In the current version, not all classes are fully annotated. In terms of coverage of such a complex domain, DMTO is still expected to grow over time. Indeed, few biomedical ontologies can be regarded as totally complete [83]. Through community engagement and feedback, the initial version of DMTO is expected to append other aspects, such as patient history, drugs and diseases, and the management of T2DM complications, which will enhance self-containment, community representativeness, structure, and semantics. In general, an ontology is a global and abstract representation of a domain. Therefore, it does not contain instances or individuals, in most cases. In our design, the ontology contains only classes, properties, axioms, and rules. By using DMTO to build a CDSS, ontology instantiation will be performed according to each set of customized patient conditions and characteristics from EHR environments. Figure 3 depicts the upper-level hierarchy of DMTO with BFO as its backbone. The next-level classes are a combination of DMTO-specific classes and imported classes from other ontologies, including temporal aspects (from TIME); diagnosis, complications, lab tests, physical examinations, and symptoms (from DDO); drug adverse effects (from DINTO); drugs (from RxNorm); and other qualities (from PATO). At lower levels, classes are mainly named based on SCT terminology. Classes are related by the *is_a* relationship. Other relations are used to specify all other relationships between classes. Table 9 illustrates the distribution of imported and newly added classes and properties. It is worth noting that DMTO added only 1015 items out of 10,977 (9.25%). The increased percentage of reuse from an existing stable of 10 ontologies increases the acceptance, shareability, and interoperability of DMTO in the medical domain. This ontology engineering concept is used to model most modern biomedical ontologies [63]. SCT is not in Table 9 because all DMTO class labels and synonyms are initially lexically mapped to SCT concepts.

**Table 9:** External ontologies cross-referenced in DMTO

Ontology	Role in DMTO	Classes	Object property	Data property	Total
BFO	Top-level reference ontology	35	0	0	35
OGMS	Mid-level reference ontology for medical domain	120	0	0	120
RxNorm	References drugs and chemical substances	343	20	5	368
TIME	All temporal related classes and relations	28	28	20	76
DINTO	All drugs’ adverse effects	2369	0	0	2369
DDO	Diabetes diagnosis–related aspects	6444	42	6	6492
OBO RO	Standard object properties design	0	20	0	20
PATO	List of qualities that describe drugs and processes	219	0	0	219
OntoFood	List of diabetes foods and material nutrients	216	0	0	216
SMASH	Terms related to types of physical exercise	47	0	0	47
Total imported		9821	110	31	9962
Newly added		879	60	76	1015
DMTO		10,700	170	107	10,977

We also provide textual definitions for some classes. The structural evaluation of DMTO is shown in Table 10, which lists some metrics regarding its size and composition collected from Protégé according to the Pellet reasoner [52]. In evaluating the correctness of DMTO, we find that it correctly satisfies the defined requirements. The latest version of DMTO in the OWL 2 format is publicly available for download from *http://bioportal.bioontology.org/ontologies/DMTO*. NCBO’s BioPortal is a web portal that supports a uniform mechanism to access biomedical terminologies and ontologies in different representation formats, such as OWL and OBO.

**Table 10:** DMTO ontology metrics

Metric	Value	Metric	Value
Number of classes	10,700	Maximum depth (is_a relationship)	19
Number of object properties	170	Number of annotations	39,425
Number of data properties	107	Number of SWRL rules	214
Maximum number of children	91	Number of axioms	62,974
Average number of children	3	SubClassOf axiom count	11,317
Classes with a single subclass	1109	DisjointClasses axiom count	62
Classes with more than 25 subclasses	31	Logical axiom count	12,264

### Case study

**Fig. 11:**
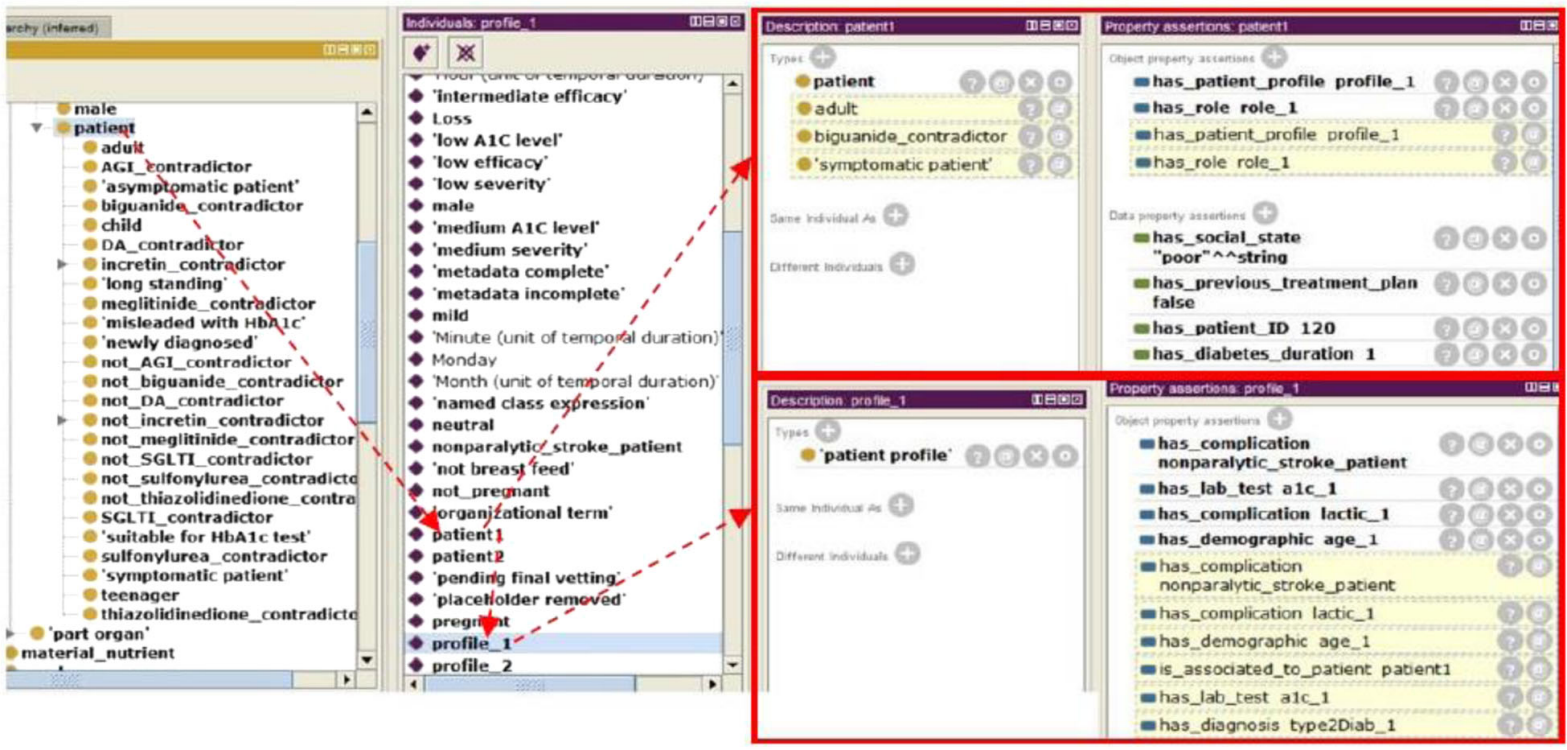
Symptomatic and adult patient inference process. The creation of a personalized treatment plan depends on the OWL 2 asserted and inferred properties. The inference process is done according to OWL 2 axioms and SWRL rules by utilizing the Pellet reasoner. In this case, *patient 1* is an instance of the *patient* class. *Patient 1* includes *profile_1*. *Patient 1* and *profile_1* have a set of asserted properties. Based on the existing axioms and rules, another set of properties is inferred. The upper red rectangle represents the properties of *patient 1*, and the lower red rectangle represents the properties of *profile_1*

Each phase of the development process was evaluated separately to measure its accuracy and completeness. The ontology was previously evaluated via CQs, and here, it is evaluated using a very simple case study to demonstrate the inference sequence. We check a single full path in Fig. 9 where the patient is symptomatic (according to PERs), is then diagnosed with T2DM (according to PDRs), which then shows some contraindications (according to PCRs), and finally, a tailored plan is proposed according to the patient-specific profile (according to PTRs). Each patient in an EHR can be populated automatically in DMTO as a case. A case for a specific patient is created by class instantiation and property assertions from the EHR, with ontology population, OWL axiom inferences, and SWRL inferences (see Table 3 and Table 4). We manually created the case instances and property assertions. This paper depends on the Pellet reasoner because it supports SWRL rules. The patient’s diagnosis and medication follow the Fig. 9 algorithm. A real case can be described as follows:
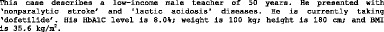


The inference process is done as follows:

**A.** As shown in Fig. 11, the patient gets ID = 120. According to the SWRL rule:
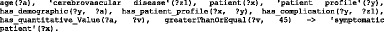

The Pellet reasoner infers that this patient is *symptomatic* and *adult* because *nonparalytic stroke* is a sub-disease of *cerebrovascular* disease and age 50 > 45, respectively. Writing a SPARQL query to directly retrieve symptomatic patients is trivial.

**B.** Figure 11 illustrates that patient_1 has a *biguanide* contraindicator according to two SWRL rules:
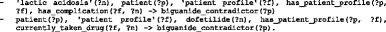


Pellet reasoner infers that this patient cannot take metformin because he has *lactic acidosis* and/or takes *dofetilide*. In addition, the patient is diagnosed with *type 2 diabetes mellitus* according to the following SWRL rule:
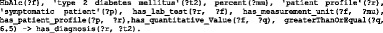


**C.** After deciding that the patient has T2DM, and because the patient has no previous plan, the following rule suggests a monotherapy drug subplan with *glimepiride* from the *sulfonylurea* family after determining that sulfonylurea is not contraindicated. This low-income patient can buy sulfonylurea drugs because they are not expensive.
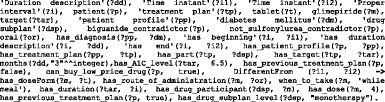


This drug has a suitable dosage form and a route of administration, with times taken, duration, and dose. Regarding diet subplan, *first*, it is created by calculating the patient’s BMR: (Eq. 1) = 66.47 + (13.75 × 100) + (5.0 × 180) - (6.75 × 50) = 2003.97 kcal/24 h. BMR is based on the following SWRL rule:
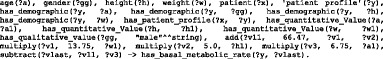


*Secondly*, total calories are calculated for this teacher as follows [84]: TC = 2003.97 × 1.375 = 2755.46 kcal/24 h. This calculation is based on the following SWRL rule:
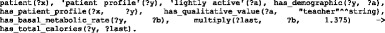


*Finally*, according to SWRL rule PTR 4 in Table 6, the patient is assigned a customized diet subplan as follows:

1. TC for meals are: breakfast = 688.865, snack 1 = 344.4325, lunch = 688.865, snack 2 = 344.4325, and dinner = 688.865.
2. TC for each meal are divided between the three main nutrients of carbohydrates, fats, and proteins. For each of breakfast, lunch, and dinner (carbohydrates = 344.4325, fat = 206.6595, and protein = 137.773); and for each snack (carbohydrates = 172.21625, fat = 103.32975, and protein = 68.8865).
3. For each meal, TC for each nutrient are converted into grams; for example, breakfast is 344.4325/4 = 86 g carbohydrates, 206.6595/9 = 23 g fat, and 137.773/4 = 35 g protein.

DMTO has the ability to capture most patient features collected in an EHR. In addition, it can make semantic inferences to discover hidden patterns. DMTO supports interoperability and integration between distributed CDSSs or EHR systems. In future work, we will handle this interoperability challenge based on available standards, such as openEHR and SNOMED CT.

## Discussion

In this paper, we introduced the development of the DMTO ontology. It provides a standard, robust, and consistent representation and organization of T2DM customized TP knowledge. It can serve as a knowledge base that integrates various aspects of knowledge from domain experts, CPGs, and the literature related to this main topic. DMTO was built with an eye toward improving the development of semantically intelligent and distributed CDSSs embedded as a component of EHR systems. For example, DMTO supports a CDSS to show that *polyradiculopathy* is a *neuropathy*; as a result, it can evaluate the meaning of a patient’s available data, and collect and integrate it from distributed and heterogeneous EHRs. DMTO supports semantic interoperability among different CDSS systems and between a CDSS and an EHR system. To achieve this goal, we made several design decisions pertaining to the use of existing ontologies, upper-level ontologies, standard terminology, and how the ontology is presented in order to best fit the scope and purpose of T2DM treatment. We used a formal methodology to identify relevant terms, identify classes and relations, and add suitable axioms and SWRL rules. To the best of our knowledge, this is the first public repository systematically documenting T2DM management. The DMTO is aligned with BFO 2.0 and OGMS. It imports many terms from existing ontologies and includes many new ontology terms. All terms have unique identifiers, and their labels are extracted from standard terminologies, such as SCT, RxNorm, NDF RT, etc. To evaluate and analyze DMTO’s content and structure, many ontology reasoners were used, and a case study was conducted.

DMTO creates individualized and customized treatment plans. These plans include complete and consistent parts, including drugs, lifestyles, and education. These parts are recommended according to the patient’s current conditions and history. More interestingly, DMTO takes all of the patient conditions into consideration, including lab tests, complications, currently or previously taken drugs, symptoms, family history, etc. The main focus is to provide applicable plans that are acceptable by both domain experts and patients. To create DMTO, we combined a top-down and bottom-up methodology. In terms of the *top-down* method, we first determined the top-level universals from the most applicable top-level ontologies (namely, BFO and OGMS) and made all DMTO terms subclasses of these universals. The determination was done according to the semantics in both. The *bottom-up* method identifies the most specific terms by utilizing diabetes CPGs, domain expert knowledge, existing well-known ontologies, and research in the literature. We imported existing terms (and provided new terms) that support the semantics of treatment plans. Before generating a new term, we check to see if this new term and its possible upper-level term exist in other ontologies, and an extensive discussion is conducted to achieve consensus on a new term’s definition. The prior knowledge of upper-level ontologies and existing ontologies, especially BFO, OGMS, TIME, DINTO, RxNorm, NDF-RT, SCT, PATO, and OntoFood, is essential to the development of DMTO.

DMTO can be used for several applications. First, it can serve as a knowledge base for a T2DM treatment CDSS. It captures complete knowledge extracted from several sources and formulated in OWL 2 axioms and SWRL rules in a consistent manner. Secondly, because DMTO is based on BFO, existing ontologies, and standard terminologies, and owing to the parseable and machine-understandable nature of the ontology, DMTO supports semantic interoperability, data exchange, data integration, and automated reasoning. DMTO knowledge can be integrated with other ontologies, which supports the integration of CDSS systems for different diseases. This offers great benefits, especially in the T2DM domain, because T2DM has so many complications that require treatment at the same time.

Although DMTO is the most comprehensive T2DM treatment ontology, it still has many limitations. These limitations come from the limited availability of detailed medical knowledge in the literature, and the need to narrow the scope of the ontology. We studied most of the existing T2DM treatment CPGs and pathways; however, some of their semantics are not handled in the ontology because these resources contain only summarized knowledge and do not cover all possible conditions. As a result, DMTO will stay open for any new or altered knowledge about T2DM medications. *First*, DMTO concentrates on the treatment of T2DM, because T2DM affects 90% of patients, but T1DM is critical, because it affects children and is mainly treated with insulin, which requires complicated plans and follow-up. *Secondly*, this paper discusses in detail the development process of DMTO. Full testing of this ontology requires connections with EHR systems to populate the ontology with real cases from real environments. *Third*, the ontology does not add the semantics of insulin therapy. *Fourth*, DMTO models diet plans by the percentage of carbohydrates, protein, and fat in each meal. Future enhancements to DMTO will tailor diet plans with familiar foods and with acceptable measurement units, such as cup, piece, etc. In addition, DMTO tries to provide treatment plans for T2DM only; however, a major step in managing T2DM is to manage its complications. Last and most importantly, in the future, we will build a DMTO-based distributed CDSS as an embedded component in an EHR system. DMTO supports integration and interoperability, which facilitates the development process.

## Conclusion

In this paper, we developed a theoretically sound and semantically intelligent T2DM treatment ontology. Such an ontology provides a major step toward the development of more-intelligent CDSSs for chronic disease management. DMTO is based on standard CPGs, standard top-level ontologies, and standard medical terminology, so we consider it a standard ontology. As a result, it can function as the knowledge base of a portable, interoperable, and semantically intelligent CDSS. DMTO is the most comprehensive ontology for T2DM treatment, including all of the needed semantics for treatment plans, T2DM complications, lab tests, symptoms, physical examinations, diet, food, physical exercise, education, drugs, contraindications, etc. All of this knowledge is interrelated in this semantic repository to provide a complete picture of the patient profile and treatment plans and their associated drugs, lifestyles, and education. DMTO includes more than 10,700 classes, 277 relations, 39,425 annotations, 214 semantic rules, and 62,974 axioms. We expect that DMTO will be utilized in the literature to build CDSS systems. DMTO is open and includes an infrastructure for any additions and modifications based on any new requirements or changes in T2DM research. The limitations listed above are the targets of future studies. In addition, handling the vagueness of the medical domain can be accomplished by using a fuzzy ontology. It can enhance the representation and inference capabilities of clinical decision support systems.

## Additional file

Additional file 1: List of SWRL rules. (DOCX 26 kb)

## Abbreviations

ADA: American Diabetes Association
BFO: Basic Formal Ontology
CDSS: Clinical Decision Support System
CPG: Clinical Practice Guideline
CQ: Competency Question
DDO: Diabetes Diagnosis Ontology
DMTO: Diabetes Mellitus Treatment Ontology
EASD: European Association for the Study of Diabetes
EHR: Electronic Health Record
NCBO: National Center for Biomedical Ontology
NDF RT: National Drug Format Reference Terminology
OBO: Open Biomedical Ontologies
OGMS: Ontology for General Medical Science
ORSD: Ontology Requirements Specification Document
OWL: Web Ontology Language
PATO: Phenotypic Quality Ontology
PCR: Plan Checking Rule
PDR: Patient Diagnosis Rule
PER: Patient Evaluation Rule
PTR: Patient Treatment Rule
SCT: SNOMED CT
SNOMED CT: Systematized Nomenclature of Medicine—Clinical Terms
SQWRL: Semantic Query-Enhanced Web Rule Language
SWRL: Semantic Web Rule Language
T1DM: Type 1 diabetes mellitus
T2DM: Type 2 diabetes mellitus
TP: Treatment Plan

## Footnotes

1
*http://ifomis.uni-saarland.de/bfo/*

2
*https://bioportal.bioontology.org/ontologies/OGMS*

3
*https://bioportal.bioontology.org* */*

4
*http://www.obofoundry.org* */*

5
*https://bioportal.bioontology.org/ontologies*

6
*https://bioportal.bioontology.org/ontologies/DIAB*

7
*https://www.nlm.nih.gov/research/umls/rxnorm/*

8
*https://www.nlm.nih.gov/research/umls/sourcereleasedocs/current/NDFRT/*

9
*https://bioportal.bioontology.org/ontologies/OF*

10
*https://* *www.w3.org/TR/2016/WD-owl-time-20160712/*

11
*https://bioportal.bioontology.org/ontologies/SMASHPHYSICAL*

12
*http://oops.linkeddata.es* */*
