# Supplementary material for: DMTO: a realistic ontology for standard diabetes mellitus treatment
Source: J Biomed Semantics. 2018 Feb 6;9:8. doi: 10.1186/s13326-018-0176-y (PMC5800094; doi:10.1186/s13326-018-0176-y)
Supplement: Additional file 1: — List of SWRL rules. (DOCX 26 kb) [file 13326_2018_176_MOESM1_ESM.docx]

**Additional file 1:** The list of SWRL rules implemented in the DMTO ontology.

1. FPG(?f), 'impaired fasting glycaemia'(?t2), 'millimoles per liter'(?mm), 'patient profile'(?r), 'symptomatic patient'(?p), has_lab_test(?r, ?f), has_measurement_unit(?f, ?mu), has_patient_profile(?p, ?r), has_quantitative_Value(?f, ?q), swrlb:greaterThanOrEqual(?q, "6.1"^^xsd:double), swrlb:lessThanOrEqual(?q, "6.9"^^xsd:double) -> has_diagnosis(?r, ?t2)
2. acetazolamide(?n), patient(?p), 'patient profile'(?f), has_patient_profile(?p, ?f), currently_taken_drug(?f, ?n) -> biguanide_contradictor(?p)
3. nephropathy(?n), patient(?p), 'patient profile'(?f), has_patient_profile(?p, ?f), has_complication(?f, ?n) -> biguanide_contradictor(?p)
4. meal(?bf), meal(?din), meal(?lu), meal(?sn1), meal(?sn2), patient(?x), 'treatment plan'(?tp), 'patient profile'(?y), diet(?di), 'lifestyle subplan'(?sub), has_patient_profile(?x, ?y), has_treatment_plan(?y, ?tp), has_part(?tp, ?sub), has_lifestyle_participant(?sub, ?di), has_breakfast_meal(?di, ?bf), has_lunch_meal(?di, ?lu), has_dinner_meal(?di, ?din), has_snack_meal_1(?di, ?sn1), has_snack_meal_2(?di, ?sn2), has_total_calories(?y, ?ca), swrlb:divide(?carbs_grams_bf, ?amount_carbs_bf, 4), swrlb:divide(?carbs_grams_din, ?amount_carbs_din, 4), swrlb:divide(?carbs_grams_lu, ?amount_carbs_lu, 4), swrlb:divide(?carbs_grams_sn1, ?amount_carbs_sn1, 4), swrlb:divide(?carbs_grams_sn2, ?amount_carbs_sn2, 4), swrlb:divide(?fat_grams_bf, ?amount_fat_bf, 9), swrlb:divide(?fat_grams_din, ?amount_fat_din, 9), swrlb:divide(?fat_grams_lu, ?amount_fat_lu, 9), swrlb:divide(?fat_grams_sn1, ?amount_fat_sn1, 9), swrlb:divide(?fat_grams_sn2, ?amount_fat_sn2, 9), swrlb:divide(?prot_grams_bf, ?amount_prot_bf, 4), swrlb:divide(?prot_grams_din, ?amount_prot_din, 4), swrlb:divide(?prot_grams_lu, ?amount_prot_lu, 4), swrlb:divide(?prot_grams_sn1, ?amount_prot_sn1, 4), swrlb:divide(?prot_grams_sn2, ?amount_prot_sn2, 4), swrlb:multiply(?amount_bf, ?ca, "0.25"^^xsd:double), swrlb:multiply(?amount_carbs_bf, ?amount_bf, "0.5"^^xsd:double), swrlb:multiply(?amount_carbs_din, ?amount_din, "0.5"^^xsd:double), swrlb:multiply(?amount_carbs_lu, ?amount_lu, "0.5"^^xsd:double), swrlb:multiply(?amount_carbs_sn1, ?amount_sn1, "0.5"^^xsd:double), swrlb:multiply(?amount_carbs_sn2, ?amount_sn2, "0.5"^^xsd:double), swrlb:multiply(?amount_din, ?ca, "0.25"^^xsd:double), swrlb:multiply(?amount_fat_bf, ?amount_bf, "0.3"^^xsd:double), swrlb:multiply(?amount_fat_din, ?amount_din, "0.3"^^xsd:double), swrlb:multiply(?amount_fat_ln, ?amount_ln, "0.3"^^xsd:double), swrlb:multiply(?amount_fat_sn1, ?amount_sn1, "0.3"^^xsd:double), swrlb:multiply(?amount_fat_sn2, ?amount_sn2, "0.3"^^xsd:double), swrlb:multiply(?amount_lu, ?ca, "0.25"^^xsd:double), swrlb:multiply(?amount_prot_bf, ?amount_bf, "0.2"^^xsd:double), swrlb:multiply(?amount_prot_din, ?amount_din, "0.2"^^xsd:double), swrlb:multiply(?amount_prot_ln, ?amount_ln, "0.2"^^xsd:double), swrlb:multiply(?amount_prot_sn1, ?amount_sn1, "0.2"^^xsd:double), swrlb:multiply(?amount_prot_sn2, ?amount_sn2, "0.2"^^xsd:double), swrlb:multiply(?amount_sn1, ?ca, "0.125"^^xsd:double) -> has_carbohydrate_per_meal(?bf, ?amount_carbs_bf), has_carbohydrate_per_meal(?din, ?amount_carbs_din), has_carbohydrate_per_meal(?lu, ?amount_carbs_lu), has_carbohydrate_per_meal(?sn1, ?amount_carbs_sn1), has_carbohydrate_per_meal(?sn2, ?amount_carbs_sn2), has_amount_of_calorie_for_meal(?bf, ?amount_bf), has_amount_of_calorie_for_meal(?din, ?amount_din), has_amount_of_calorie_for_meal(?lu, ?amount_lu), has_amount_of_calorie_for_meal(?sn1, ?amount_sn1), has_amount_of_calorie_for_meal(?sn2, ?amount_sn2), has_fat_per_meal(?bf, ?amount_fat_bf), has_fat_per_meal(?din, ?amount_fat_din), has_fat_per_meal(?lu, ?amount_fat_lu), has_fat_per_meal(?sn1, ?amount_fat_sn1), has_fat_per_meal(?sn2, ?amount_fat_sn2), has_protein_per_meal(?bf, ?amount_prot_bf), has_protein_per_meal(?din, ?amount_prot_din), has_protein_per_meal(?lu, ?amount_prot_lu), has_protein_per_meal(?sn1, ?amount_prot_sn1), has_protein_per_meal(?sn2, ?amount_prot_sn2), has_carbohydrate_grams(?bf, ?carbs_grams_bf), has_carbohydrate_grams(?din, ?carbs_grams_din), has_carbohydrate_grams(?ln, ?carbs_grams_ln), has_carbohydrate_grams(?sn1, ?carbs_grams_sn1), has_carbohydrate_grams(?sn2, ?carbs_grams_sn2), has_fat_grams(?bf, ?fat_grams_bf), has_fat_grams(?din, ?fat_grams_din), has_fat_grams(?ln, ?fat_grams_ln), has_fat_grams(?sn1, ?fat_grams_sn1), has_fat_grams(?sn2, ?fat_grams_sn2), has_protein_grams(?bf, ?prot_grams_bf), has_protein_grams(?din, ?prot_grams_din), has_protein_grams(?ln, ?prot_grams_ln), has_protein_grams(?sn1, ?prot_grams_sn1), has_protein_grams(?sn2, ?prot_grams_sn2)
5. nephropathy(?n), patient(?p), 'patient profile'(?f), has_patient_profile(?p, ?f), has_complication(?f, ?n) -> incretin_contradictor(?p)
6. 'choline salicylate product'(?n), patient(?p), 'patient profile'(?f), has_patient_profile(?p, ?f), currently_taken_drug(?f, ?n) -> sulfonylurea_contradictor(?p)
7. benazepril(?n), patient(?p), 'patient profile'(?f), has_patient_profile(?p, ?f), currently_taken_drug(?f, ?n) -> biguanide_contradictor(?p)
8. patient(?p), 'patient profile'(?f), dofetilide(?n), has_patient_profile(?p, ?f), currently_taken_drug(?f, ?n) -> biguanide_contradictor(?p)
9. nephropathy(?n), patient(?p), 'patient profile'(?f), has_patient_profile(?p, ?f), has_complication(?f, ?n) -> sulfonylurea_contradictor(?p)
10. darunavir(?n), patient(?p), 'patient profile'(?f), has_patient_profile(?p, ?f), currently_taken_drug(?f, ?n) -> biguanide_contradictor(?p)
11. 'kilogram per square meter'(?kg), age(?a), BMI(?bmi), 'physically inactive'(?ph), 'first-degree relative with diabetes'(?fi), HbA1c(?hb), hypertension(?hyper), 'polycystic ovaries'(?po), 'cardiovascular disease'(?card), percent(?mm), 'gestational diabetes mellitus'(?gis), patient(?x), 'patient profile'(?y), 'high risk ethnicity'(?hir), has_demographic(?y, ?a), has_demographic(?y, ?hir), has_physical_examination(?y, ?fi), has_physical_examination(?y, ?ph), has_lab_test(?y, ?bmi), has_lab_test(?y, ?hb), has_measurement_unit(?bmi, ?kg), has_measurement_unit(?hb, ?mu), has_patient_profile(?x, ?y), has_complication(?y, ?card), has_complication(?y, ?gis), has_complication(?y, ?hyper), has_complication(?y, ?po), has_quantitative_Value(?a, ?v), has_quantitative_Value(?bmi, ?q5), has_quantitative_Value(?hb, ?q1), swrlb:greaterThanOrEqual(?q1, "5.7"^^xsd:double), swrlb:greaterThanOrEqual(?q5, 25), swrlb:greaterThanOrEqual(?v, 45) -> 'symptomatic patient'(?x)
12. age(?a), patient(?x), 'patient profile'(?y), has_demographic(?y, ?a), has_patient_profile(?x, ?y), has_quantitative_Value(?a, ?v), swrlb:lessThanOrEqual(?v, 10) -> child(?x)
13. obesity(?n), patient(?p), 'patient profile'(?f), has_patient_profile(?p, ?f), has_complication(?f, ?n) -> thiazolidinedione_contradictor(?p)
14. clozapine(?n), patient(?p), 'patient profile'(?f), has_patient_profile(?p, ?f), currently_taken_drug(?f, ?n) -> biguanide_contradictor(?p)
15. age(?a), 'foot disease'(?z1), patient(?x), 'patient profile'(?y), has_patient_profile(?x, ?y), has_complication(?y, ?z1), has_quantitative_Value(?a, ?v), swrlb:greaterThanOrEqual(?v, 45) -> 'symptomatic patient'(?x)
16. patient(?p), 'treatment plan'(?tp), 'patient profile'(?pp), 'drug subplan'(?dsup), has_patient_profile(?p, ?pp), has_part(?tp, ?dsup), has_target_achieved(?tp, true), has_drug_subplan_level(?dsup, "monotherapy"), has_passed_3_months(?tp, true) -> has_message(?tp, "Continue on the same monotherapy plan for another 3 months.")
17. patient(?x), 'treatment plan'(?tp), 'patient profile'(?y), 'diabetes mellitus'(?z1), 'physical activity'(?pa), 'lifestyle subplan'(?lp), 'aerobic exercise'(?ae), child(?x), 'days per week'(?dpw), 'minutes per day'(?aeu), has_diagnosis(?y, ?z1), has_patient_profile(?x, ?y), has_part(?tp, ?lp), has_aerobic_exercise(?pa, ?ae), has_lifestyle_participant(?lp, ?lfs) -> has_exercise_duration_UoM(?ae, ?aeu), has_exercise_frequency_UoM(?ae, ?dpw), has_exercise_duration(?ae, 60), has_intensity_percent(?ae, 50), has_exercise_frequency(?ae, 3)
18. anemia(?z1), patient(?x), 'patient profile'(?y), has_patient_profile(?x, ?y), has_complication(?y, ?z1) -> 'misleaded with HbA1c'(?x)
19. patient(?p), 'respiratory failure'(?n), 'patient profile'(?f), has_patient_profile(?p, ?f), has_complication(?f, ?n) -> biguanide_contradictor(?p)
20. 'Duration description'(?dd), 'Time instant'(?i1), 'Time instant'(?i2), 'Proper interval'(?i), patient(?p), 'treatment plan'(?tp), tablet(?t), metformin(?m), exenatide(?sul), 'drug timing'('while meal'), target(?tar), 'patient profile'(?pp), 'drug subplan'(?dsp), sulfonylurea_contradictor(?p), thiazolidinedione_contradictor(?p), SGLTI_contradictor(?p), 'DPP-4 inhibitor contradictor'(?p), 'not GLP-1 contradictor'(?p), oral(?or), 'has beginning'(?i, ?i1), 'has duration description'(?i, ?dd), 'has end'(?i, ?i2), has_patient_profile(?p, ?pp), has_treatment_plan(?pp, ?tp), has_part(?tp, ?dsp), has_target(?tp, ?tar), has_drug_participant(?dsp, ?m), months(?dd, 3), has_A1C_level(?tar, "6.5"^^xsd:double), has_previous_treatment_plan(?p, true), has_target_achieved(?tp, false), has_drug_subplan_level(?dsup, "monotherapy"), can_buy_expensive_drug(?p, true), has_passed_3_months(?tp, true), DifferentFrom (?i1, ?i2) -> has_doseForm(?sul, ?t), has_route_of_administration(?sul, ?or), when_to_take(?sul, 'while meal'), has_duration_backup(?tar, ?i), has_drug_participant(?dsp, ?sul), has_dose(?sul, 10), has_previous_treatment_plan(?p, true), has_drug_subplan_level(?dsp, "dual therapy")
21. 'Duration description'(?dd), 'Time instant'(?i1), 'Time instant'(?i2), 'Proper interval'(?i), patient(?p), 'treatment plan'(?tp), tablet(?t), sulfonylurea(?sul), metformin(?m), canagliflozin(?tzd), 'drug timing'('while meal'), target(?tar), 'patient profile'(?pp), 'drug subplan'(?dsp), thiazolidinedione_contradictor(?p), 'DPP-4 inhibitor contradictor'(?p), not_SGLTI_contradictor(?p), oral(?or), 'has beginning'(?i, ?i1), 'has duration description'(?i, ?dd), 'has end'(?i, ?i2), has_patient_profile(?p, ?pp), has_treatment_plan(?pp, ?tp), has_part(?tp, ?dsp), has_target(?tp, ?tar), has_drug_participant(?dsp, ?m), has_drug_participant(?dsp, ?sul), months(?dd, 3), has_A1C_level(?tar, "6.5"^^xsd:double), has_previous_treatment_plan(?p, true), has_target_achieved(?tp, false), has_drug_subplan_level(?dsup, "dual therapy"), has_passed_3_months(?tp, true), DifferentFrom (?i1, ?i2) -> has_doseForm(?tzd, ?t), has_route_of_administration(?tzd, ?or), when_to_take(?tzd, 'while meal'), has_duration_backup(?tar, ?i), has_drug_participant(?dsp, ?tzd), has_dose(?tzd, 300), has_previous_treatment_plan(?p, true), has_drug_subplan_level(?dsp, "triple therapy")
22. patient(?x), 'patient profile'(?y), Sedentary(?a), has_demographic(?y, ?a), has_patient_profile(?x, ?y), has_basal_metabolic_rate(?y, ?b), swrlb:multiply(?last, ?b, "1.2"^^xsd:double) -> has_total_calories(?y, ?last)
23. phenelzine(?n), patient(?p), 'patient profile'(?f), has_patient_profile(?p, ?f), currently_taken_drug(?f, ?n) -> sulfonylurea_contradictor(?p)
24. age(?a), 'disorder of adrenal gland'(?z1), patient(?x), 'patient profile'(?y), has_patient_profile(?x, ?y), has_complication(?y, ?z1), has_quantitative_Value(?a, ?v), swrlb:greaterThanOrEqual(?v, 45) -> 'symptomatic patient'(?x)
25. hyponatremia(?n), patient(?p), 'patient profile'(?f), has_patient_profile(?p, ?f), has_complication(?f, ?n) -> sulfonylurea_contradictor(?p)
26. FPG(?f), FPG(?f2), 'type 2 diabetes mellitus'(?t2), 'millimoles per liter'(?mm), 'patient profile'(?r), 'asymptomatic patient'(?p), has_lab_test(?r, ?f), has_lab_test(?r, ?f2), has_measurement_unit(?f, ?mu), has_measurement_unit(?f2, ?mu), has_patient_profile(?p, ?r), has_quantitative_Value(?f, ?q), has_quantitative_Value(?f2, ?q2), swrlb:greaterThanOrEqual(?q, "7.0"^^xsd:double), swrlb:greaterThanOrEqual(?q2, "7.0"^^xsd:double), DifferentFrom (?f, ?f2) -> has_diagnosis(?r, ?t2)
27. 'Duration description'(?dd), 'Time instant'(?i1), 'Time instant'(?i2), 'Proper interval'(?i), patient(?p), 'treatment plan'(?tp), tablet(?t), metformin(?m), Glipizide(?sul), 'drug timing'('while meal'), target(?tar), 'patient profile'(?pp), 'drug subplan'(?dsp), not_sulfonylurea_contradictor(?p), oral(?or), 'has beginning'(?i, ?i1), 'has duration description'(?i, ?dd), 'has end'(?i, ?i2), has_patient_profile(?p, ?pp), has_treatment_plan(?pp, ?tp), has_part(?tp, ?dsp), has_target(?tp, ?tar), has_drug_participant(?dsp, ?m), months(?dd, 3), has_A1C_level(?tar, "6.5"^^xsd:double), has_previous_treatment_plan(?p, true), has_target_achieved(?tp, false), has_drug_subplan_level(?dsup, "monotherapy"), can_buy_low_price_drug(?p, true), has_passed_3_months(?tp, true), DifferentFrom (?i1, ?i2) -> has_doseForm(?sul, ?t), has_route_of_administration(?sul, ?or), when_to_take(?sul, 'while meal'), has_duration_backup(?tar, ?i), has_drug_participant(?dsp, ?sul), has_dose(?sul, 10), has_previous_treatment_plan(?p, true), has_drug_subplan_level(?dsp, "dual therapy")
28. 'Duration description'(?dd), 'Time instant'(?i1), 'Time instant'(?i2), 'Proper interval'(?i), patient(?p), 'treatment plan'(?tp), tablet(?t), metformin(?m), 'dipeptidyl peptidase 4 (DPP-4) inhibitor'(?sul), pioglitazone(?tzd), 'drug timing'('while meal'), target(?tar), 'patient profile'(?pp), 'drug subplan'(?dsp), sulfonylurea_contradictor(?p), not_thiazolidinedione_contradictor(?p), oral(?or), 'has beginning'(?i, ?i1), 'has duration description'(?i, ?dd), 'has end'(?i, ?i2), has_patient_profile(?p, ?pp), has_treatment_plan(?pp, ?tp), has_part(?tp, ?dsp), has_target(?tp, ?tar), has_drug_participant(?dsp, ?m), has_drug_participant(?dsp, ?sul), months(?dd, 3), has_A1C_level(?tar, "6.5"^^xsd:double), has_previous_treatment_plan(?p, true), has_target_achieved(?tp, false), has_drug_subplan_level(?dsup, "dual therapy"), has_passed_3_months(?tp, true), DifferentFrom (?i1, ?i2) -> has_doseForm(?tzd, ?t), has_route_of_administration(?tzd, ?or), when_to_take(?tzd, 'while meal'), has_duration_backup(?tar, ?i), has_drug_participant(?dsp, ?tzd), has_dose(?tzd, 45), has_previous_treatment_plan(?p, true), has_drug_subplan_level(?dsp, "triple therapy")
29. 'kilogram per square meter'(?m), BMI(?a), patient(?x), 'patient profile'(?y), 'obese class III'(?u), has_physical_examination(?y, ?a), has_measurement_unit(?a, ?m), has_patient_profile(?x, ?y), has_quantitative_Value(?a, ?v), swrlb:greaterThanOrEqual(?v, "40.0"^^xsd:double) -> has_demographic(?y, ?u)
30. age(?a), 'acanthosis nigricans'(?z1), patient(?x), 'patient profile'(?y), has_demographic(?y, ?a), has_patient_profile(?x, ?y), has_complication(?y, ?z1), has_quantitative_Value(?a, ?v), swrlb:greaterThanOrEqual(?v, 45) -> 'symptomatic patient'(?x)
31. asenapine(?n), patient(?p), 'patient profile'(?f), has_patient_profile(?p, ?f), currently_taken_drug(?f, ?n) -> biguanide_contradictor(?p)
32. 'Duration description'(?dd), 'Time instant'(?i1), 'Time instant'(?i2), 'Proper interval'(?i), patient(?p), 'treatment plan'(?tp), tablet(?t), sulfonylurea(?sul), metformin(?m), exenatide(?tzd), 'drug timing'('while meal'), target(?tar), 'patient profile'(?pp), 'drug subplan'(?dsp), thiazolidinedione_contradictor(?p), SGLTI_contradictor(?p), 'DPP-4 inhibitor contradictor'(?p), 'not GLP-1 contradictor'(?p), oral(?or), 'has beginning'(?i, ?i1), 'has duration description'(?i, ?dd), 'has end'(?i, ?i2), has_patient_profile(?p, ?pp), has_treatment_plan(?pp, ?tp), has_part(?tp, ?dsp), has_target(?tp, ?tar), has_drug_participant(?dsp, ?m), has_drug_participant(?dsp, ?sul), months(?dd, 3), has_A1C_level(?tar, "6.5"^^xsd:double), has_previous_treatment_plan(?p, true), has_target_achieved(?tp, false), has_drug_subplan_level(?dsup, "dual therapy"), has_passed_3_months(?tp, true), DifferentFrom (?i1, ?i2) -> has_doseForm(?tzd, ?t), has_route_of_administration(?tzd, ?or), when_to_take(?tzd, 'while meal'), has_duration_backup(?tar, ?i), has_drug_participant(?dsp, ?tzd), has_dose(?tzd, 10), has_previous_treatment_plan(?p, true), has_drug_subplan_level(?dsp, "triple therapy")
33. HbA1c(?f), 'type 2 diabetes mellitus'(?t2), percent(?mm), 'patient profile'(?r), 'symptomatic patient'(?p), has_lab_test(?r, ?f), has_measurement_unit(?f, ?mu), has_patient_profile(?p, ?r), has_quantitative_Value(?f, ?q), swrlb:greaterThanOrEqual(?q, "6.5"^^xsd:double) -> has_diagnosis(?r, ?t2)
34. HbA1c(?f1), HbA1c(?f2), 'type 2 diabetes mellitus'(?t2), percent(?mm), 'patient profile'(?r), 'asymptomatic patient'(?p), 'suitable for HbA1c test'(?p), has_lab_test(?r, ?f1), has_lab_test(?r, ?f2), has_measurement_unit(?f1, ?mu), has_measurement_unit(?f2, ?mu), has_patient_profile(?p, ?r), has_quantitative_Value(?f1, ?q1), has_quantitative_Value(?f2, ?q2), swrlb:greaterThanOrEqual(?q1, "6.5"^^xsd:double), swrlb:greaterThanOrEqual(?q2, "6.5"^^xsd:double), DifferentFrom (?f1, ?f2) -> has_diagnosis(?r, ?t2)
35. FPG(?f), OGTT(?f2), 'normal patient'(?t2), 'patient profile'(?r), 'symptomatic patient'(?p), 'milligrams per deciliter'(?mm), has_lab_test(?r, ?f), has_lab_test(?r, ?f2), has_measurement_unit(?f, ?mu), has_measurement_unit(?f2, ?mu), has_patient_profile(?p, ?r), has_quantitative_Value(?f, ?q), has_quantitative_Value(?f2, ?q2), swrlb:lessThanOrEqual(?q, 100), swrlb:lessThanOrEqual(?q2, 140) -> has_diagnosis(?r, ?t2)
36. age(?a), gender(?gen), 'polycystic ovaries'(?z1), patient(?x), 'patient profile'(?y), has_demographic(?y, ?a), has_demographic(?y, ?gen), has_patient_profile(?x, ?y), has_complication(?y, ?z1), has_quantitative_Value(?a, ?v), has_qualitative_value(?gen, "female"^^xsd:string), swrlb:greaterThanOrEqual(?v, 45) -> 'symptomatic patient'(?x)
37. pancreatitis(?n), patient(?p), 'patient profile'(?f), has_patient_profile(?p, ?f), has_complication(?f, ?n) -> incretin_contradictor(?p)
38. age(?a), 'impaired glucose tolerance'(?z1), patient(?x), 'patient profile'(?y), has_demographic(?y, ?a), has_patient_profile(?x, ?y), has_complication(?y, ?z1), has_quantitative_Value(?a, ?v), swrlb:greaterThanOrEqual(?v, 45) -> 'symptomatic patient'(?x)
39. patient(?p), 'treatment plan'(?tp), 'patient profile'(?pp), 'drug subplan'(?dsup), has_patient_profile(?p, ?pp), has_part(?tp, ?dsup), has_target_achieved(?tp, true), has_drug_subplan_level(?dsup, "triple therapy"), has_passed_3_months(?tp, true) -> has_message(?tp, "Continue on the same triple therapy plan for another 3 months.")
40. 'Duration description'(?dd), 'Time instant'(?i1), 'Time instant'(?i2), 'Proper interval'(?i), patient(?p), 'treatment plan'(?tp), tablet(?t), metformin(?m), 'Sodium-Glucose Transporter 2 (SGLT-2) Inhibitor'(?sul), Glyburide(?tzd), 'drug timing'('while meal'), target(?tar), 'patient profile'(?pp), 'drug subplan'(?dsp), not_sulfonylurea_contradictor(?p), oral(?or), 'has beginning'(?i, ?i1), 'has duration description'(?i, ?dd), 'has end'(?i, ?i2), has_patient_profile(?p, ?pp), has_treatment_plan(?pp, ?tp), has_part(?tp, ?dsp), has_target(?tp, ?tar), has_drug_participant(?dsp, ?m), has_drug_participant(?dsp, ?sul), months(?dd, 3), has_A1C_level(?tar, "6.5"^^xsd:double), has_previous_treatment_plan(?p, true), has_target_achieved(?tp, false), has_drug_subplan_level(?dsup, "dual therapy"), has_passed_3_months(?tp, true), DifferentFrom (?i1, ?i2) -> has_doseForm(?tzd, ?t), has_route_of_administration(?tzd, ?or), when_to_take(?tzd, 'while meal'), has_duration_backup(?tar, ?i), has_drug_participant(?dsp, ?tzd), has_dose(?tzd, 5), has_previous_treatment_plan(?p, true), has_drug_subplan_level(?dsp, "triple therapy")
41. 'Duration description'(?dd), 'Time instant'(?i1), 'Time instant'(?i2), 'Proper interval'(?i), patient(?p), 'treatment plan'(?tp), tablet(?t), metformin(?m), 'dipeptidyl peptidase 4 (DPP-4) inhibitor'(?sul), canagliflozin(?tzd), 'drug timing'('while meal'), target(?tar), 'patient profile'(?pp), 'drug subplan'(?dsp), sulfonylurea_contradictor(?p), thiazolidinedione_contradictor(?p), not_SGLTI_contradictor(?p), oral(?or), 'has beginning'(?i, ?i1), 'has duration description'(?i, ?dd), 'has end'(?i, ?i2), has_patient_profile(?p, ?pp), has_treatment_plan(?pp, ?tp), has_part(?tp, ?dsp), has_target(?tp, ?tar), has_drug_participant(?dsp, ?m), has_drug_participant(?dsp, ?sul), months(?dd, 3), has_A1C_level(?tar, "6.5"^^xsd:double), has_previous_treatment_plan(?p, true), has_target_achieved(?tp, false), has_drug_subplan_level(?dsup, "dual therapy"), has_passed_3_months(?tp, true), DifferentFrom (?i1, ?i2) -> has_doseForm(?tzd, ?t), has_route_of_administration(?tzd, ?or), when_to_take(?tzd, 'while meal'), has_duration_backup(?tar, ?i), has_drug_participant(?dsp, ?tzd), has_dose(?tzd, 300), has_previous_treatment_plan(?p, true), has_drug_subplan_level(?dsp, "triple therapy")
42. patient(?p), dulaglutide(?n), 'patient profile'(?f), has_patient_profile(?p, ?f), currently_taken_drug(?f, ?n) -> biguanide_contradictor(?p)
43. rifampin(?n), patient(?p), 'patient profile'(?f), has_patient_profile(?p, ?f), currently_taken_drug(?f, ?n) -> sulfonylurea_contradictor(?p)
44. age(?a), patient(?x), 'family history'(?f), 'patient profile'(?y), has_demographic(?y, ?a), has_patient_profile(?x, ?y), has_history(?y, ?f), has_quantitative_Value(?a, ?v), number_of_first_degree_relatives_with_type2_diabetes(?f, ?n), swrlb:greaterThanOrEqual(?n, 1), swrlb:greaterThanOrEqual(?v, 45) -> 'symptomatic patient'(?x)
45. 'Duration description'(?dd), 'Time instant'(?i1), 'Time instant'(?i2), 'Proper interval'(?i), patient(?p), 'treatment plan'(?tp), tablet(?t), dapagliflozin(?m), target(?tar), 'patient profile'(?pp), 'diabetes mellitus'(?dm), 'drug subplan'(?dsp), 'asymptomatic patient'(?p), biguanide_contradictor(?p), sulfonylurea_contradictor(?p), thiazolidinedione_contradictor(?p), 'DPP-4 inhibitor contradictor'(?p), not_SGLTI_contradictor(?p), oral(?or), has_diagnosis(?pp, ?dm), 'has beginning'(?i, ?i1), 'has duration description'(?i, ?dd), 'has end'(?i, ?i2), has_patient_profile(?p, ?pp), has_treatment_plan(?pp, ?tp), has_part(?tp, ?dsp), has_target(?tp, ?tar), months(?dd, 3), has_A1C_level(?tar, "6.5"^^xsd:double), has_previous_treatment_plan(?p, false), has_acceptance_level(?m, "accepted"), can_buy_expensive_drug(?p, true), DifferentFrom (?i1, ?i2) -> has_doseForm(?m, ?t), has_route_of_administration(?m, ?or), has_duration_backup(?tar, ?i), has_drug_participant(?dsp, ?m), has_dose(?m, 10), has_previous_treatment_plan(?p, true), has_drug_subplan_level(?dsp, "monotherapy")
46. age(?a), obesity(?z1), patient(?x), 'patient profile'(?y), has_demographic(?y, ?a), has_patient_profile(?x, ?y), has_complication(?y, ?z1), has_quantitative_Value(?a, ?v), swrlb:greaterThanOrEqual(?v, 45) -> 'symptomatic patient'(?x)
47. patient(?p), 'patient profile'(?f), dolutegravir(?n), has_patient_profile(?p, ?f), currently_taken_drug(?f, ?n) -> biguanide_contradictor(?p)
48. age(?a), hypertension(?z1), patient(?x), 'patient profile'(?y), has_demographic(?y, ?a), has_patient_profile(?x, ?y), has_complication(?y, ?z1), has_quantitative_Value(?a, ?v), swrlb:greaterThanOrEqual(?v, 45) -> 'symptomatic patient'(?x)
49. patient(?x), 'treatment plan'(?tp), 'patient profile'(?y), 'diabetes mellitus'(?z1), 'physical activity'(?pa), 'lifestyle subplan'(?lp), 'aerobic exercise'(?ae), teenager(?x), 'days per week'(?dpw), 'minutes per day'(?aeu), has_diagnosis(?y, ?z1), has_patient_profile(?x, ?y), has_part(?tp, ?lp), has_aerobic_exercise(?pa, ?ae), has_lifestyle_participant(?lp, ?lfs) -> has_exercise_duration_UoM(?ae, ?aeu), has_exercise_frequency_UoM(?ae, ?dpw), has_exercise_duration(?ae, 60), has_intensity_percent(?ae, 50), has_exercise_frequency(?ae, 3)
50. 'Duration description'(?dd), 'Time instant'(?i1), 'Time instant'(?i2), 'Proper interval'(?i), patient(?p), 'treatment plan'(?tp), tablet(?t), metformin(?m), linagliptin(?sul), 'drug timing'('while meal'), target(?tar), 'patient profile'(?pp), 'drug subplan'(?dsp), sulfonylurea_contradictor(?p), thiazolidinedione_contradictor(?p), 'not DPP-4 inhibitor contradictor'(?p), oral(?or), 'has beginning'(?i, ?i1), 'has duration description'(?i, ?dd), 'has end'(?i, ?i2), has_patient_profile(?p, ?pp), has_treatment_plan(?pp, ?tp), has_part(?tp, ?dsp), has_target(?tp, ?tar), has_drug_participant(?dsp, ?m), months(?dd, 3), has_A1C_level(?tar, "6.5"^^xsd:double), has_previous_treatment_plan(?p, true), has_target_achieved(?tp, false), has_drug_subplan_level(?dsup, "monotherapy"), can_buy_expensive_drug(?p, true), has_passed_3_months(?tp, true), DifferentFrom (?i1, ?i2) -> has_doseForm(?sul, ?t), has_route_of_administration(?sul, ?or), when_to_take(?sul, 'while meal'), has_duration_backup(?tar, ?i), has_drug_participant(?dsp, ?sul), has_dose(?sul, 5), has_previous_treatment_plan(?p, true), has_drug_subplan_level(?dsp, "dual therapy")
51. patient(?p), has_social_state(?p, "poor"^^xsd:string) -> can_buy_low_price_drug(?p, true)
52. age(?a), dyslipidemia(?z1), patient(?x), 'patient profile'(?y), has_patient_profile(?x, ?y), has_complication(?y, ?z1), has_quantitative_Value(?a, ?v), swrlb:greaterThanOrEqual(?v, 45) -> 'symptomatic patient'(?x)
53. age(?a), gender(?gg), height(?h), weight(?w), patient(?x), 'patient profile'(?y), has_demographic(?y, ?a), has_demographic(?y, ?gg), has_demographic(?y, ?h), has_demographic(?y, ?w), has_patient_profile(?x, ?y), has_quantitative_Value(?a, ?a1), has_quantitative_Value(?h, ?h1), has_quantitative_Value(?w, ?w1), has_qualitative_value(?gg, "male"^^xsd:string), swrlb:add(?v11, "66.47"^^xsd:double, ?v1, ?v2), swrlb:multiply(?v1, "13.75"^^xsd:double, ?w1), swrlb:multiply(?v2, "5.0"^^xsd:double, ?h1), swrlb:multiply(?v3, "6.75"^^xsd:double, ?a1), swrlb:subtract(?vlast, ?v11, ?v3) -> has_basal_metabolic_rate(?y, ?vlast)
54. patient(?p), 'patient profile'(?f), fleroxacin(?n), has_patient_profile(?p, ?f), currently_taken_drug(?f, ?n) -> biguanide_contradictor(?p)
55. tranylcypromine(?n), patient(?p), 'patient profile'(?f), has_patient_profile(?p, ?f), currently_taken_drug(?f, ?n) -> sulfonylurea_contradictor(?p)
56. age(?a), 'cardiovascular disease'(?z1), patient(?x), 'patient profile'(?y), has_demographic(?y, ?a), has_patient_profile(?x, ?y), has_complication(?y, ?z1), has_quantitative_Value(?a, ?v), swrlb:greaterThanOrEqual(?v, 45) -> 'symptomatic patient'(?x)
57. HbA1c(?f1), HbA1c(?f2), 'type 2 diabetes mellitus'(?t2), percent(?mm), 'patient profile'(?r), 'asymptomatic patient'(?p), 'suitable for HbA1c test'(?p), has_lab_test(?r, ?f1), has_lab_test(?r, ?f2), has_measurement_unit(?f1, ?mu), has_measurement_unit(?f2, ?mu), has_patient_profile(?p, ?r), has_quantitative_Value(?f1, ?q1), has_quantitative_Value(?f2, ?q2), swrlb:greaterThanOrEqual(?q1, "6.5"^^xsd:double), swrlb:greaterThanOrEqual(?q2, "5.6"^^xsd:double), DifferentFrom (?f1, ?f2) -> has_diagnosis(?r, ?t2)
58. 'Duration description'(?dd), 'Time instant'(?i1), 'Time instant'(?i2), 'Proper interval'(?i), patient(?p), 'treatment plan'(?tp), tablet(?t), sulfonylurea(?sul), metformin(?m), sitagliptin(?tzd), 'drug timing'('while meal'), target(?tar), 'patient profile'(?pp), 'drug subplan'(?dsp), thiazolidinedione_contradictor(?p), 'not DPP-4 inhibitor contradictor'(?p), oral(?or), 'has beginning'(?i, ?i1), 'has duration description'(?i, ?dd), 'has end'(?i, ?i2), has_patient_profile(?p, ?pp), has_treatment_plan(?pp, ?tp), has_part(?tp, ?dsp), has_target(?tp, ?tar), has_drug_participant(?dsp, ?m), has_drug_participant(?dsp, ?sul), months(?dd, 3), has_A1C_level(?tar, "6.5"^^xsd:double), has_previous_treatment_plan(?p, true), has_target_achieved(?tp, false), has_drug_subplan_level(?dsup, "dual therapy"), has_passed_3_months(?tp, true), DifferentFrom (?i1, ?i2) -> has_doseForm(?tzd, ?t), has_route_of_administration(?tzd, ?or), when_to_take(?tzd, 'while meal'), has_duration_backup(?tar, ?i), has_drug_participant(?dsp, ?tzd), has_dose(?tzd, 100), has_previous_treatment_plan(?p, true), has_drug_subplan_level(?dsp, "triple therapy")
59. patient(?p), 'vitamin B12 deficiency'(?n), 'patient profile'(?f), has_patient_profile(?p, ?f), has_complication(?f, ?n) -> biguanide_contradictor(?p)
60. food(?f), drug(?dd), patient(?x), 'patient profile'(?y), contradicte_with_food(?dd, ?f), has_patient_profile(?x, ?y), currently_taken_drug(?y, ?dd) -> has_forbidden_food(?y, ?f)
61. indinavir(?n), patient(?p), 'patient profile'(?f), has_patient_profile(?p, ?f), currently_taken_drug(?f, ?n) -> biguanide_contradictor(?p)
62. nephropathy(?z1), patient(?x), 'patient profile'(?y), has_patient_profile(?x, ?y), has_complication(?y, ?z1) -> 'misleaded with HbA1c'(?x)
63. patient(?p), liraglutide(?n), 'patient profile'(?f), has_patient_profile(?p, ?f), currently_taken_drug(?f, ?n) -> biguanide_contradictor(?p)
64. patient(?x), 'patient profile'(?y), 'moderately active'(?a), has_demographic(?y, ?a), has_patient_profile(?x, ?y), has_basal_metabolic_rate(?y, ?b), swrlb:multiply(?last, ?b, "1.55"^^xsd:double) -> has_total_calories(?y, ?last)
65. isocarboxazid(?n), patient(?p), 'patient profile'(?f), has_patient_profile(?p, ?f), currently_taken_drug(?f, ?n) -> sulfonylurea_contradictor(?p)
66. patient(?p), 'patient profile'(?f), gemifloxacin(?n), has_patient_profile(?p, ?f), currently_taken_drug(?f, ?n) -> biguanide_contradictor(?p)
67. gender(?g), patient(?p), 'breast feeding'(?pr), 'patient profile'(?f), has_demographic(?f, ?g), has_demographic(?f, ?pr), has_patient_profile(?p, ?f), has_qualitative_value(?g, "female"), has_qualitative_value(?pr, "breast feed") -> thiazolidinedione_contradictor(?p)
68. coma(?n), patient(?p), 'patient profile'(?f), has_patient_profile(?p, ?f), has_complication(?f, ?n) -> biguanide_contradictor(?p)
69. anemia(?n), patient(?p), 'patient profile'(?f), has_patient_profile(?p, ?f), has_complication(?f, ?n) -> sulfonylurea_contradictor(?p)
70. aspirin(?n), patient(?p), 'patient profile'(?f), has_patient_profile(?p, ?f), currently_taken_drug(?f, ?n) -> sulfonylurea_contradictor(?p)
71. 'heart disease'(?n), patient(?p), 'patient profile'(?f), has_patient_profile(?p, ?f), has_complication(?f, ?n) -> DA_contradictor(?p)
72. 'acute myocardial infarction'(?n), patient(?p), 'patient profile'(?f), has_patient_profile(?p, ?f), has_complication(?f, ?n) -> biguanide_contradictor(?p)
73. 'cardiovascular disease'(?n), patient(?p), 'patient profile'(?f), has_patient_profile(?p, ?f), has_complication(?f, ?n) -> sulfonylurea_contradictor(?p)
74. 'Duration description'(?dd), 'Time instant'(?i1), 'Time instant'(?i2), 'Proper interval'(?i), patient(?p), 'treatment plan'(?tp), tablet(?t), canagliflozin(?m), target(?tar), 'patient profile'(?pp), 'diabetes mellitus'(?dm), 'drug subplan'(?dsp), 'asymptomatic patient'(?p), biguanide_contradictor(?p), sulfonylurea_contradictor(?p), thiazolidinedione_contradictor(?p), 'DPP-4 inhibitor contradictor'(?p), not_SGLTI_contradictor(?p), oral(?or), has_diagnosis(?pp, ?dm), 'has beginning'(?i, ?i1), 'has duration description'(?i, ?dd), 'has end'(?i, ?i2), has_patient_profile(?p, ?pp), has_treatment_plan(?pp, ?tp), has_part(?tp, ?dsp), has_target(?tp, ?tar), months(?dd, 3), has_A1C_level(?tar, "6.5"^^xsd:double), has_previous_treatment_plan(?p, false), has_social_state(?pp, "rich"), has_acceptance_level(?m, "accepted"), can_buy_expensive_drug(?p, true), DifferentFrom (?i1, ?i2) -> has_doseForm(?m, ?t), has_route_of_administration(?m, ?or), has_duration_backup(?tar, ?i), has_drug_participant(?dsp, ?m), has_dose(?m, 300), has_previous_treatment_plan(?p, true), has_drug_subplan_level(?dsp, "monotherapy")
75. 'cardiovascular disease'(?n), patient(?p), 'patient profile'(?f), has_patient_profile(?p, ?f), has_complication(?f, ?n) -> meglitinide_contradictor(?p)
76. age(?a), 'metabolic syndrome X'(?z1), patient(?x), 'patient profile'(?y), has_demographic(?y, ?a), has_patient_profile(?x, ?y), has_complication(?y, ?z1), has_quantitative_Value(?a, ?v), swrlb:greaterThanOrEqual(?v, 45) -> 'symptomatic patient'(?x)
77. patient(?x), 'treatment plan'(?tp), 'patient profile'(?y), 'diabetes mellitus'(?z1), 'physical activity'(?pa), 'lifestyle subplan'(?lp), 'aerobic exercise'(?ae), adult(?x), 'days per week'(?dpw), 'minutes per day'(?aeu), has_diagnosis(?y, ?z1), has_patient_profile(?x, ?y), has_part(?tp, ?lp), has_aerobic_exercise(?pa, ?ae), has_lifestyle_participant(?lp, ?lfs) -> has_exercise_duration_UoM(?ae, ?aeu), has_exercise_frequency_UoM(?ae, ?dpw), has_exercise_duration(?ae, 150), has_intensity_percent(?ae, 50), has_exercise_frequency(?ae, 3)
78. age(?a), steroid(?z1), patient(?x), 'patient profile'(?y), has_demographic(?y, ?a), has_patient_profile(?x, ?y), currently_taken_drug(?y, ?z1), has_quantitative_Value(?a, ?v), swrlb:greaterThanOrEqual(?v, 45) -> 'symptomatic patient'(?x)
79. gender(?g), patient(?p), 'breast feeding'(?pr), 'patient profile'(?f), has_demographic(?f, ?g), has_demographic(?f, ?pr), has_patient_profile(?p, ?f), has_qualitative_value(?g, "female"), has_qualitative_value(?pr, "breast feed") -> 'GLP-1 contradictor'(?p)
80. anemia(?n), patient(?p), 'patient profile'(?f), has_patient_profile(?p, ?f), has_complication(?f, ?n) -> thiazolidinedione_contradictor(?p)
81. gender(?g), patient(?p), 'breast feeding'(?pr), 'patient profile'(?f), has_demographic(?f, ?g), has_demographic(?f, ?pr), has_patient_profile(?p, ?f), has_qualitative_value(?g, "female"), has_qualitative_value(?pr, "breast feed") -> 'DPP-4 inhibitor contradictor'(?p)
82. age(?a), 'coronary arteriosclerosis'(?z1), patient(?x), 'patient profile'(?y), has_demographic(?y, ?a), has_patient_profile(?x, ?y), has_complication(?y, ?z1), has_quantitative_Value(?a, ?v), swrlb:greaterThanOrEqual(?v, 45) -> 'symptomatic patient'(?x)
83. 'Duration description'(?dd), 'Time instant'(?i1), 'Time instant'(?i2), 'Proper interval'(?i), patient(?p), 'treatment plan'(?tp), tablet(?t), sulfonylurea(?sul), metformin(?m), pioglitazone(?tzd), 'drug timing'('while meal'), target(?tar), 'patient profile'(?pp), 'drug subplan'(?dsp), not_thiazolidinedione_contradictor(?p), oral(?or), 'has beginning'(?i, ?i1), 'has duration description'(?i, ?dd), 'has end'(?i, ?i2), has_patient_profile(?p, ?pp), has_treatment_plan(?pp, ?tp), has_part(?tp, ?dsp), has_target(?tp, ?tar), has_drug_participant(?dsp, ?m), has_drug_participant(?dsp, ?sul), months(?dd, 3), has_A1C_level(?tar, "6.5"^^xsd:double), has_previous_treatment_plan(?p, true), has_target_achieved(?tp, false), has_drug_subplan_level(?dsup, "dual therapy"), has_passed_3_months(?tp, true), DifferentFrom (?i1, ?i2) -> has_doseForm(?tzd, ?t), has_route_of_administration(?tzd, ?or), when_to_take(?tzd, 'while meal'), has_duration_backup(?tar, ?i), has_drug_participant(?dsp, ?tzd), has_dose(?tzd, 45), has_previous_treatment_plan(?p, true), has_drug_subplan_level(?dsp, "triple therapy")
84. FPG(?f), 'type 2 diabetes mellitus'(?t2), 'millimoles per liter'(?mm), 'patient profile'(?r), 'symptomatic patient'(?p), has_lab_test(?r, ?f), has_measurement_unit(?f, ?mu), has_patient_profile(?p, ?r), has_quantitative_Value(?f, ?q), swrlb:greaterThanOrEqual(?q, "7.0"^^xsd:double) -> has_diagnosis(?r, ?t2)
85. 'impaired fasting glycaemia'(?z1), patient(?x), 'treatment plan'(?tp), 'patient profile'(?y), 'physical activity'(?pa), 'lifestyle subplan'(?lp), 'aerobic exercise'(?ae), child(?x), 'days per week'(?dpw), 'minutes per day'(?aeu), has_diagnosis(?y, ?z1), has_patient_profile(?x, ?y), has_part(?tp, ?lp), has_aerobic_exercise(?pa, ?ae), has_lifestyle_participant(?lp, ?lfs) -> has_exercise_duration_UoM(?ae, ?aeu), has_exercise_frequency_UoM(?ae, ?dpw), has_exercise_duration(?ae, 60), has_intensity_percent(?ae, 50), has_exercise_frequency(?ae, 3)
86. 'Duration description'(?dd), 'Time instant'(?i1), 'Time instant'(?i2), 'Proper interval'(?i), patient(?p), 'treatment plan'(?tp), tablet(?t), pioglitazone(?m), target(?tar), 'patient profile'(?pp), 'diabetes mellitus'(?dm), 'drug subplan'(?dsp), 'asymptomatic patient'(?p), biguanide_contradictor(?p), sulfonylurea_contradictor(?p), not_thiazolidinedione_contradictor(?p), oral(?or), has_diagnosis(?pp, ?dm), 'has beginning'(?i, ?i1), 'has duration description'(?i, ?dd), 'has end'(?i, ?i2), has_patient_profile(?p, ?pp), has_treatment_plan(?pp, ?tp), has_part(?tp, ?dsp), has_target(?tp, ?tar), months(?dd, 3), has_A1C_level(?tar, "6.5"^^xsd:double), has_previous_treatment_plan(?p, false), has_acceptance_level(?m, "accepted"), can_buy_low_price_drug(?p, true), DifferentFrom (?i1, ?i2) -> has_doseForm(?m, ?t), has_route_of_administration(?m, ?or), has_duration_backup(?tar, ?i), has_drug_participant(?dsp, ?m), has_dose(?m, 45), has_previous_treatment_plan(?p, true), has_drug_subplan_level(?dsp, "monotherapy")
87. 'kilogram per square meter'(?m), BMI(?a), patient(?x), 'patient profile'(?y), 'normal weight'(?u), has_physical_examination(?y, ?a), has_measurement_unit(?a, ?m), has_patient_profile(?x, ?y), has_quantitative_Value(?a, ?v), swrlb:greaterThanOrEqual(?v, "18.5"^^xsd:double), swrlb:lessThanOrEqual(?v, "24.9"^^xsd:double) -> has_demographic(?y, ?u)
88. patient(?p), 'patient profile'(?f), porphyria(?n), has_patient_profile(?p, ?f), has_complication(?f, ?n) -> sulfonylurea_contradictor(?p)
89. 'Duration description'(?dd), 'Time instant'(?i1), 'Time instant'(?i2), 'Proper interval'(?i), patient(?p), 'treatment plan'(?tp), tablet(?t), metformin(?m), 'dipeptidyl peptidase 4 (DPP-4) inhibitor'(?sul), Glyburide(?tzd), 'drug timing'('while meal'), target(?tar), 'patient profile'(?pp), 'drug subplan'(?dsp), not_sulfonylurea_contradictor(?p), oral(?or), 'has beginning'(?i, ?i1), 'has duration description'(?i, ?dd), 'has end'(?i, ?i2), has_patient_profile(?p, ?pp), has_treatment_plan(?pp, ?tp), has_part(?tp, ?dsp), has_target(?tp, ?tar), has_drug_participant(?dsp, ?m), has_drug_participant(?dsp, ?sul), months(?dd, 3), has_A1C_level(?tar, "6.5"^^xsd:double), has_previous_treatment_plan(?p, true), has_target_achieved(?tp, false), has_drug_subplan_level(?dsup, "dual therapy"), has_passed_3_months(?tp, true), DifferentFrom (?i1, ?i2) -> has_doseForm(?tzd, ?t), has_route_of_administration(?tzd, ?or), when_to_take(?tzd, 'while meal'), has_duration_backup(?tar, ?i), has_drug_participant(?dsp, ?tzd), has_dose(?tzd, 5), has_previous_treatment_plan(?p, true), has_drug_subplan_level(?dsp, "triple therapy")
90. patient(?p), has_social_state(?p, "rich"^^xsd:string) -> can_buy_intermediate_drug(?p, true)
91. patient(?p), has_social_state(?p, "rich"^^xsd:string) -> can_buy_low_price_drug(?p, true)
92. patient(?p), has_social_state(?p, "rich"^^xsd:string) -> can_buy_expensive_drug(?p, true)
93. patient(?p), albiglutide(?n), 'patient profile'(?f), has_patient_profile(?p, ?f), currently_taken_drug(?f, ?n) -> biguanide_contradictor(?p)
94. patient(?p), 'treatment plan'(?tp), 'patient profile'(?pp), 'drug subplan'(?dsup), has_patient_profile(?p, ?pp), has_part(?tp, ?dsup), has_target_achieved(?tp, true), has_drug_subplan_level(?dsup, "dual therapy"), has_passed_3_months(?tp, true) -> has_message(?tp, "Continue on the same dual therapy plan for another 3 months.")
95. 'diabetic ketoacidosis'(?n), patient(?p), 'patient profile'(?f), has_patient_profile(?p, ?f), has_complication(?f, ?n) -> AGI_contradictor(?p)
96. 'heart failure'(?n), patient(?p), 'patient profile'(?f), has_patient_profile(?p, ?f), has_complication(?f, ?n) -> thiazolidinedione_contradictor(?p)
97. FPG(?f2), RPG(?f), 'type 2 diabetes mellitus'(?t2), 'millimoles per liter'(?mm), 'patient profile'(?r), 'asymptomatic patient'(?p), has_lab_test(?r, ?f), has_lab_test(?r, ?f2), has_measurement_unit(?f, ?mu), has_measurement_unit(?f2, ?mu), has_patient_profile(?p, ?r), has_quantitative_Value(?f, ?q), has_quantitative_Value(?f2, ?q2), swrlb:greaterThanOrEqual(?q, "7.0"^^xsd:double), swrlb:greaterThanOrEqual(?q2, "11.1"^^xsd:double), DifferentFrom (?f, ?f2) -> has_diagnosis(?r, ?t2)
98. age(?a), 'thiazide diuretic'(?z1), 'beta-Blocking agent'(?z3), patient(?x), 'patient profile'(?y), has_demographic(?y, ?a), has_patient_profile(?x, ?y), currently_taken_drug(?y, ?z1), currently_taken_drug(?y, ?z3), has_quantitative_Value(?a, ?v), swrlb:greaterThanOrEqual(?v, 45) -> 'symptomatic patient'(?x)
99. 'heart failure'(?n), patient(?p), 'patient profile'(?f), has_patient_profile(?p, ?f), has_complication(?f, ?n) -> biguanide_contradictor(?p)
100. 'Duration description'(?dd), 'Time instant'(?i1), 'Time instant'(?i2), 'Proper interval'(?i), patient(?p), 'treatment plan'(?tp), tablet(?t), metformin(?m), 'Sodium-Glucose Transporter 2 (SGLT-2) Inhibitor'(?sul), pioglitazone(?tzd), 'drug timing'('while meal'), target(?tar), 'patient profile'(?pp), 'drug subplan'(?dsp), sulfonylurea_contradictor(?p), not_thiazolidinedione_contradictor(?p), oral(?or), 'has beginning'(?i, ?i1), 'has duration description'(?i, ?dd), 'has end'(?i, ?i2), has_patient_profile(?p, ?pp), has_treatment_plan(?pp, ?tp), has_part(?tp, ?dsp), has_target(?tp, ?tar), has_drug_participant(?dsp, ?m), has_drug_participant(?dsp, ?sul), months(?dd, 3), has_A1C_level(?tar, "6.5"^^xsd:double), has_previous_treatment_plan(?p, true), has_target_achieved(?tp, false), has_drug_subplan_level(?dsup, "dual therapy"), has_passed_3_months(?tp, true), DifferentFrom (?i1, ?i2) -> has_doseForm(?tzd, ?t), has_route_of_administration(?tzd, ?or), when_to_take(?tzd, 'while meal'), has_duration_backup(?tar, ?i), has_drug_participant(?dsp, ?tzd), has_dose(?tzd, 45), has_previous_treatment_plan(?p, true), has_drug_subplan_level(?dsp, "triple therapy")
101. age(?a), 'peripheral arterial disease (PAD)'(?z1), patient(?x), 'patient profile'(?y), has_demographic(?y, ?a), has_patient_profile(?x, ?y), has_complication(?y, ?z1), has_quantitative_Value(?a, ?v), swrlb:greaterThanOrEqual(?v, 45) -> 'symptomatic patient'(?x)
102. 'disorder of intestine'(?n), patient(?p), 'patient profile'(?f), has_patient_profile(?p, ?f), has_complication(?f, ?n) -> AGI_contradictor(?p)
103. 'hepatic cirrhosis'(?n), patient(?p), 'patient profile'(?f), has_patient_profile(?p, ?f), has_complication(?f, ?n) -> AGI_contradictor(?p)
104. 'Duration description'(?dd), 'Time instant'(?i1), 'Time instant'(?i2), 'Proper interval'(?i), patient(?p), 'treatment plan'(?tp), tablet(?t), thiazolidinedione(?sul), metformin(?m), exenatide(?tzd), 'drug timing'('while meal'), target(?tar), 'patient profile'(?pp), 'drug subplan'(?dsp), sulfonylurea_contradictor(?p), SGLTI_contradictor(?p), 'DPP-4 inhibitor contradictor'(?p), 'not GLP-1 contradictor'(?p), oral(?or), 'has beginning'(?i, ?i1), 'has duration description'(?i, ?dd), 'has end'(?i, ?i2), has_patient_profile(?p, ?pp), has_treatment_plan(?pp, ?tp), has_part(?tp, ?dsp), has_target(?tp, ?tar), has_drug_participant(?dsp, ?m), has_drug_participant(?dsp, ?sul), months(?dd, 3), has_A1C_level(?tar, "6.5"^^xsd:double), has_previous_treatment_plan(?p, true), has_target_achieved(?tp, false), has_drug_subplan_level(?dsup, "dual therapy"), has_passed_3_months(?tp, true), DifferentFrom (?i1, ?i2) -> has_doseForm(?tzd, ?t), has_route_of_administration(?tzd, ?or), when_to_take(?tzd, 'while meal'), has_duration_backup(?tar, ?i), has_drug_participant(?dsp, ?tzd), has_dose(?tzd, 10), has_previous_treatment_plan(?p, true), has_drug_subplan_level(?dsp, "triple therapy")
105. 'Duration description'(?dd), 'Time instant'(?i1), 'Time instant'(?i2), 'Proper interval'(?i), patient(?p), 'treatment plan'(?tp), tablet(?t), albiglutide(?m), target(?tar), 'patient profile'(?pp), 'diabetes mellitus'(?dm), 'drug subplan'(?dsp), 'asymptomatic patient'(?p), biguanide_contradictor(?p), sulfonylurea_contradictor(?p), thiazolidinedione_contradictor(?p), SGLTI_contradictor(?p), 'DPP-4 inhibitor contradictor'(?p), 'not GLP-1 contradictor'(?p), oral(?or), has_diagnosis(?pp, ?dm), 'has beginning'(?i, ?i1), 'has duration description'(?i, ?dd), 'has end'(?i, ?i2), has_patient_profile(?p, ?pp), has_treatment_plan(?pp, ?tp), has_part(?tp, ?dsp), has_target(?tp, ?tar), months(?dd, 3), has_A1C_level(?tar, "6.5"^^xsd:double), has_previous_treatment_plan(?p, false), has_acceptance_level(?m, "accepted"), can_buy_expensive_drug(?p, true), DifferentFrom (?i1, ?i2) -> has_doseForm(?m, ?t), has_route_of_administration(?m, ?or), has_duration_backup(?tar, ?i), has_drug_participant(?dsp, ?m), has_dose(?m, 50), has_previous_treatment_plan(?p, true), has_drug_subplan_level(?dsp, "monotherapy")
106. hypotension(?n), patient(?p), 'patient profile'(?f), has_patient_profile(?p, ?f), has_complication(?f, ?n) -> DA_contradictor(?p)
107. 'milligram per liter'(?mg), HbA1c(?f1), FPG(?f2), 'type 2 diabetes mellitus'(?t2), percent(?mm), 'patient profile'(?r), 'asymptomatic patient'(?p), has_lab_test(?r, ?f1), has_lab_test(?r, ?f2), has_measurement_unit(?f1, ?mu), has_measurement_unit(?f2, ?mg), has_patient_profile(?p, ?r), has_quantitative_Value(?f1, ?q1), has_quantitative_Value(?f2, ?q2), swrlb:greaterThanOrEqual(?q1, "6.5"^^xsd:double), swrlb:greaterThanOrEqual(?q2, "7.0"^^xsd:double) -> has_diagnosis(?r, ?t2)
108. 'impaired fasting glycaemia'(?z1), patient(?x), 'treatment plan'(?tp), 'patient profile'(?y), 'physical activity'(?pa), 'lifestyle subplan'(?lp), 'aerobic exercise'(?ae), teenager(?x), 'days per week'(?dpw), 'minutes per day'(?aeu), has_diagnosis(?y, ?z1), has_patient_profile(?x, ?y), has_part(?tp, ?lp), has_aerobic_exercise(?pa, ?ae), has_lifestyle_participant(?lp, ?lfs) -> has_exercise_duration_UoM(?ae, ?aeu), has_exercise_frequency_UoM(?ae, ?dpw), has_exercise_duration(?ae, 60), has_intensity_percent(?ae, 50), has_exercise_frequency(?ae, 3)
109. 'gestational diabetes mellitus'(?z1), patient(?x), 'patient profile'(?y), has_patient_profile(?x, ?y), has_complication(?y, ?z1) -> 'symptomatic patient'(?x)
110. OGTT(?f2), 'millimoles per liter'(?mu), 'gestational diabetes mellitus'(?zz), 'patient profile'(?r), 'symptomatic patient'(?x), has_lab_test(?r, ?f2), has_measurement_unit(?f2, ?mu), has_patient_profile(?x, ?r), has_quantitative_Value(?f2, ?q1), swrlb:greaterThanOrEqual(?q1, "8.5"^^xsd:double) -> has_diagnosis(?r, ?zz)
111. 'kilogram per square meter'(?kg), age(?a), BMI(?bmi), 'anti-psychotic agent'(?z2), 'corticoid preparation'(?z1), 'gestational diabetes mellitus'(?gis), patient(?x), 'patient profile'(?y), 'high risk ethnicity'(?hir), has_demographic(?y, ?a), has_demographic(?y, ?hir), has_lab_test(?y, ?bmi), has_measurement_unit(?bmi, ?kg), has_patient_profile(?x, ?y), has_complication(?y, ?gis), currently_taken_drug(?y, ?z1), currently_taken_drug(?y, ?z2), has_quantitative_Value(?a, ?v), has_quantitative_Value(?bmi, ?q5), swrlb:greaterThanOrEqual(?q5, 30), swrlb:greaterThanOrEqual(?v, 40) -> 'symptomatic patient'(?x)
112. FPG(?f1), OGTT(?f2), 'type 2 diabetes mellitus'(?t2), 'millimoles per liter'(?mm), 'patient profile'(?r), 'asymptomatic patient'(?p), has_lab_test(?r, ?f1), has_lab_test(?r, ?f2), has_measurement_unit(?f1, ?mu), has_measurement_unit(?f2, ?mu), has_patient_profile(?p, ?r), has_quantitative_Value(?f1, ?q1), has_quantitative_Value(?f2, ?q2), swrlb:greaterThanOrEqual(?q1, "6.1"^^xsd:double), swrlb:greaterThanOrEqual(?q2, "11.1"^^xsd:double), swrlb:lessThanOrEqual(?q1, "6.9"^^xsd:double) -> has_diagnosis(?r, ?t2)
113. 'malignant tumor of urinary bladder'(?n), patient(?p), 'patient profile'(?f), has_patient_profile(?p, ?f), has_complication(?f, ?n) -> thiazolidinedione_contradictor(?p)
114. osteoporosis(?n), patient(?p), 'patient profile'(?f), has_patient_profile(?p, ?f), has_complication(?f, ?n) -> thiazolidinedione_contradictor(?p)
115. 'Duration description'(?dd), 'Time instant'(?i1), 'Time instant'(?i2), 'Proper interval'(?i), patient(?p), 'treatment plan'(?tp), tablet(?t), alogliptin(?m), target(?tar), 'patient profile'(?pp), 'diabetes mellitus'(?dm), 'drug subplan'(?dsp), 'asymptomatic patient'(?p), biguanide_contradictor(?p), sulfonylurea_contradictor(?p), thiazolidinedione_contradictor(?p), 'not DPP-4 inhibitor contradictor'(?p), oral(?or), has_diagnosis(?pp, ?dm), 'has beginning'(?i, ?i1), 'has duration description'(?i, ?dd), 'has end'(?i, ?i2), has_patient_profile(?p, ?pp), has_treatment_plan(?pp, ?tp), has_part(?tp, ?dsp), has_target(?tp, ?tar), months(?dd, 3), has_A1C_level(?tar, "6.5"^^xsd:double), has_previous_treatment_plan(?p, false), has_acceptance_level(?m, "accepted"), can_buy_expensive_drug(?p, true), DifferentFrom (?i1, ?i2) -> has_doseForm(?m, ?t), has_route_of_administration(?m, ?or), has_duration_backup(?tar, ?i), has_drug_participant(?dsp, ?m), has_dose(?m, 25), has_previous_treatment_plan(?p, true), has_drug_subplan_level(?dsp, "monotherapy")
116. 'milligram per liter'(?mg), HbA1c(?f1), RPG(?f2), 'type 2 diabetes mellitus'(?t2), percent(?mm), 'patient profile'(?r), 'asymptomatic patient'(?p), has_lab_test(?r, ?f1), has_lab_test(?r, ?f2), has_measurement_unit(?f1, ?mu), has_measurement_unit(?f2, ?mg), has_patient_profile(?p, ?r), has_quantitative_Value(?f1, ?q1), has_quantitative_Value(?f2, ?q2), swrlb:greaterThanOrEqual(?q1, "6.5"^^xsd:double), swrlb:greaterThanOrEqual(?q2, "11.1"^^xsd:double) -> has_diagnosis(?r, ?t2)
117. 'Duration description'(?dd), 'Time instant'(?i1), 'Time instant'(?i2), 'Proper interval'(?i), patient(?p), 'treatment plan'(?tp), tablet(?t), glimepiride(?m), target(?tar), 'patient profile'(?pp), 'diabetes mellitus'(?dm), 'drug subplan'(?dsp), biguanide_contradictor(?p), not_sulfonylurea_contradictor(?p), oral(?or), has_diagnosis(?pp, ?dm), 'has beginning'(?i, ?i1), 'has duration description'(?i, ?dd), 'has end'(?i, ?i2), has_patient_profile(?p, ?pp), has_treatment_plan(?pp, ?tp), has_part(?tp, ?dsp), has_target(?tp, ?tar), months(?dd, 3), has_A1C_level(?tar, "6.5"^^xsd:double), has_previous_treatment_plan(?p, false), can_buy_low_price_drug(?p, true), DifferentFrom (?i1, ?i2) -> has_doseForm(?m, ?t), has_route_of_administration(?m, ?or), when_to_take(?m, 'while meal'), has_drug_participant(?dsp, ?m), has_dose(?m, 4), has_previous_treatment_plan(?p, true), has_drug_subplan_level(?dsp, "monotherapy"), has_duration(?tar, "3.0"^^xsd:double)
118. hypoxia(?n), patient(?p), 'patient profile'(?f), has_patient_profile(?p, ?f), has_complication(?f, ?n) -> biguanide_contradictor(?p)
119. age(?a), patient(?x), 'patient profile'(?y), has_demographic(?y, ?a), has_patient_profile(?x, ?y), has_quantitative_Value(?a, ?v), swrlb:greaterThan(?v, 10), swrlb:lessThan(?v, 18) -> teenager(?x)
120. 'Duration description'(?dd), 'Time instant'(?i1), 'Time instant'(?i2), 'Proper interval'(?i), patient(?p), 'treatment plan'(?tp), tablet(?t), sitagliptin(?m), target(?tar), 'patient profile'(?pp), 'diabetes mellitus'(?dm), 'drug subplan'(?dsp), 'asymptomatic patient'(?p), biguanide_contradictor(?p), sulfonylurea_contradictor(?p), thiazolidinedione_contradictor(?p), 'not DPP-4 inhibitor contradictor'(?p), oral(?or), has_diagnosis(?pp, ?dm), 'has beginning'(?i, ?i1), 'has duration description'(?i, ?dd), 'has end'(?i, ?i2), has_patient_profile(?p, ?pp), has_treatment_plan(?pp, ?tp), has_part(?tp, ?dsp), has_target(?tp, ?tar), months(?dd, 3), has_A1C_level(?tar, "6.5"^^xsd:double), has_previous_treatment_plan(?p, false), has_acceptance_level(?m, "accepted"), can_buy_expensive_drug(?p, true), DifferentFrom (?i1, ?i2) -> has_doseForm(?m, ?t), has_route_of_administration(?m, ?or), has_duration_backup(?tar, ?i), has_drug_participant(?dsp, ?m), has_dose(?m, 100), has_previous_treatment_plan(?p, true), has_drug_subplan_level(?dsp, "monotherapy")
121. 'Duration description'(?dd), 'Time instant'(?i1), 'Time instant'(?i2), 'Proper interval'(?i), patient(?p), 'treatment plan'(?tp), tablet(?t), metformin(?m), pioglitazone(?sul), 'drug timing'('while meal'), target(?tar), 'patient profile'(?pp), 'drug subplan'(?dsp), sulfonylurea_contradictor(?p), not_thiazolidinedione_contradictor(?p), oral(?or), 'has beginning'(?i, ?i1), 'has duration description'(?i, ?dd), 'has end'(?i, ?i2), has_patient_profile(?p, ?pp), has_treatment_plan(?pp, ?tp), has_part(?tp, ?dsp), has_target(?tp, ?tar), has_drug_participant(?dsp, ?m), months(?dd, 3), has_A1C_level(?tar, "6.5"^^xsd:double), has_previous_treatment_plan(?p, true), has_target_achieved(?tp, false), has_drug_subplan_level(?dsup, "monotherapy"), can_buy_low_price_drug(?p, true), has_passed_3_months(?tp, true), DifferentFrom (?i1, ?i2) -> has_doseForm(?sul, ?t), has_route_of_administration(?sul, ?or), when_to_take(?sul, 'while meal'), has_duration_backup(?tar, ?i), has_drug_participant(?dsp, ?sul), has_dose(?sul, 45), has_previous_treatment_plan(?p, true), has_drug_subplan_level(?dsp, "dual therapy")
122. HbA1c(?a1c), patient(?p), 'treatment plan'(?tp), target(?tg), 'patient profile'(?pp), has_lab_test(?pp, ?a1c), has_patient_profile(?p, ?pp), has_treatment_plan(?pp, ?tp), has_target(?tp, ?tg), has_quantitative_Value(?a1c, ?v2), has_A1C_level(?tg, ?v1), has_date(?a1c, ?dat1), has_date(?tp, ?dat2), swrlb:greaterThan(?dat1, ?dat2), swrlb:greaterThanOrEqual(?v2, ?v1) -> has_target_achieved(?tp, false)
123. 'Duration description'(?dd), 'Time instant'(?i1), 'Time instant'(?i2), 'Proper interval'(?i), patient(?p), 'treatment plan'(?tp), tablet(?t), metformin(?m), Glyburide(?tzd), 'Glucagon-like peptide-1 receptor (GLP-1) analogue'(?sul), 'drug timing'('while meal'), target(?tar), 'patient profile'(?pp), 'drug subplan'(?dsp), not_sulfonylurea_contradictor(?p), oral(?or), 'has beginning'(?i, ?i1), 'has duration description'(?i, ?dd), 'has end'(?i, ?i2), has_patient_profile(?p, ?pp), has_treatment_plan(?pp, ?tp), has_part(?tp, ?dsp), has_target(?tp, ?tar), has_drug_participant(?dsp, ?m), has_drug_participant(?dsp, ?sul), months(?dd, 3), has_A1C_level(?tar, "6.5"^^xsd:double), has_previous_treatment_plan(?p, true), has_target_achieved(?tp, false), has_drug_subplan_level(?dsup, "dual therapy"), has_passed_3_months(?tp, true), DifferentFrom (?i1, ?i2) -> has_doseForm(?tzd, ?t), has_route_of_administration(?tzd, ?or), when_to_take(?tzd, 'while meal'), has_duration_backup(?tar, ?i), has_drug_participant(?dsp, ?tzd), has_dose(?tzd, 5), has_previous_treatment_plan(?p, true), has_drug_subplan_level(?dsp, "triple therapy")
124. diazoxide(?n), patient(?p), 'patient profile'(?f), has_patient_profile(?p, ?f), currently_taken_drug(?f, ?n) -> sulfonylurea_contradictor(?p)
125. 'Duration description'(?dd), 'Time instant'(?i1), 'Time instant'(?i2), 'Proper interval'(?i), patient(?p), 'treatment plan'(?tp), tablet(?t), metformin(?m), sitagliptin(?tzd), 'Sodium-Glucose Transporter 2 (SGLT-2) Inhibitor'(?sul), 'drug timing'('while meal'), target(?tar), 'patient profile'(?pp), 'drug subplan'(?dsp), sulfonylurea_contradictor(?p), thiazolidinedione_contradictor(?p), 'not DPP-4 inhibitor contradictor'(?p), oral(?or), 'has beginning'(?i, ?i1), 'has duration description'(?i, ?dd), 'has end'(?i, ?i2), has_patient_profile(?p, ?pp), has_treatment_plan(?pp, ?tp), has_part(?tp, ?dsp), has_target(?tp, ?tar), has_drug_participant(?dsp, ?m), has_drug_participant(?dsp, ?sul), months(?dd, 3), has_A1C_level(?tar, "6.5"^^xsd:double), has_previous_treatment_plan(?p, true), has_target_achieved(?tp, false), has_drug_subplan_level(?dsup, "dual therapy"), has_passed_3_months(?tp, true), DifferentFrom (?i1, ?i2) -> has_doseForm(?tzd, ?t), has_route_of_administration(?tzd, ?or), when_to_take(?tzd, 'while meal'), has_duration_backup(?tar, ?i), has_drug_participant(?dsp, ?tzd), has_dose(?tzd, 100), has_previous_treatment_plan(?p, true), has_drug_subplan_level(?dsp, "triple therapy")
126. HbA1c(?f1), HbA1c(?f2), 'impaired glucose tolerance'(?t2), percent(?mm), 'patient profile'(?r), 'asymptomatic patient'(?p), 'suitable for HbA1c test'(?p), has_lab_test(?r, ?f1), has_lab_test(?r, ?f2), has_measurement_unit(?f1, ?mu), has_measurement_unit(?f2, ?mu), has_patient_profile(?p, ?r), has_quantitative_Value(?f1, ?q1), has_quantitative_Value(?f2, ?q2), swrlb:greaterThanOrEqual(?q1, "6.5"^^xsd:double), swrlb:lessThanOrEqual(?q2, "5.6"^^xsd:double), DifferentFrom (?f1, ?f2) -> has_diagnosis(?r, ?t2)
127. 'Duration description'(?dd), 'Time instant'(?i1), 'Time instant'(?i2), 'Proper interval'(?i), patient(?p), 'treatment plan'(?tp), tablet(?t), metformin(?m), 'Sodium-Glucose Transporter 2 (SGLT-2) Inhibitor'(?sul), exenatide(?tzd), 'drug timing'('while meal'), target(?tar), 'patient profile'(?pp), 'drug subplan'(?dsp), sulfonylurea_contradictor(?p), thiazolidinedione_contradictor(?p), 'DPP-4 inhibitor contradictor'(?p), 'not GLP-1 contradictor'(?p), oral(?or), 'has beginning'(?i, ?i1), 'has duration description'(?i, ?dd), 'has end'(?i, ?i2), has_patient_profile(?p, ?pp), has_treatment_plan(?pp, ?tp), has_part(?tp, ?dsp), has_target(?tp, ?tar), has_drug_participant(?dsp, ?m), has_drug_participant(?dsp, ?sul), months(?dd, 3), has_A1C_level(?tar, "6.5"^^xsd:double), has_previous_treatment_plan(?p, true), has_target_achieved(?tp, false), has_drug_subplan_level(?dsup, "dual therapy"), has_passed_3_months(?tp, true), DifferentFrom (?i1, ?i2) -> has_doseForm(?tzd, ?t), has_route_of_administration(?tzd, ?or), when_to_take(?tzd, 'while meal'), has_duration_backup(?tar, ?i), has_drug_participant(?dsp, ?tzd), has_dose(?tzd, 10), has_previous_treatment_plan(?p, true), has_drug_subplan_level(?dsp, "triple therapy")
128. patient(?x), 'patient profile'(?y), 'extra active'(?a), has_demographic(?y, ?a), has_patient_profile(?x, ?y), has_basal_metabolic_rate(?y, ?b), swrlb:multiply(?last, ?b, "1.9"^^xsd:double) -> has_total_calories(?y, ?last)
129. 'hepatic failure'(?n), patient(?p), 'patient profile'(?f), has_patient_profile(?p, ?f), has_complication(?f, ?n) -> sulfonylurea_contradictor(?p)
130. 'hepatic failure'(?n), patient(?p), 'patient profile'(?f), has_patient_profile(?p, ?f), has_complication(?f, ?n) -> meglitinide_contradictor(?p)
131. 'kilogram per square meter'(?m), BMI(?a), patient(?x), 'patient profile'(?y), underweight(?u), has_physical_examination(?y, ?a), has_measurement_unit(?a, ?m), has_patient_profile(?x, ?y), has_quantitative_Value(?a, ?v), swrlb:lessThan(?v, "18.5"^^xsd:double) -> has_demographic(?y, ?u)
132. 'renal impairment'(?n), patient(?p), 'patient profile'(?f), has_patient_profile(?p, ?f), has_complication(?f, ?n) -> meglitinide_contradictor(?p)
133. 'hepatic failure'(?n), patient(?p), 'patient profile'(?f), has_patient_profile(?p, ?f), has_complication(?f, ?n) -> biguanide_contradictor(?p)
134. 'magnesium salicylate product'(?n), patient(?p), 'patient profile'(?f), has_patient_profile(?p, ?f), currently_taken_drug(?f, ?n) -> sulfonylurea_contradictor(?p)
135. 'hepatic failure'(?n), patient(?p), 'patient profile'(?f), has_patient_profile(?p, ?f), has_complication(?f, ?n) -> AGI_contradictor(?p)
136. patient(?p), hypoxemia(?n), 'patient profile'(?f), has_patient_profile(?p, ?f), has_complication(?f, ?n) -> biguanide_contradictor(?p)
137. patient(?p), 'patient profile'(?f), ethanol(?n), has_patient_profile(?p, ?f), currently_taken_drug(?f, ?n) -> biguanide_contradictor(?p)
138. 'kilogram per square meter'(?m), BMI(?a), patient(?x), 'patient profile'(?y), overweight(?u), has_physical_examination(?y, ?a), has_measurement_unit(?a, ?m), has_patient_profile(?x, ?y), has_quantitative_Value(?a, ?v), swrlb:greaterThanOrEqual(?v, "25.0"^^xsd:double), swrlb:lessThanOrEqual(?v, "29.9"^^xsd:double) -> has_demographic(?y, ?u)
139. age(?a), dyslipidemia(?dy), obesity(?z1), hypertension(?hi), 'acanthosis nigricans'(?z2), 'polycystic ovaries'(?po), patient(?x), 'patient profile'(?y), 'high risk ethnicity'(?hir), 'family history of gestational diabetes mellitus'(?fam), has_demographic(?y, ?a), has_demographic(?y, ?hir), has_physical_examination(?y, ?fam), has_patient_profile(?x, ?y), has_complication(?y, ?dy), has_complication(?y, ?hi), has_complication(?y, ?po), has_complication(?y, ?z1), has_complication(?y, ?z2), has_quantitative_Value(?a, ?v), swrlb:greaterThanOrEqual(?v, 10) -> 'symptomatic patient'(?x)
140. age(?a), gender(?gg), height(?h), weight(?w), patient(?x), 'patient profile'(?y), has_demographic(?y, ?a), has_demographic(?y, ?gg), has_demographic(?y, ?h), has_demographic(?y, ?w), has_patient_profile(?x, ?y), has_quantitative_Value(?a, ?a1), has_quantitative_Value(?h, ?h1), has_quantitative_Value(?w, ?w1), has_qualitative_value(?gg, "female"^^xsd:string), swrlb:add(?v11, "665.09"^^xsd:double, ?v1, ?v2), swrlb:multiply(?v1, "9.56"^^xsd:double, ?w1), swrlb:multiply(?v2, "1.84"^^xsd:double, ?h1), swrlb:multiply(?v3, "4.67"^^xsd:double, ?a1), swrlb:subtract(?vlast, ?v11, ?v3) -> has_basal_metabolic_rate(?y, ?vlast)
141. 'Duration description'(?dd), 'Time instant'(?i1), 'Time instant'(?i2), 'Proper interval'(?i), patient(?p), 'treatment plan'(?tp), tablet(?t), metformin(?m), pioglitazone(?tzd), 'Glucagon-like peptide-1 receptor (GLP-1) analogue'(?sul), 'drug timing'('while meal'), target(?tar), 'patient profile'(?pp), 'drug subplan'(?dsp), sulfonylurea_contradictor(?p), not_thiazolidinedione_contradictor(?p), oral(?or), 'has beginning'(?i, ?i1), 'has duration description'(?i, ?dd), 'has end'(?i, ?i2), has_patient_profile(?p, ?pp), has_treatment_plan(?pp, ?tp), has_part(?tp, ?dsp), has_target(?tp, ?tar), has_drug_participant(?dsp, ?m), has_drug_participant(?dsp, ?sul), months(?dd, 3), has_A1C_level(?tar, "6.5"^^xsd:double), has_previous_treatment_plan(?p, true), has_target_achieved(?tp, false), has_drug_subplan_level(?dsup, "dual therapy"), has_passed_3_months(?tp, true), DifferentFrom (?i1, ?i2) -> has_doseForm(?tzd, ?t), has_route_of_administration(?tzd, ?or), when_to_take(?tzd, 'while meal'), has_duration_backup(?tar, ?i), has_drug_participant(?dsp, ?tzd), has_dose(?tzd, 45), has_previous_treatment_plan(?p, true), has_drug_subplan_level(?dsp, "triple therapy")
142. 'Duration description'(?dd), 'Time instant'(?i1), 'Time instant'(?i2), 'Proper interval'(?i), patient(?p), 'treatment plan'(?tp), tablet(?t), metformin(?m), canagliflozin(?sul), 'drug timing'('while meal'), target(?tar), 'patient profile'(?pp), 'drug subplan'(?dsp), sulfonylurea_contradictor(?p), thiazolidinedione_contradictor(?p), 'DPP-4 inhibitor contradictor'(?p), not_SGLTI_contradictor(?p), oral(?or), 'has beginning'(?i, ?i1), 'has duration description'(?i, ?dd), 'has end'(?i, ?i2), has_patient_profile(?p, ?pp), has_treatment_plan(?pp, ?tp), has_part(?tp, ?dsp), has_target(?tp, ?tar), has_drug_participant(?dsp, ?m), months(?dd, 3), has_A1C_level(?tar, "6.5"^^xsd:double), has_previous_treatment_plan(?p, true), has_target_achieved(?tp, false), has_drug_subplan_level(?dsup, "monotherapy"), can_buy_expensive_drug(?p, true), has_passed_3_months(?tp, true), DifferentFrom (?i1, ?i2) -> has_doseForm(?sul, ?t), has_route_of_administration(?sul, ?or), when_to_take(?sul, 'while meal'), has_duration_backup(?tar, ?i), has_drug_participant(?dsp, ?sul), has_dose(?sul, 300), has_previous_treatment_plan(?p, true), has_drug_subplan_level(?dsp, "dual therapy")
143. patient(?p), has_social_state(?p, "intermediate"^^xsd:string) -> can_buy_low_price_drug(?p, true)
144. patient(?p), 'patient profile'(?f), amiodarone(?n), has_patient_profile(?p, ?f), currently_taken_drug(?f, ?n) -> biguanide_contradictor(?p)
145. HbA1c(?f2), RPG(?f), 'type 2 diabetes mellitus'(?t2), 'millimoles per liter'(?mm), percent(?per), 'patient profile'(?r), 'asymptomatic patient'(?p), has_lab_test(?r, ?f), has_lab_test(?r, ?f2), has_measurement_unit(?f, ?mu), has_measurement_unit(?f2, ?per), has_patient_profile(?p, ?r), has_quantitative_Value(?f, ?q), has_quantitative_Value(?f2, ?q2), swrlb:greaterThanOrEqual(?q, "11.1"^^xsd:double), swrlb:greaterThanOrEqual(?q2, "6.5"^^xsd:double), DifferentFrom (?f, ?f2) -> has_diagnosis(?r, ?t2)
146. 'Duration description'(?dd), 'Time instant'(?i1), 'Time instant'(?i2), 'Proper interval'(?i), patient(?p), 'treatment plan'(?tp), tablet(?t), Glyburide(?m), target(?tar), 'patient profile'(?pp), 'diabetes mellitus'(?dm), 'drug subplan'(?dsp), 'asymptomatic patient'(?p), biguanide_contradictor(?p), not_sulfonylurea_contradictor(?p), oral(?or), has_diagnosis(?pp, ?dm), 'has beginning'(?i, ?i1), 'has duration description'(?i, ?dd), 'has end'(?i, ?i2), has_patient_profile(?p, ?pp), has_treatment_plan(?pp, ?tp), has_part(?tp, ?dsp), has_target(?tp, ?tar), months(?dd, 3), has_A1C_level(?tar, "6.5"^^xsd:double), has_previous_treatment_plan(?p, false), has_acceptance_level(?m, "accepted"), can_buy_low_price_drug(?p, true), DifferentFrom (?i1, ?i2) -> has_doseForm(?m, ?t), has_route_of_administration(?m, ?or), when_to_take(?m, 'while meal'), has_duration_backup(?tar, ?i), has_drug_participant(?dsp, ?m), has_dose(?m, 5), has_previous_treatment_plan(?p, true), has_drug_subplan_level(?dsp, "monotherapy")
147. 'hepatic failure'(?n), patient(?p), 'patient profile'(?f), has_patient_profile(?p, ?f), has_complication(?f, ?n) -> thiazolidinedione_contradictor(?p)
148. HbA1c(?f1), OGTT(?f2), 'type 2 diabetes mellitus'(?t2), 'millimoles per liter'(?mm2), percent(?mm), 'patient profile'(?r), 'asymptomatic patient'(?p), 'suitable for HbA1c test'(?p), has_lab_test(?r, ?f1), has_lab_test(?r, ?f2), has_measurement_unit(?f1, ?mu), has_measurement_unit(?f2, ?mu2), has_patient_profile(?p, ?r), has_quantitative_Value(?f1, ?q1), has_quantitative_Value(?f2, ?q2), swrlb:greaterThanOrEqual(?q1, "6.5"^^xsd:double), swrlb:greaterThanOrEqual(?q2, "11.1"^^xsd:double) -> has_diagnosis(?r, ?t2)
149. patient(?p), has_social_state(?p, "intermediate"^^xsd:string) -> can_buy_intermediate_drug(?p, true)
150. 'Duration description'(?dd), 'Time instant'(?i1), 'Time instant'(?i2), 'Proper interval'(?i), patient(?p), 'treatment plan'(?tp), tablet(?t), metformin(?m), canagliflozin(?tzd), 'Glucagon-like peptide-1 receptor (GLP-1) analogue'(?sul), 'drug timing'('while meal'), target(?tar), 'patient profile'(?pp), 'drug subplan'(?dsp), sulfonylurea_contradictor(?p), thiazolidinedione_contradictor(?p), not_SGLTI_contradictor(?p), oral(?or), 'has beginning'(?i, ?i1), 'has duration description'(?i, ?dd), 'has end'(?i, ?i2), has_patient_profile(?p, ?pp), has_treatment_plan(?pp, ?tp), has_part(?tp, ?dsp), has_target(?tp, ?tar), has_drug_participant(?dsp, ?m), has_drug_participant(?dsp, ?sul), months(?dd, 3), has_A1C_level(?tar, "6.5"^^xsd:double), has_previous_treatment_plan(?p, true), has_target_achieved(?tp, false), has_drug_subplan_level(?dsup, "dual therapy"), has_passed_3_months(?tp, true), DifferentFrom (?i1, ?i2) -> has_doseForm(?tzd, ?t), has_route_of_administration(?tzd, ?or), when_to_take(?tzd, 'while meal'), has_duration_backup(?tar, ?i), has_drug_participant(?dsp, ?tzd), has_dose(?tzd, 300), has_previous_treatment_plan(?p, true), has_drug_subplan_level(?dsp, "triple therapy")
151. 'lactic acidosis'(?n), patient(?p), 'patient profile'(?f), has_patient_profile(?p, ?f), has_complication(?f, ?n) -> biguanide_contradictor(?p)
152. gender(?g), patient(?p), 'pregnancy state'(?pr), 'patient profile'(?f), has_demographic(?f, ?g), has_demographic(?f, ?pr), has_patient_profile(?p, ?f), has_qualitative_value(?g, "female"), has_qualitative_value(?pr, "pregnant") -> 'GLP-1 contradictor'(?p)
153. 'diabetes symptom'(?z1), 'diabetes symptom'(?z2), 'diabetes symptom'(?z3), 'diabetes symptom'(?z4), age(?a), patient(?x), 'patient profile'(?y), has_demographic(?y, ?a), has_patient_profile(?x, ?y), has_symptom(?y, ?z1), has_symptom(?y, ?z2), has_symptom(?y, ?z3), has_symptom(?y, ?z4), has_quantitative_Value(?a, ?v), swrlb:greaterThanOrEqual(?v, 45), swrlb:notEqual(?z1, ?z2), swrlb:notEqual(?z1, ?z3), swrlb:notEqual(?z1, ?z4), swrlb:notEqual(?z2, ?z3), swrlb:notEqual(?z2, ?z4), swrlb:notEqual(?z3, ?z4) -> 'symptomatic patient'(?x)
154. gender(?g), patient(?p), 'pregnancy state'(?pr), 'patient profile'(?f), has_demographic(?f, ?g), has_demographic(?f, ?pr), has_patient_profile(?p, ?f), has_qualitative_value(?g, "female"), has_qualitative_value(?pr, "pregnant") -> 'DPP-4 inhibitor contradictor'(?p)
155. patient(?p), 'Renal Dysfunction'(?n), 'patient profile'(?f), has_patient_profile(?p, ?f), has_complication(?f, ?n) -> incretin_contradictor(?p)
156. patient(?p), 'Renal Dysfunction'(?n), 'patient profile'(?f), has_patient_profile(?p, ?f), has_complication(?f, ?n) -> biguanide_contradictor(?p)
157. age(?a), 'cerebrovascular disease'(?z1), patient(?x), 'patient profile'(?y), has_demographic(?y, ?a), has_patient_profile(?x, ?y), has_complication(?y, ?z1), has_quantitative_Value(?a, ?v), swrlb:greaterThanOrEqual(?v, 45) -> 'symptomatic patient'(?x)
158. age(?a), patient(?x), 'patient profile'(?y), has_demographic(?y, ?a), has_patient_profile(?x, ?y), has_quantitative_Value(?a, ?v), swrlb:greaterThanOrEqual(?v, 18) -> adult(?x)
159. patient(?x), 'patient profile'(?y), 'very active'(?a), has_demographic(?y, ?a), has_patient_profile(?x, ?y), has_basal_metabolic_rate(?y, ?b), swrlb:multiply(?last, ?b, "1.725"^^xsd:double) -> has_total_calories(?y, ?last)
160. OGTT(?f), 'type 2 diabetes mellitus'(?t2), 'millimoles per liter'(?mm), 'patient profile'(?r), 'symptomatic patient'(?p), has_lab_test(?r, ?f), has_measurement_unit(?f, ?mu), has_patient_profile(?p, ?r), has_quantitative_Value(?f, ?q), swrlb:greaterThanOrEqual(?q, "11.1"^^xsd:double) -> has_diagnosis(?r, ?t2)
161. patient(?p), 'patient profile'(?f), colesevelam(?n), has_patient_profile(?p, ?f), currently_taken_drug(?f, ?n) -> biguanide_contradictor(?p)
162. depression(?n), patient(?p), 'patient profile'(?f), has_patient_profile(?p, ?f), has_complication(?f, ?n) -> DA_contradictor(?p)
163. chloramphenicol(?n), patient(?p), 'patient profile'(?f), has_patient_profile(?p, ?f), currently_taken_drug(?f, ?n) -> sulfonylurea_contradictor(?p)
164. patient(?p), 'Renal Dysfunction'(?n), 'patient profile'(?f), has_patient_profile(?p, ?f), has_complication(?f, ?n) -> AGI_contradictor(?p)
165. 'Duration description'(?dd), 'Time instant'(?i1), 'Time instant'(?i2), 'Proper interval'(?i), patient(?p), 'treatment plan'(?tp), tablet(?t), linagliptin(?m), target(?tar), 'patient profile'(?pp), 'diabetes mellitus'(?dm), 'drug subplan'(?dsp), 'asymptomatic patient'(?p), biguanide_contradictor(?p), sulfonylurea_contradictor(?p), thiazolidinedione_contradictor(?p), 'not DPP-4 inhibitor contradictor'(?p), oral(?or), has_diagnosis(?pp, ?dm), 'has beginning'(?i, ?i1), 'has duration description'(?i, ?dd), 'has end'(?i, ?i2), has_patient_profile(?p, ?pp), has_treatment_plan(?pp, ?tp), has_part(?tp, ?dsp), has_target(?tp, ?tar), months(?dd, 3), has_A1C_level(?tar, "6.5"^^xsd:double), has_previous_treatment_plan(?p, false), has_acceptance_level(?m, "accepted"), can_buy_expensive_drug(?p, true), DifferentFrom (?i1, ?i2) -> has_doseForm(?m, ?t), has_route_of_administration(?m, ?or), has_duration_backup(?tar, ?i), has_drug_participant(?dsp, ?m), has_dose(?m, 5), has_previous_treatment_plan(?p, true), has_drug_subplan_level(?dsp, "monotherapy")
166. 'impaired glucose tolerance'(?z1), patient(?x), 'treatment plan'(?tp), 'patient profile'(?y), 'physical activity'(?pa), 'lifestyle subplan'(?lp), 'aerobic exercise'(?ae), child(?x), 'days per week'(?dpw), 'minutes per day'(?aeu), has_diagnosis(?y, ?z1), has_patient_profile(?x, ?y), has_part(?tp, ?lp), has_aerobic_exercise(?pa, ?ae), has_lifestyle_participant(?lp, ?lfs) -> has_exercise_duration_UoM(?ae, ?aeu), has_exercise_frequency_UoM(?ae, ?dpw), has_exercise_duration(?ae, 60), has_intensity_percent(?ae, 50), has_exercise_frequency(?ae, 3)
167. ketoacidosis(?n), patient(?p), 'patient profile'(?f), has_patient_profile(?p, ?f), has_complication(?f, ?n) -> 'DPP-4 inhibitor contradictor'(?p)
168. ketoacidosis(?n), patient(?p), 'patient profile'(?f), has_patient_profile(?p, ?f), has_complication(?f, ?n) -> 'GLP-1 contradictor'(?p)
169. HbA1c(?f1), OGTT(?f2), 'impaired glucose tolerance'(?t2), 'millimoles per liter'(?mm2), percent(?mm), 'patient profile'(?r), 'asymptomatic patient'(?p), 'suitable for HbA1c test'(?p), has_lab_test(?r, ?f1), has_lab_test(?r, ?f2), has_measurement_unit(?f1, ?mu), has_measurement_unit(?f2, ?mu2), has_patient_profile(?p, ?r), has_quantitative_Value(?f1, ?q1), has_quantitative_Value(?f2, ?q2), swrlb:greaterThanOrEqual(?q1, "6.5"^^xsd:double), swrlb:greaterThanOrEqual(?q2, "7.8"^^xsd:double), swrlb:lessThanOrEqual(?q2, "11.0"^^xsd:double) -> has_diagnosis(?r, ?t2)
170. age(?a), 'NAFLD - Nonalcoholic fatty liver disease'(?z1), patient(?x), 'patient profile'(?y), has_demographic(?y, ?a), has_patient_profile(?x, ?y), has_complication(?y, ?z1), has_quantitative_Value(?a, ?v), swrlb:greaterThanOrEqual(?v, 45) -> 'symptomatic patient'(?x)
171. aripiprazole(?n), patient(?p), 'patient profile'(?f), has_patient_profile(?p, ?f), currently_taken_drug(?f, ?n) -> biguanide_contradictor(?p)
172. 'Duration description'(?dd), 'Time instant'(?i1), 'Time instant'(?i2), 'Proper interval'(?i), patient(?p), 'treatment plan'(?tp), tablet(?t), empagliflozin(?m), target(?tar), 'patient profile'(?pp), 'diabetes mellitus'(?dm), 'drug subplan'(?dsp), 'asymptomatic patient'(?p), biguanide_contradictor(?p), sulfonylurea_contradictor(?p), thiazolidinedione_contradictor(?p), 'DPP-4 inhibitor contradictor'(?p), not_SGLTI_contradictor(?p), oral(?or), has_diagnosis(?pp, ?dm), 'has beginning'(?i, ?i1), 'has duration description'(?i, ?dd), 'has end'(?i, ?i2), has_patient_profile(?p, ?pp), has_treatment_plan(?pp, ?tp), has_part(?tp, ?dsp), has_target(?tp, ?tar), months(?dd, 3), has_A1C_level(?tar, "6.5"^^xsd:double), has_previous_treatment_plan(?p, false), has_acceptance_level(?m, "accepted"), can_buy_expensive_drug(?p, true), DifferentFrom (?i1, ?i2) -> has_doseForm(?m, ?t), has_route_of_administration(?m, ?or), has_duration_backup(?tar, ?i), has_drug_participant(?dsp, ?m), has_dose(?m, 25), has_previous_treatment_plan(?p, true), has_drug_subplan_level(?dsp, "monotherapy")
173. age(?a), 'impaired fasting glycaemia'(?z1), patient(?x), 'patient profile'(?y), has_demographic(?y, ?a), has_patient_profile(?x, ?y), has_complication(?y, ?z1), has_quantitative_Value(?a, ?v), swrlb:greaterThanOrEqual(?v, 45) -> 'symptomatic patient'(?x)
174. 'kilogram per square meter'(?m), BMI(?a), patient(?x), 'patient profile'(?y), 'obese class II'(?u), has_physical_examination(?y, ?a), has_measurement_unit(?a, ?m), has_patient_profile(?x, ?y), has_quantitative_Value(?a, ?v), swrlb:greaterThanOrEqual(?v, "35.0"^^xsd:double), swrlb:lessThanOrEqual(?v, "39.9"^^xsd:double) -> has_demographic(?y, ?u)
175. atazanavir(?n), patient(?p), 'patient profile'(?f), has_patient_profile(?p, ?f), currently_taken_drug(?f, ?n) -> biguanide_contradictor(?p)
176. 'Duration description'(?dd), 'Time instant'(?i1), 'Time instant'(?i2), 'Proper interval'(?i), patient(?p), 'treatment plan'(?tp), tablet(?t), thiazolidinedione(?sul), metformin(?m), Glyburide(?tzd), 'drug timing'('while meal'), target(?tar), 'patient profile'(?pp), 'drug subplan'(?dsp), not_sulfonylurea_contradictor(?p), oral(?or), 'has beginning'(?i, ?i1), 'has duration description'(?i, ?dd), 'has end'(?i, ?i2), has_patient_profile(?p, ?pp), has_treatment_plan(?pp, ?tp), has_part(?tp, ?dsp), has_target(?tp, ?tar), has_drug_participant(?dsp, ?m), has_drug_participant(?dsp, ?sul), months(?dd, 3), has_A1C_level(?tar, "6.5"^^xsd:double), has_previous_treatment_plan(?p, true), has_target_achieved(?tp, false), has_drug_subplan_level(?dsup, "dual therapy"), has_passed_3_months(?tp, true), DifferentFrom (?i1, ?i2) -> has_doseForm(?tzd, ?t), has_route_of_administration(?tzd, ?or), when_to_take(?tzd, 'while meal'), has_duration_backup(?tar, ?i), has_drug_participant(?dsp, ?tzd), has_dose(?tzd, 5), has_previous_treatment_plan(?p, true), has_drug_subplan_level(?dsp, "triple therapy")
177. food(?f), patient(?x), 'patient profile'(?y), has_patient_profile(?x, ?y), has_complication(?y, ?z1), contradicte_with_disease_food(?f, ?z1) -> has_forbidden_food(?y, ?f)
178. RPG(?f), 'type 2 diabetes mellitus'(?t2), 'millimoles per liter'(?mm), 'patient profile'(?r), 'symptomatic patient'(?p), has_lab_test(?r, ?f), has_measurement_unit(?f, ?mu), has_patient_profile(?p, ?r), has_quantitative_Value(?f, ?q), swrlb:greaterThanOrEqual(?q, "11.1"^^xsd:double) -> has_diagnosis(?r, ?t2)
179. age(?a), dyslipidemia(?z1), patient(?x), 'patient profile'(?y), has_demographic(?y, ?a), has_patient_profile(?x, ?y), has_complication(?y, ?z1), has_quantitative_Value(?a, ?v), swrlb:greaterThanOrEqual(?v, 45) -> 'symptomatic patient'(?x)
180. age(?age), 'Duration description'(?dd), 'Time instant'(?i1), 'Time instant'(?i2), 'Proper interval'(?i), patient(?p), 'treatment plan'(?tp), tablet(?t), metformin(?m), 'drug timing'('while meal'), target(?tar), 'patient profile'(?pp), 'diabetes mellitus'(?dm), 'drug subplan'(?dsp), 'asymptomatic patient'(?p), not_biguanide_contradictor(?p), oral(?or), has_diagnosis(?pp, ?dm), 'has beginning'(?i, ?i1), 'has duration description'(?i, ?dd), 'has end'(?i, ?i2), has_patient_profile(?p, ?pp), has_treatment_plan(?pp, ?tp), has_part(?tp, ?dsp), has_target(?tp, ?tar), has_quantitative_Value(?age, ?val), months(?dd, 3), has_A1C_level(?tar, "6.5"^^xsd:double), has_previous_treatment_plan(?p, false), has_acceptance_level(?m, "accepted"), can_buy_low_price_drug(?p, true), swrlb:greaterThanOrEqual(?val, 10), DifferentFrom (?i1, ?i2) -> has_doseForm(?m, ?t), has_route_of_administration(?m, ?or), when_to_take(?m, 'while meal'), has_duration_backup(?tar, ?i), has_drug_participant(?dsp, ?m), has_dose(?m, 500), has_previous_treatment_plan(?p, true), has_drug_subplan_level(?dsp, "monotherapy")
181. patient(?p), 'patient profile'(?f), cimetidine(?n), has_patient_profile(?p, ?f), currently_taken_drug(?f, ?n) -> biguanide_contradictor(?p)
182. OGTT(?f), OGTT(?f2), 'type 2 diabetes mellitus'(?t2), 'millimoles per liter'(?mm), 'patient profile'(?r), 'asymptomatic patient'(?p), has_lab_test(?r, ?f), has_lab_test(?r, ?f2), has_measurement_unit(?f, ?mu), has_measurement_unit(?f2, ?mu), has_patient_profile(?p, ?r), has_quantitative_Value(?f, ?q), has_quantitative_Value(?f2, ?q2), swrlb:greaterThanOrEqual(?q, "11.1"^^xsd:double), swrlb:greaterThanOrEqual(?q2, "11.1"^^xsd:double), DifferentFrom (?f, ?f2) -> has_diagnosis(?r, ?t2)
183. 'Duration description'(?dd), 'Time instant'(?i1), 'Time instant'(?i2), 'Proper interval'(?i), patient(?p), 'treatment plan'(?tp), tablet(?t), thiazolidinedione(?sul), metformin(?m), canagliflozin(?tzd), 'drug timing'('while meal'), target(?tar), 'patient profile'(?pp), 'drug subplan'(?dsp), sulfonylurea_contradictor(?p), 'DPP-4 inhibitor contradictor'(?p), not_SGLTI_contradictor(?p), oral(?or), 'has beginning'(?i, ?i1), 'has duration description'(?i, ?dd), 'has end'(?i, ?i2), has_patient_profile(?p, ?pp), has_treatment_plan(?pp, ?tp), has_part(?tp, ?dsp), has_target(?tp, ?tar), has_drug_participant(?dsp, ?m), has_drug_participant(?dsp, ?sul), months(?dd, 3), has_A1C_level(?tar, "6.5"^^xsd:double), has_previous_treatment_plan(?p, true), has_target_achieved(?tp, false), has_drug_subplan_level(?dsup, "dual therapy"), has_passed_3_months(?tp, true), DifferentFrom (?i1, ?i2) -> has_doseForm(?tzd, ?t), has_route_of_administration(?tzd, ?or), when_to_take(?tzd, 'while meal'), has_duration_backup(?tar, ?i), has_drug_participant(?dsp, ?tzd), has_dose(?tzd, 300), has_previous_treatment_plan(?p, true), has_drug_subplan_level(?dsp, "triple therapy")
184. age(?a), 'anti-psychotic agent'(?z1), patient(?x), 'patient profile'(?y), has_demographic(?y, ?a), has_patient_profile(?x, ?y), currently_taken_drug(?y, ?z1), has_quantitative_Value(?a, ?v), swrlb:greaterThanOrEqual(?v, 45) -> 'symptomatic patient'(?x)
185. patient(?x), 'patient profile'(?y), 'lightly active'(?a), has_demographic(?y, ?a), has_patient_profile(?x, ?y), has_basal_metabolic_rate(?y, ?b), swrlb:multiply(?last, ?b, "1.375"^^xsd:double) -> has_total_calories(?y, ?last)
186. 'kilogram per square meter'(?m), BMI(?a), patient(?x), 'patient profile'(?y), 'obese class I'(?u), has_physical_examination(?y, ?a), has_measurement_unit(?a, ?m), has_patient_profile(?x, ?y), has_quantitative_Value(?a, ?v), swrlb:greaterThanOrEqual(?v, "30.0"^^xsd:double), swrlb:lessThanOrEqual(?v, "34.9"^^xsd:double) -> has_demographic(?y, ?u)
187. gender(?g), patient(?p), 'pregnancy state'(?pr), 'patient profile'(?f), has_demographic(?f, ?g), has_demographic(?f, ?pr), has_patient_profile(?p, ?f), has_qualitative_value(?g, "female"), has_qualitative_value(?pr, "pregnant") -> sulfonylurea_contradictor(?p)
188. iloperidone(?n), patient(?p), 'patient profile'(?f), has_patient_profile(?p, ?f), currently_taken_drug(?f, ?n) -> biguanide_contradictor(?p)
189. age(?a), 'bipolar disorder'(?z1), patient(?x), 'patient profile'(?y), has_demographic(?y, ?a), has_patient_profile(?x, ?y), has_complication(?y, ?z1), has_quantitative_Value(?a, ?v), swrlb:greaterThanOrEqual(?v, 45) -> 'symptomatic patient'(?x)
190. age(?a), retinopathy(?z1), patient(?x), 'patient profile'(?y), has_demographic(?y, ?a), has_patient_profile(?x, ?y), has_complication(?y, ?z1), has_quantitative_Value(?a, ?v), swrlb:greaterThanOrEqual(?v, 45) -> 'symptomatic patient'(?x)
191. patient(?p), 'patient profile'(?f), dichlorphenamide(?n), has_patient_profile(?p, ?f), currently_taken_drug(?f, ?n) -> biguanide_contradictor(?p)
192. HbA1c(?f), HbA1c(?f2), 'type 2 diabetes mellitus'(?t2), percent(?mm), 'patient profile'(?r), 'asymptomatic patient'(?p), has_lab_test(?r, ?f), has_lab_test(?r, ?f2), has_measurement_unit(?f, ?mu), has_measurement_unit(?f2, ?mu), has_patient_profile(?p, ?r), has_quantitative_Value(?f, ?q), has_quantitative_Value(?f2, ?q2), swrlb:greaterThanOrEqual(?q, "6.5"^^xsd:double), swrlb:greaterThanOrEqual(?q2, "6.5"^^xsd:double), DifferentFrom (?f, ?f2) -> has_diagnosis(?r, ?t2)
193. FPG(?f1), FPG(?f2), 'type 2 diabetes mellitus'(?t2), 'millimoles per liter'(?mm), 'patient profile'(?r), 'asymptomatic patient'(?p), has_lab_test(?r, ?f1), has_lab_test(?r, ?f2), has_measurement_unit(?f1, ?mu), has_measurement_unit(?f2, ?mu), has_patient_profile(?p, ?r), has_quantitative_Value(?f1, ?q1), has_quantitative_Value(?f2, ?q2), swrlb:greaterThanOrEqual(?q1, "7.0"^^xsd:double), swrlb:greaterThanOrEqual(?q2, "7.0"^^xsd:double), DifferentFrom (?f1, ?f2) -> has_diagnosis(?r, ?t2)
194. 'impaired glucose tolerance'(?z1), patient(?x), 'treatment plan'(?tp), 'patient profile'(?y), 'physical activity'(?pa), 'lifestyle subplan'(?lp), 'aerobic exercise'(?ae), teenager(?x), 'days per week'(?dpw), 'minutes per day'(?aeu), has_diagnosis(?y, ?z1), has_patient_profile(?x, ?y), has_part(?tp, ?lp), has_aerobic_exercise(?pa, ?ae), has_lifestyle_participant(?lp, ?lfs) -> has_exercise_duration_UoM(?ae, ?aeu), has_exercise_frequency_UoM(?ae, ?dpw), has_exercise_duration(?ae, 60), has_intensity_percent(?ae, 50), has_exercise_frequency(?ae, 3)
195. ketoacidosis(?n), patient(?p), 'patient profile'(?f), has_patient_profile(?p, ?f), has_complication(?f, ?n) -> biguanide_contradictor(?p)
196. RPG(?f1), RPG(?f2), 'type 2 diabetes mellitus'(?t2), 'millimoles per liter'(?mm), 'patient profile'(?r), 'asymptomatic patient'(?p), has_lab_test(?r, ?f1), has_lab_test(?r, ?f2), has_measurement_unit(?f1, ?mu), has_measurement_unit(?f2, ?mu), has_patient_profile(?p, ?r), has_quantitative_Value(?f1, ?q1), has_quantitative_Value(?f2, ?q2), swrlb:greaterThanOrEqual(?q1, "11.1"^^xsd:double), swrlb:greaterThanOrEqual(?q2, "11.1"^^xsd:double), DifferentFrom (?f1, ?f2) -> has_diagnosis(?r, ?t2)
197. gender(?g), patient(?p), 'pregnancy state'(?pr), 'patient profile'(?f), has_demographic(?f, ?g), has_demographic(?f, ?pr), has_patient_profile(?p, ?f), has_qualitative_value(?g, "female"), has_qualitative_value(?pr, "pregnant") -> thiazolidinedione_contradictor(?p)
198. 'gastroparesis syndrome'(?n), patient(?p), 'patient profile'(?f), has_patient_profile(?p, ?f), has_complication(?f, ?n) -> incretin_contradictor(?p)
199. FPG(?f1), OGTT(?f2), 'impaired glucose tolerance'(?t2), 'millimoles per liter'(?mm), 'patient profile'(?r), 'asymptomatic patient'(?p), has_lab_test(?r, ?f1), has_lab_test(?r, ?f2), has_measurement_unit(?f1, ?mu), has_measurement_unit(?f2, ?mu), has_patient_profile(?p, ?r), has_quantitative_Value(?f1, ?q1), has_quantitative_Value(?f2, ?q2), swrlb:greaterThanOrEqual(?q1, "6.1"^^xsd:double), swrlb:greaterThanOrEqual(?q2, "7.8"^^xsd:double), swrlb:lessThanOrEqual(?q1, "6.9"^^xsd:double), swrlb:lessThanOrEqual(?q2, "11.0"^^xsd:double) -> has_diagnosis(?r, ?t2)
200. 'Duration description'(?dd), 'Time instant'(?i1), 'Time instant'(?i2), 'Proper interval'(?i), patient(?p), 'treatment plan'(?tp), tablet(?t), rosiglitazone(?m), target(?tar), 'patient profile'(?pp), 'diabetes mellitus'(?dm), 'drug subplan'(?dsp), 'asymptomatic patient'(?p), biguanide_contradictor(?p), sulfonylurea_contradictor(?p), not_thiazolidinedione_contradictor(?p), oral(?or), has_diagnosis(?pp, ?dm), 'has beginning'(?i, ?i1), 'has duration description'(?i, ?dd), 'has end'(?i, ?i2), has_patient_profile(?p, ?pp), has_treatment_plan(?pp, ?tp), has_part(?tp, ?dsp), has_target(?tp, ?tar), months(?dd, 3), has_A1C_level(?tar, "6.5"^^xsd:double), has_previous_treatment_plan(?p, false), has_acceptance_level(?m, "accepted"), can_buy_low_price_drug(?p, true), DifferentFrom (?i1, ?i2) -> has_doseForm(?m, ?t), has_route_of_administration(?m, ?or), has_duration_backup(?tar, ?i), has_drug_participant(?dsp, ?m), has_dose(?m, 4), has_previous_treatment_plan(?p, true), has_drug_subplan_level(?dsp, "monotherapy")
201. 'hepatic failure'(?z1), patient(?x), 'patient profile'(?y), has_patient_profile(?x, ?y), has_complication(?y, ?z1) -> 'misleaded with HbA1c'(?x)
202. 'Duration description'(?dd), 'Time instant'(?i1), 'Time instant'(?i2), 'Proper interval'(?i), patient(?p), 'treatment plan'(?tp), tablet(?t), thiazolidinedione(?sul), metformin(?m), sitagliptin(?tzd), 'drug timing'('while meal'), target(?tar), 'patient profile'(?pp), 'drug subplan'(?dsp), sulfonylurea_contradictor(?p), 'not DPP-4 inhibitor contradictor'(?p), oral(?or), 'has beginning'(?i, ?i1), 'has duration description'(?i, ?dd), 'has end'(?i, ?i2), has_patient_profile(?p, ?pp), has_treatment_plan(?pp, ?tp), has_part(?tp, ?dsp), has_target(?tp, ?tar), has_drug_participant(?dsp, ?m), has_drug_participant(?dsp, ?sul), months(?dd, 3), has_A1C_level(?tar, "6.5"^^xsd:double), has_previous_treatment_plan(?p, true), has_target_achieved(?tp, false), has_drug_subplan_level(?dsup, "dual therapy"), has_passed_3_months(?tp, true), DifferentFrom (?i1, ?i2) -> has_doseForm(?tzd, ?t), has_route_of_administration(?tzd, ?or), when_to_take(?tzd, 'while meal'), has_duration_backup(?tar, ?i), has_drug_participant(?dsp, ?tzd), has_dose(?tzd, 100), has_previous_treatment_plan(?p, true), has_drug_subplan_level(?dsp, "triple therapy")
203. age(?a), neuropathy(?z1), patient(?x), 'patient profile'(?y), has_demographic(?y, ?a), has_patient_profile(?x, ?y), has_complication(?y, ?z1), has_quantitative_Value(?a, ?v), swrlb:greaterThanOrEqual(?v, 45) -> 'symptomatic patient'(?x)
204. ketoacidosis(?n), patient(?p), 'patient profile'(?f), has_patient_profile(?p, ?f), has_complication(?f, ?n) -> thiazolidinedione_contradictor(?p)
205. 'Duration description'(?dd), 'Time instant'(?i1), 'Time instant'(?i2), 'Proper interval'(?i), patient(?p), 'treatment plan'(?tp), tablet(?t), Glipizide(?m), target(?tar), 'patient profile'(?pp), 'diabetes mellitus'(?dm), 'drug subplan'(?dsp), 'asymptomatic patient'(?p), biguanide_contradictor(?p), not_sulfonylurea_contradictor(?p), oral(?or), has_diagnosis(?pp, ?dm), 'has beginning'(?i, ?i1), 'has duration description'(?i, ?dd), 'has end'(?i, ?i2), has_patient_profile(?p, ?pp), has_treatment_plan(?pp, ?tp), has_part(?tp, ?dsp), has_target(?tp, ?tar), months(?dd, 3), has_A1C_level(?tar, "6.5"^^xsd:double), has_previous_treatment_plan(?p, false), has_acceptance_level(?m, "accepted"), can_buy_low_price_drug(?p, true), DifferentFrom (?i1, ?i2) -> has_doseForm(?m, ?t), has_route_of_administration(?m, ?or), when_to_take(?m, 'while meal'), has_duration_backup(?tar, ?i), has_drug_participant(?dsp, ?m), has_dose(?m, 10), has_previous_treatment_plan(?p, true), has_drug_subplan_level(?dsp, "monotherapy")
206. patient(?p), 'patient profile'(?f), ciprofloxacin(?n), has_patient_profile(?p, ?f), currently_taken_drug(?f, ?n) -> biguanide_contradictor(?p)
207. 'Duration description'(?dd), 'Time instant'(?i1), 'Time instant'(?i2), 'Proper interval'(?i), patient(?p), 'treatment plan'(?tp), tablet(?t), saxagliptin(?m), target(?tar), 'patient profile'(?pp), 'diabetes mellitus'(?dm), 'drug subplan'(?dsp), 'asymptomatic patient'(?p), biguanide_contradictor(?p), sulfonylurea_contradictor(?p), thiazolidinedione_contradictor(?p), 'not DPP-4 inhibitor contradictor'(?p), oral(?or), has_diagnosis(?pp, ?dm), 'has beginning'(?i, ?i1), 'has duration description'(?i, ?dd), 'has end'(?i, ?i2), has_patient_profile(?p, ?pp), has_treatment_plan(?pp, ?tp), has_part(?tp, ?dsp), has_target(?tp, ?tar), months(?dd, 3), has_A1C_level(?tar, "6.5"^^xsd:double), has_previous_treatment_plan(?p, false), has_acceptance_level(?m, "accepted"), can_buy_expensive_drug(?p, true), DifferentFrom (?i1, ?i2) -> has_doseForm(?m, ?t), has_route_of_administration(?m, ?or), has_duration_backup(?tar, ?i), has_drug_participant(?dsp, ?m), has_dose(?m, 5), has_previous_treatment_plan(?p, true), has_drug_subplan_level(?dsp, "monotherapy")
208. levofloxacin(?n), patient(?p), 'patient profile'(?f), has_patient_profile(?p, ?f), currently_taken_drug(?f, ?n) -> biguanide_contradictor(?p)
209. food(?f), 'diabetes drug'(?dr), patient(?x), 'treatment plan'(?tp), 'patient profile'(?y), 'drug subplan'(?dsp), contradicte_with_food(?dr, ?f), has_patient_profile(?x, ?y), has_part(?tp, ?dsp), has_drug_participant(?dsp, ?dr) -> has_forbidden_food(?y, ?f)
210. HbA1c(?a1c), patient(?p), 'treatment plan'(?tp), target(?tg), 'patient profile'(?pp), has_lab_test(?pp, ?a1c), has_patient_profile(?p, ?pp), has_treatment_plan(?pp, ?tp), has_target(?tp, ?tg), has_quantitative_Value(?a1c, ?v2), has_A1C_level(?tg, ?v1), has_date(?a1c, ?dat1), has_date(?tp, ?dat2), swrlb:greaterThan(?dat1, ?dat2), swrlb:lessThanOrEqual(?v2, ?v1) -> has_target_achieved(?tp, true)
211. patient(?p), 'patient profile'(?f), ioversol(?n), has_patient_profile(?p, ?f), currently_taken_drug(?f, ?n) -> biguanide_contradictor(?p)
212. patient(?p), 'patient profile'(?f), digoxin(?n), has_patient_profile(?p, ?f), currently_taken_drug(?f, ?n) -> biguanide_contradictor(?p)
213. salsalate(?n), patient(?p), 'patient profile'(?f), has_patient_profile(?p, ?f), currently_taken_drug(?f, ?n) -> sulfonylurea_contradictor(?p)
214. RPG(?f), OGTT(?f2), 'millimoles per liter'(?mm), 'normal patient'(?t2), 'patient profile'(?r), 'asymptomatic patient'(?p), has_lab_test(?r, ?f), has_lab_test(?r, ?f2), has_measurement_unit(?f, ?mu), has_measurement_unit(?f2, ?mu), has_patient_profile(?p, ?r), has_quantitative_Value(?f, ?q), has_quantitative_Value(?f2, ?q2), swrlb:greaterThanOrEqual(?q, "11.1"^^xsd:double), swrlb:greaterThanOrEqual(?q2, "11.1"^^xsd:double) -> has_diagnosis(?r, ?t2)
